# Characterization of oligopeptide patterns in large protein sets

**DOI:** 10.1186/1471-2164-8-346

**Published:** 2007-10-01

**Authors:** Anders Bresell, Bengt Persson

**Affiliations:** 1 IFM Bioinformatics, Linköping University, S-581 83 Linköping, Sweden; 2 Department of Cell and Molecular Biology (CMB), Karolinska Institutet, S-171 77 Stockholm, Sweden

## Abstract

**Background:**

Recent sequencing projects and the growth of sequence data banks enable oligopeptide patterns to be characterized on a genome or kingdom level. Several studies have focused on kingdom or habitat classifications based on the abundance of short peptide patterns. There have also been efforts at local structural prediction based on short sequence motifs. Oligopeptide patterns undoubtedly carry valuable information content. Therefore, it is important to characterize these informational peptide patterns to shed light on possible new applications and the pitfalls implicit in neglecting bias in peptide patterns.

**Results:**

We have studied four classes of pentapeptide patterns (designated POP, NEP, ORP and URP) in the kingdoms archaea, bacteria and eukaryotes. POP are highly abundant patterns statistically not expected to exist; NEP are patterns that do not exist but are statistically expected to; ORP are patterns unique to a kingdom; and URP are patterns excluded from a kingdom. We used two data sources: the *de facto *standard of protein knowledge Swiss-Prot, and a set of 386 completely sequenced genomes. For each class of peptides we looked at the 100 most extreme and found both known and unknown sequence features. Most of the known sequence motifs can be explained on the basis of the protein families from which they originate.

**Conclusion:**

We find an inherent bias of certain oligopeptide patterns in naturally occurring proteins that cannot be explained solely on the basis of residue distribution in single proteins, kingdoms or databases. We see three predominant categories of patterns: (i) patterns widespread in a kingdom such as those originating from respiratory chain-associated proteins and translation machinery; (ii) proteins with structurally and/or functionally favored patterns, which have not yet been ascribed this role; (iii) multicopy species-specific retrotransposons, only found in the genome set. These categories will affect the accuracy of sequence pattern algorithms that rely mainly on amino acid residue usage. Methods presented in this paper may be used to discover targets for antibiotics, as we identify numerous examples of kingdom-specific antigens among our peptide classes. The methods may also be useful for detecting coding regions of genes.

## Background

Sequencing projects have been ongoing for decades and have made enormous amounts of sequence data available. This opens up possibilities for large-scale investigations of oligopeptide pattern frequencies, both in general and on a kingdom or genome level by relying on statistically impressive amounts of data. For example, kingdoms can be classified on the basis of tripeptide pattern abundances using only the first two principal components, and the compositional signatures can be explained by habitats [1]. However, at the level of relative amino acid composition, one can see a connection with growth temperature [2]. In another study, the occurrence of oligopeptides of lengths three, four and five was investigated using the NCBI non-redundant sequence database, showing that many peptide patterns did not exist. Six non-existent pentapeptides were synthesized and expressed as parts of a soluble fusion protein in reasonably high yields, suggesting that oligopeptide patterns in proteins are selected on an evolutionary basis rather than by limitations in the biosynthetic pathway [3]. It has also been shown that short amino acid residue patterns can be useful for predicting sequence features, *e.g*. secondary structure prediction using pentapeptides [4]. Furthermore, efforts at local structure prediction have been made with sequence segments of length nine using profiles based on structurally aligned regions [5]. Among the best-known initiatives is the Prosite pattern database, which has been used for many years in protein sequence annotation for assigning function and structure via regular expressions [6]. Consequently, it is beyond doubt that short oligopeptide patterns carry information and that many patterns are either over- or under-represented.

Many common bioinformatic methods of today, *e.g*. BLAST, hidden Markov models, PSI-BLAST and Prosite scans, assume that the relative amino acid residue frequency is more or less the same for all larger protein data sets. However, if we have a database biased for a certain species, a kingdom or a set of protein families, then over- and under-represented oligopeptide patterns will cause overestimation of the accuracy of the result, for which an amino acid null frequency model will not account. Also, besides utilizing kingdom-specific peptide patterns for diagnostics, they can be used to find antigens and targets for antibiotics. It might also be possible to find patterns with high risk of causing autoantigens in eukaryotes after viral or bacterial infections.

In this study, we have performed a large-scale investigation of all possible combinations of five amino acid residues, pentapetides, in order to characterize oligopeptide patterns that are over- or under-represented in general or with respect to a kingdom. We find not only sequence patterns of known and frequently-used features but also patterns due to compositional bias. In addition, we find novel patterns which might be part of features not revealed by current bioinformatic methods, forming structural building blocks or segments selectively filtered because of unfavorable properties or immune response-induced epitopes.

## Results and discussion

### Data sets

We have searched in protein databases for pentapeptide patterns that are over- or under-represented. On one hand, we wanted to utilize as much sequence data as presently available. For this, we collected all protein sequences from 386 completed genomes, in the following referred to as the genome set. On the other hand, we wanted well annotated data in order to get information about the proteins. For this, we utilized the Swiss-Prot part of UniprotKB [7,8], hereafter referred to as Swiss-Prot. We decided to use both these two data sets, since they complement each other. The Swiss-Prot database has high quality, is very well annotated and constitutes the current *de facto *standard of protein knowledge. Swiss-Prot contains many well-characterized proteins and one may suspect a bias because it is easier to characterize proteins that are easily purified and/or homologous to proteins that are already well known. The genome sequence set, on the other hand, represents a more complete and unbiased distribution with respect to different types of proteins. However, many sequences in the genome set have been predicted by unsupervised automatic high-throughput algorithms and hence might be of lower quality than those in the Swiss-Prot dataset. There might also be a bias towards organisms of medical and biotechnological interest. In addition, genomes might contain duplicated genes. Consequently, the two datasets have different properties which motivates their combined utilization in this investigation.

Initially, we compared sequence patterns in the observed data sets with those in the randomized data sets. The randomization was performed so that for each protein in the dataset, a randomized sequence was generated with the same total amino acid residue content as the original sequence, but with the residues in an arbitrary order (cf. the Methods section for details). The oligopeptide patterns of lengths four, five and six in the original and randomized data sets show that the gap between observed and expected numbers of patterns increases with pattern length (Table 1). Evidently, there is an inherent bias towards certain oligopeptide patterns in naturally-occurring proteins that cannot be explained solely on the basis of residue distribution in single proteins, kingdoms or data banks. Notably, all combinations of oligopeptides of size four exist in both the original and randomized sets, except for Swiss-Prot-original, from which one peptide pattern was missing. However, peptides with zero observations were found in all sets of lengths five and six (including randomized). Furthermore, for penta- and hexapeptides the observed number of different patterns is fewer in original data compared with randomized data (Table 1).

**Table 1:** Number of different observed oligopeptides

Length (*d*)	Theoretical (20^*d*^)	Swiss-Prot		Genome	
		original	randomized	original	randomized

4	160 000	159 999	160 000	160 000	160 000
5	3 200 000	3 021 259	3 136 980	3 196 081	3 199 490
6	64 000 000	25 025 493	34 155 965	52 989 609	58 435 452

Number of different observed oligopeptide patterns of lengths four, five and six in the original and randomized data sets.

The oligopeptide sets of length four, five and six overlap in informational content because an oligopeptide set of length six is a partitioning of the set of length five, and that of length five is a partitioning of the set of length four. We limited our further studies to only one length, pentapeptides, which proved to be a good compromise between informational resolution and run times for the computer calculations.

**Figure 1:**
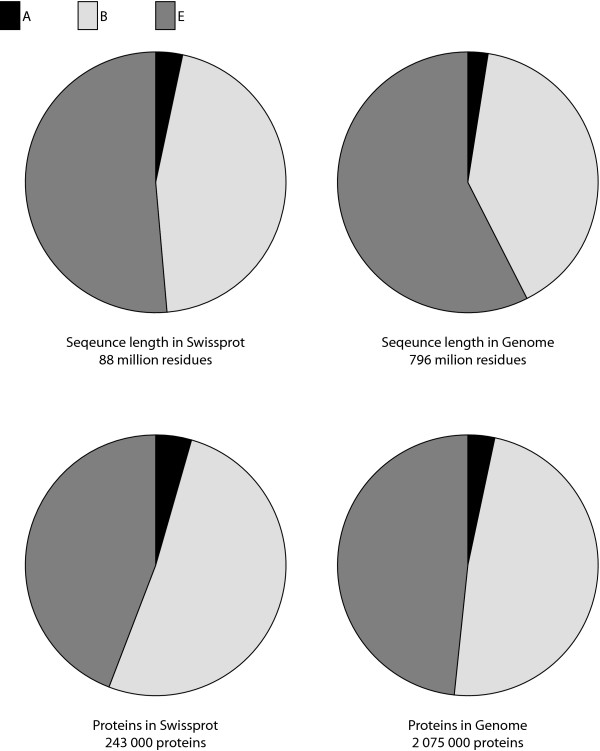
**Kingdom distributions of protein number and sequence length**. The pie charts show the total sequence length and the number of proteins found in the kingdoms of archaea (A), bacteria (B) and eukaryotes (E). The fraction of proteins in bacteria and eukaryotes are approximately the same, but the eukaryotic proteins are 8% longer on average in the genome set compared to Swiss-Prot. The archaeal portion constitutes only 2–5% of the data and will in absolute number of observations therefore be considerably lower.

Figure 1 shows pie charts of sequence lengths and number of proteins for each kingdom and data set. The genome set is more than eight times larger than the Swiss-Prot set, both in terms of total sequence length and in number of proteins. For eukaryotes, the average sequence length is 8% longer in the genome set than in the Swiss-Prot set. This, together with the fact that archaeal, bacterial and eukaryotic sequence data are of different orders of magnitude, must be borne in mind when interpreting the peptide patterns frequencies.

### Classification of peptide patterns sets

In order to investigate various biological aspects of the nature of peptide patterns, we create four categories and focus on the 100 most extreme examples in each category. The categories are: (i) POP ("positively selected peptides"), which are the most abundant peptide patterns in observed data and are found not at all or only occasionally in randomized data; (ii) NEP ("negatively selected peptides"), which are those with extremely low abundance in available protein data but with high frequencies in randomized data; (iii) ORP ("over-represented peptides") are the most frequent kingdom-specific peptide patterns; and (iv) URP ("under-represented peptides") are those with extremely low abundance in a particular kingdom. POPs are expected to contain favored structural or functional motifs and might also belong to large protein families. They are expected in low numbers in view of their amino acid compositions but are in fact over-represented and must therefore result from positive selective pressure. ORPs are unique to a kingdom and might be used as diagnostic patterns. They will cause bias in databases that do not have equal portions of proteins from the three kingdoms. NEPs are expected to result from negative selective pressure and can be explained as structurally unfavored building blocks. URPs can be parts of epitopes that are inappropriate to the kingdom or avoided for other reasons and, as for the ORPs, this will lead to bias in protein databases.

**Figure 2:**
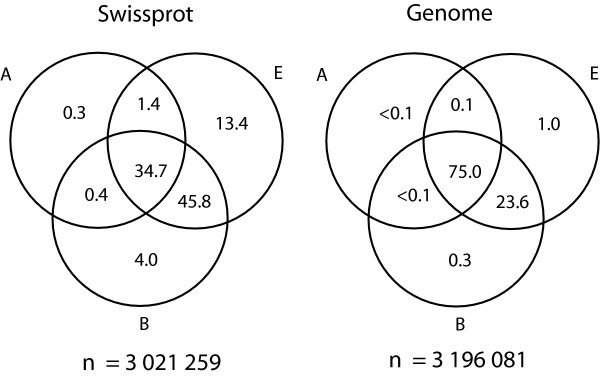
**Peptide patterns among kingdoms**. The Venn diagrams show the percentage of peptide patterns common to the kingdoms in the original sequence sets. Only few peptide patterns are unique to a kingdom in the genome data set. As many as 98.7% of the peptide patterns are common to two or more kingdoms in the genome set, while the corresponding number for Swiss-Prot is 82.3%. *<*0.1 indicates less than 0.1%. The sum for each data set is 100%.

When analyzing ORPs, URPs, POPs and NEPs (see below) it is interesting to relate the overall abundance of pentapeptides in the data sets. The Venn diagrams in Figure 2 show the percentages of peptide patterns common among the kingdoms. In the genome set, as many as 75.0% of the peptide patterns are in common to all three kingdoms, but in Swiss-Prot this fraction is only 34.7%; there are more bacterial, eukaryotic and bacterial-eukaryotic specific patterns in the latter.

### Amino acid residue composition

The overall relative amino acid compositions for each kingdom in the two data sets are shown in Additional file 1, ordered by their average frequencies in the respective data set. The data follows trends in previous studies, *i.e*. our data are not contradictory to a habitat-amino acid usage correlation study [2] and consistent with kingdom classification via principle component analysis [1]. Only small differences are observed between Swiss-Prot and genome data sets.

**Figure 3:**
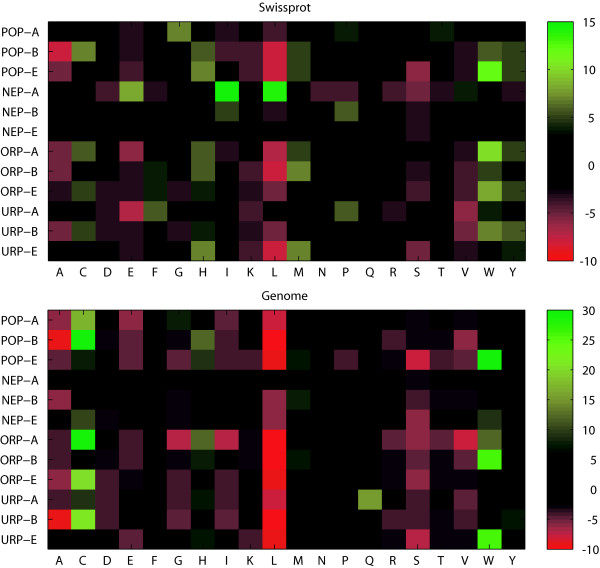
**Amino acid residue differences in peptide sets and kingdoms**. Graphics shows the differences in percentage points *versus *the overall residue frequencies in the respective kingdom (cf. Additional file 1). All peptides sets contain 100 peptide patterns, except ORP-A for which only 54 and 6 patterns passed the filtering step for the Swiss-Prot and genome datasets, respectively (cf. Table 3). Green indicates that the residue is more abundant than background, while red indicates less abundant than background. The difference in percentage points is shown by color intensities according to the scale.

The differences (in percentage points) in amino acid usage between the peptide pattern classes and the overall relative frequencies in respect of kingdom/dataset are shown in Figure 3. Although these peptide classes comprise only the 100 most extreme patterns in each category, a few deviations are worth mentioning. Archaeal Swiss-Prot NEPs have high levels of isoleucine and leucine, amino acid residues that are chemically similar and hydrophobic. Leucine is less frequent in bacterial and eukaryotic POPs, bacterial ORPs and eukaryotic URPs. Tryptophan is greatly elevated in eukaryotic Swiss-Prot POPs, especially considering its low frequency in general. This residue type is also enriched in archaeal ORPs. In the genome set, we also see elevated levels of tryptophan in eukaryotic POPs, bacterial ORPs and eukaryotic URPs. Furthermore, cysteines in the genome set are highly elevated in bacterial POPs and archaeal ORPs, but also in eukaryotic ORPs and bacterial URPs. Tryptophan and cysteine are the two rarest amino acid residues and when peptide patterns are selected on the basis of background frequencies, as in this study, it is not unexpected that such patterns are rich in these residues. However, cysteine and tryptophan are also often structurally and functionally important, *e.g*. at active sites. Cysteine can form intra- or inter-molecular disulfide bridges and multiple cysteines can coordinate metal ions. Furthermore, proteins with redox-sensitive cysteines can regulate other proteins dependent upon the redox state [9]. Tryptophan is often found at the membrane-water interface in membranes [10] and has been shown important in protein folding [11]. Tryptophan is also often found in anti-microbial peptides [12]. Furthermore, we see low levels of serine and valine in eukaryotic POPs and archaeal ORPs, respectively, and elevated levels of glutamine in archaeal URPs. Leucine shows a pronounced general trend towards negative selection among the peptide categories. Thus, even if leucine is the most frequent residue type, it is not over- or under-represented in the peptide categories, with the exception of Swiss-Prot archaeal NEPs.

### Feature analysis of peptide patterns

In order to categorize a pattern as known or novel we investigated its occurrence in Swiss-Prot sequence features. If at least 20% of the sequence hits of a pattern were part of the same feature, we annotated the feature to the peptide pattern. In the following sections, ambiguous features such as "chain", "domain", "topological domain" and "region" are discarded. A summary of the number of novel and known patterns per peptide pattern set is shown in Table 2. Interesting patterns within each of the four categories POP, NEP, ORP and URP are described in the subsequent paragraphs and listed in Additional file 2. Complete lists of top 100 peptides of each category and kingdom are given in Additional files 3 and 4.

**Table 2:** Swiss-Prot sequence features matching peptide patterns

Dataset	wf	wof	Feature (Number of peptide patterns)
POP-A-Swissprot	46	54	NP_BIND 25, ACT_SITE 7, REPEAT 6, MOTIF 4, BINDING 4, ZN_FING 3, METAL 2
POP-A-genome	52	48	METAL 22, ZN_FING 15, NP_BIND 7, ACT_SITE 3, DISULFID 3, CARBOHYD 2, VAR_SEQ 2, LIPID 1, SIGNAL 1, MUTAGEN 1, REPEAT 1
POP-B-Swissprot	13	87	METAL 6, ACT_SITE 5, MOTIF 2, NP_BIND 1
POP-B-genome	67	33	METAL 33, ZN_FING 18, DISULFID 7, BINDING 6, ACT_SITE 4, TRANSMEM 2, VAR_SEQ 2, STRAND 2, REPEAT 1, SE_CYS 1, SITE 1, TURN 1, HELIX 1
POP-E-Swissprot	24	76	METAL 10, TRANSMEM 5, DNA_BIND 5, DISULFID 3, ZN_FING 2, BINDING 1, ACT_SITE 1
POP-E-genome	39	61	TRANSMEM 10, ZN_FING 6, DISULFID 6, REPEAT 5, VAR_SEQ 4, COMPBIAS 2, METAL 2, CARBOHYD 2, VARIANT 1, BINDING 1, PROPEP 1, CONFLICT 1
NEP-A-Swissprot	16	84	TRANSMEM 16
NEP-A-genome	6	94	TRANSMEM 5, PEPTIDE 1
NEP-B-Swissprot	10	90	TRANSMEM 7, VAR_SEQ 1, COMPBIAS 1, COILED 1
NEP-B-genome	38	62	DISULFID 12, VAR_SEQ 8, REPEAT 6, TRANSMEM 5, COMPBIAS 3, METAL 2, ZN_FING 2, VARIANT 1, COILED 1, STRAND 1
NEP-E-Swissprot	7	93	TRANSMEM 3, VAR_SEQ 2, COMPBIAS 1, NP_BIND 1
NEP-E-genome	14	86	TRANSMEM 4, DISULFID 4, STRAND 3, ZN_FING 1, MOTIF 1, PROPEP 1
ORP-A-Swissprot	10	44	METAL 3, BINDING 2, ACT_SITE 1, DISULFID 1, MOTIF 1, TRANSMEM 1, HELIX 1
ORP-A-genome	0	6	
ORP-B-Swissprot	7	93	METAL 4, BINDING 3, NP_BIND 1
ORP-B-genome	29	71	TRANSMEM 17, STRAND 4, HELIX 3, METAL 2, DNA_BIND 1, ACT_SITE 1, TURN 1, ZN_FING 1, DISULFID 1, CROSSLNK 1
ORP-E-Swissprot	24	76	METAL 10, ZN_FING 9, DNA_BIND 3, NP_BIND 1, TRANSMEM 1
ORP-E-genome	40	60	ZN_FING 22, DISULFID 5, COMPBIAS 2, REPEAT 2, CARBOHYD 2, TRANSMEM 2, LIPID 1, DNA_BIND 1, HELIX 1, COILED 1, VAR_SEQ 1, PROPEP 1
URP-A-Swissprot	13	87	COMPBIAS 7, METAL 4, VAR_SEQ 3, ZN_FING 2, REPEAT 1, COILED 1
URP-A-genome	79	21	ZN_FING 49, COMPBIAS 10, TRANSMEM 6, DISULFID 6, REPEAT 3, DNA_BIND 2, SIGNAL 2, ACT_SITE 2, NP_BIND 1, METAL 1, CARBOHYD 1, VAR_SEQ 1
URP-B-Swissprot	23	77	ZN_FING 10, METAL 8, DNA_BIND 3, NP_BIND 1, TRANSMEM 1
URP-B-genome	47	53	ZN_FING 24, DISULFID 9, COMPBIAS 2, TRANSMEM 2, HELIX 2, CARBOHYD 2, REPEAT 1, SIGNAL 1, METAL 1 TURN 1, NP_BIND 1, COILED 1, PROPEP 1, LIPID 1
URP-E-Swissprot	6	94	NP_BIND 2, METAL 2, BINDING 1, ACT_SITE 1
URP-E-genome	31	69	TRANSMEM 19, HELIX 4, STRAND 4, METAL 2, TURN 2, DNA_BIND 1, ACT_SITE 1, ZN_FING 1, CROSSLNK 1

Known Swiss-Prot sequence features of peptide patterns were retrieved by matching peptide patterns against Swiss-Prot entries. Features that matched fewer than 20% of the sequence hits of a peptide pattern or were ambiguous (see Methods section) were excluded. The table shows the fraction of feature-associated patterns for each peptide set and the respective number of patterns associated with each feature. Individual data on each pattern are given in Additional files 3 and 4. wf, with feature, number of peptide patterns that matches a sequence feature type in at least 20% of all the sequence hits in Swiss-Prot (release 51.5); wof, without feature, number of peptide patterns that does not meet the criteria of wf.

### POP – positively selected and structurally/functionally favored peptide patterns

The POP category contains peptide patterns that are expected not to exist but are in fact found in large numbers. These peptides have intrinsically favorable properties and have undergone positive selection, presumably containing structurally or functionally important sequences. Only for few of the patterns, the databases contain information about properties, while for the majority of these patterns, functional assignments still remains to be done. Here we summarize selected findings about the most interesting patterns among the 100 most frequent peptides for each kingdom. All these patterns were found at most twice in the randomized data sets but between 28 and 1648 times in the observed data sets, so they are statistically unexpected. The patterns can be divided into three groups: a) large protein families, b) peptides with unassigned functions, and c) integrases and transposases.

#### Large protein families

More than half of archaeal Swiss-Prot POP share the nucleotide phosphate-binding feature, patterns that are also found in several thousand copies in other kingdoms in the Swiss-Prot data set. For the genome set we see other dominant characters, "zinc finger" and "metal". Only 13% of bacterial Swiss-Prot POPs are associated with a known feature, "metal" and "active site" being the most frequent, with examples from glutamine amidotransferases, the methionine import ATP-binding protein metN, the GTP-binding protein lepA and elongation factor G. The bacterial POPs from the genome set show many more feature associations (67%) than their Swiss-Prot counterparts, "metal" and "zinc finger" predominating. However, the most abundant peptide pattern WCGPC (599 occurrences), which is found in almost all bacterial species in the genome data set, has the features "disulfide" and "active site" and is found in thioredoxins [13]. In the eukaryotic Swiss-Prot POP set, frequent peptide patterns originate from cytochrome b, homeobox associated proteins, and various sodium channels.

#### Peptides with unassigned functions

In the archaeal genome set, the third most abundant peptide pattern, CPVCG (258 occurrences), is not part of any known feature but is found in all 31 archaeal species in the genome set. The sequence is part of various biosynthesis related proteins. Another not yet feature-associated pattern found in all archaeal species is GMDKM, which is part of the archaeal chaperonin thermosome and its homologues in the eukaryotic cytosol (CCT) [14]. Presumably, these patterns form structurally or functionally important motifs in the respective protein.

In the bacterial Swiss-Prot set, the peptides GMQFD (385 occurrences) and MQFDR (375) lack feature assignments and they are found in the 60 kDa chaperonin. In the eukaryotic Swiss-Prot set, the eight most abundant patterns (all with more than 1300 occurrences) are not associated with any known sequence features. These eight peptides (AMHYT, WWNFG, WIWGG, HICRD, PWGQM/QMSFW/MSFWG and EWYFL) are all found in the known conserved regions *Q*_*o*_, *Q*_*i *_and the two haem binding segments in cytochrome b [15], which is vital in eukaryotes as a component of the respiratory chain bc1 complex in mitochondria [16]. We conclude that the patterns must be structurally or functionally important, since they are heavily over-represented (>1300 versus ≤ 2).

Furthermore, among the eukaryotic Swiss-Prot POPs, four sets of overlapping peptide patterns, WTTVW/TVWTD, HVWHM/VWHMP/WHMPA, GHPWG/HPWGN and PFMRW/FMRWR/MRWRD, and the single peptide patterns WNIGI and HRAMH, found approximately 430 times each, lack known sequence features except for the last-named, which is annotated with "binding" and "active site". They are all found in ribulose bisphosphate carboxylase (RuBisCO), which catalyzes the first major step in carbon fixation by the Calvin cycle [17]. Another unfeatured set of peptides is VYPWT/YPWTQ (403/377 occurrences), found in various haemoglobin subunits.

In the eukaryotic genome set, we find two highly abundant peptide patterns, which are not parts of any known features-FHWCC (283 occurrences, 29 eukaryotic species) and WCCYV (207 occurrences, 25 species). The corresponding proteins belong to the Wnt signalling pathway, which is a large family of cysteine-rich secreted glycoproteins controlling development in multicellular organisms [18].

#### Integrases and transposases

In the bacterial genome set, the POP peptide NCWDN is found 218 times although expected only twice. This peptide pattern is found only once in Swiss-Prot, but the description line of the proteins in the genome set that harbor this pattern shows that half of them are integrases. Integrases are usually used by viruses (*e.g*. HIV) to integrate genetic material into the host DNA and have been suggested as therapeutic targets [19]. Note, however, that no virus proteins are included in our genome set, and all these hits are in prokaryotic proteins. The remaining NCWDN-containing proteins are transposases, which are involved in the transfer of transposons within a genome. Hence, as the functions of these two protein families are very similar, the NCWDN peptide pattern might be directly involved in the integrating activity.

The two most abundant peptide patterns in the eukaryotic genome set, WWDHF (569 occurrences) and WCMRH (313), are not found in Swiss-Prot at all, and nearly all protein hits (except 6 and 14, respectively) are to a putative protein retrotransposon in *Oryza sativa *(rice). The peptide pattern YCKWH (203) also occurs mainly in this family. Transposable elements are abundant among the POP and ORP (cf. below) categories from the genome data set, but this high abundance usually originates from only one or a few species (see below for examples). Genome projects differ in whether repeats and transposable elements are included in the main release of protein predictions of a genome, so there is no systematic way to exclude these. Interestingly, the high copy numbers of transposable elements are believed to be important in rapid speciation [20]. Hence, they might be of great evolutionary importance but will distort the distribution of native peptide patterns in studies such as this.

The most frequent peptide pattern in bacterial ORPs from the genomic set, HYNWH (216 occurrences, 9 species), matches 205 copies of transposase from *Bordetella pertussis Tohoma I*. Similarly, 184 of 190 occurrences of the second most frequent peptide pattern, IMTWM, come from transposase copies from only one species, in this case *Mycobacterium ulcerans Agy99*. However, the IMTWM pattern is also found in the transpeptidase region of a penicillin binding protein in the bacterial species *Nostoc sp. PCC 7120 *and *Anabella variabilis ATCC 29413*, which may explain why it does not occur in eukaryotes. The pattern EFWCR (109 occurrences, 8 species) is part of a multicopy transposase protein in *Yersinia pestis *and *Salmonella enterica*.

Also in the genome bacterial URP set we find motifs originating from a retrotransposon protein family, in this case MCVDY (1094), MYCAE (435) and TMYCE (412). All these are significant and primarily found in only one species, rice.

### NEP – negatively selected and structurally/functionally unfavored

NEPs are peptides that we expect to find frequently in view of the composition and size of the sequence database for each kingdom. In fact, however, they are seen only very seldom or not at all. They are retrieved by searching for peptides with at most two observations in original data and with ten or more occurrences in the randomized data. The 100 most frequent in randomized data were selected (see Table 3 for details). NEPs represent negatively-selected peptides that are structurally unfavorable or otherwise avoided by the species in the kingdom. Peptides in this pattern class could be a result of residue bias and all peptides are therefore tested for biological significance (cf. Methods). In our data, all NEPs are significant except for four archaeal NEPs in the Swiss-Prot set. Note, however, that the most expected peptide pattern among NEPs, RGPPW, is only observed 72 times in the randomized set. Hence, even though these peptide patterns are not at all expected, they are not expected in very large numbers in the randomized data set either.

**Table 3:** Categorization of peptide sets

Peptide set	Kingdom	Filter	Top 100 by
POP	A	≥ 10 in A, ≤ 2 in rand. A	Freq. in orig. A
	B	≥ 10 in B, ≤ 2 in rand. B	Freq. in orig. B
	E	≥ 10 in E, ≤ 2 in rand. E	Freq. in orig. E

NEP	A	≤ 2 in A, ≥ 10 in rand. A	Freq. in rand. A
	B	≤ 2 in B, ≥ 10 in rand. B	Freq. in rand. B
	E	≤ 2 in E, ≥ 10 in rand. E	Freq. in rand. E

ORP	A	≥ 10 in A, ≤ 2 in B+E	Freq. in orig. A
	B	≥ 10 in B, ≤ 2 in A+E	Freq. in orig. B
	E	≥ 10 in E, ≤ 2 in A+B	Freq. in orig. E

URP	A	≤ 2 in A, *≥ *10 in B+E	Freq in orig. B+E
	B	≤ 2 in B, *≥ *10 in A+E	Freq in orig. A+E
	E	≤ 2 in E, *≥ *10 in A+B	Freq in orig. A+B

The peptide sets POP, NEP, ORP and URP (for archaea (A), bacteria (B) and eukaryota (E), respectively) were generated by an initial filtering step selecting peptides occurring at least 10 times in one set and at most twice in another, as detailed in the table, then the 100 highest ranked peptide patterns were extracted according to the parameter settings. orig, original; rand, randomized.

#### Archaeal

Only 6–16% of the archaeal NEPs have known features (Table 2). For peptides with feature associations, all except one (in the Swiss-Prot data set) are of the type "transmembrane". These featured peptide patterns are rich in the hydrophobic residues leucine, isoleucine and valine. Leucine and isoleucine are much more abundant in this peptide pattern class than in the archaeal Swiss-Prot proteins overall (Figure 3). These patterns do not exist in archaea, although archaea have more isoleucine and valine residues than eukaryotes and bacteria. One may suspect an inherent restriction on how leucine and isoleucine containing proteins are able to fold into working entities in archaea. However, when archaeal NEPs in the genome data set are examined, no extreme differences in amino acid residue contents are observed. It is possible that the much smaller sample space (shorter total length, see Figure 1) of archaeal sequences in comparison to eukaryotes and bacteria masks some part of the informational pattern. Hence, caution is needed in drawing conclusions about archaeal NEPs, as NEPs are very sensitive to the size of the data set during the data filtration.

#### Bacterial

A common characteristic of NEPs in bacteria is that several patterns contain a proline-proline dipeptide (Table 4), which will have considerable effects on the structure of the protein. Further investigation shows that in both the genome and Swiss-Prot data sets, many bacterial proteins contain this dipeptide; the fraction of eukaryotic proteins that contain it is even larger – more than half the proteins contain a proline-proline dipeptide. Hence, contrary to what might be expected considering the NEP data of bacteria, this structure-affecting dipeptide is frequent in both eukaryotes and bacteria. In the genome set, 38% of the peptide patterns have known features and the most frequently-associated feature is disulfide, which also is frequently associated with bacterial URPs in the genome set.

**Table 4:** The number of patterns that have cysteine-cysteine and proline-proline dipeptides in NEP

	Dipeptide	
Dataset	CC	PP

NEP-A-Genome	0	2
NEP-A-Swissprot	0	0
NEP-B-Genome	0	12
NEP-B-Swissprot	0	14
NEP-E-Genome	12	1
NEP-E-Swissprot	1	1

All-A-Genome	4%	35%
All-A-Swissprot	3%	34%
All-B-Genome	4%	38%
All-B-Swissprot	3%	38%
All-E-Genome	18%	57%
All-E-Swissprot	18%	54%

All: all proteins from the kingdom.		

Dipeptides of cysteine and proline are suspected to be structurally unfavored. Several of the peptides in bacterial NEPs do have diprolines but no dicysteines. Eukaryotic NEPs from the genome set have several dicysteines. However, for NEPs, the fraction of proteins having these dipeptides are considerably lower than in there their respective kingdom section of the full data set.

#### Eukaryotic

For the most expected NEP pattern, CSCCC (40 occurrences in randomized data) in Swiss-Prot, one may suspect that several consecutive cysteines are unfavorable. However, considering the difference in relative residue frequency for eukaryotic NEPs in Swiss-Prot compared to the overall distribution (Figure 3), no extremes are observed for any amino acid residues, which makes this cysteine-rich peptide pattern an exception in this peptide class. Disulfide bridges connect polypeptide chains or distant segments within the same chain and are known to depend on the spacing of cysteines in the linear sequence [21]. Apparently, consecutive cysteines are statistically expected but have been negatively selected during evolution. Eukaryotic NEPs in the genome set contain many of the rare cysteine and tryptophan residues (Figure 3). Several of the NEPs in the genome set also have cysteine–cysteine dipeptides (Table 4). One of the most expected NEPs in the genome set (RCDLM, 50 occurrences in randomized data) is found twice among eukaryotes, in an unknown protein from the plant *Arabidopsis thaliana *and in a novel protein from the fish *Takifugu rubripes*. In contrast, it is found ten times in bacteria and one may speculate that the peptide pattern is part of an immunological triggering epitope, as for bacterial stress response proteins [14].

### ORPs and URPs – kingdom-specifically over- and under-represented peptide patterns

ORPs are peptide patterns that are found at least 10 times in one kingdom and at most twice in the union of the other two kingdoms. They are therefore to be considered unique to one kingdom. Analogously, URPs are peptides not found at all or in only low numbers in a kingdom. As the randomized data set is not used in the filtering step it is possible that the retrieved ORPs and URPs are just a result of compositional bias. To investigate this possibility, the patterns were tested for significance (cf. Methods section).

#### Few statistically significant patterns in archaea

In the Swiss-Prot and genome archaeal ORP sets, only 54 and 6 peptide patterns, respectively, passed the filtering step. The small number of archaeal ORPs that passed the filtering step is largely due to the much shorter total sequence length in archaea compared to those in bacteria and eukaryotes (Figure 1). On the other hand, the resulting peptide patterns have passed a harder filtering criterion and might therefore be considered even more specific than those of bacteria and eukaryotes. Three of the six patterns in the genome set were overlapping (EMCCH/MCCHY/CCHYD) and found 18 times each, all in the same protein and in the same species, *Methanospirillum hungatei JF-1*.

Among archaeal Swiss-Prot URPs, no peptide patterns are biologically significant (*p *< 0.05) and all may result from the much smaller size of the archaeal section of Swiss-Prot. In the genome counterpart, seven peptide patterns are significant. There are numerous examples of peptide patterns found more than 10 000 times in eukaryotes or bacteria. All these have the zinc finger feature but only one is significant, the THTGE pattern with 13 209 occurrences. Other significant peptide patterns come from collagen-associated proteins and cadherin-associated proteins [23].

#### Bacterial-specific patterns

Two of the most abundant bacterial ORP patterns, FRCGF (268 occurrences) and FGFRC (245) in Swiss-Prot, have no feature association but occur in the GTP-binding protein lepA family, the function of which is unknown. The peptide patterns DWMEQ (265 occurrences) and YHDVD (235), together with the overlapping patterns GSYHD (200) and YHDVD (235) in the eukaryotic Swiss-Prot URP set, come from the conserved elongation factor G family, responsible for the accuracy of translation in the ribosome and preserved in all kingdoms. This protein has been suggested as a target for antibiotics [24] and therefore these bacterial-specific patterns now found are interesting sites for further investigation. Two other interesting peptides, MGAQM (234 occurrences) and MNPMD (210 occurrences), are parts of the 60 kDa chaperonin, a protein also found in the bacterial POP set. Like other bacterial stress response proteins, this protein family harbors human immune response activating antigens [14], which explains why these peptide patterns are not found in eukaryotes. The bacterial ORPs in the genome set are rich in tryptophan and have more feature associations than those of the Swiss-Prot set (29% versus 7%), the most common of which is "transmembrane" (Table 2). As in the Swiss-Prot data set, the translational machinery is also represented here, although in this case it is FCDWY (140 occurrences, 138 species), which is found in the bacterial form of valyl-tRNA synthetase. This pattern is also the most widespread of the eukaryotic URPs in the genome set.

About half the eukaryotic Swiss-Prot URPs are significant. Two examples of the most abundant peptide patterns in other kingdoms are YAEGY (270) and VMPQT (223), which are parts of serine hydroxymethyltransferase and translation initiation factor IF-2, respectively. Very few of the eukaryotic Swiss-Prot URPs are feature associated. Among the eukaryotic URPs in the genome set, 80 peptide patterns are found in significantly very low numbers and are therefore expected to be missing for biological reasons. One of these patterns is GWMHD (110 occurrences), which is part of the 1,4-alpha-glucan branching enzyme responsible for the branched structure of glycogen. The enzyme is also found in animal cells, but this peptide pattern seems unique to the bacterial form, known to be different from the eukaryotic version [25]. The GWMHD peptide pattern is widespread in the bacterial kingdom and is found in 100 of 303 of the bacterial species in the genome data set. The peptide pattern QWAYA (133 occurrences, 37 bacterial species) is part of the UDP-N-acetylmuramate-L-alanine ligase, a protein involved in the biosynthesis of the peptidoglycan murein, which is an essential part of the bacterial cell wall. The enzymes involved in this process are interesting antibacterial drug targets as they are not found in eukaryotes [26].

#### Eukaryotic-specific patterns

**Figure 4:**
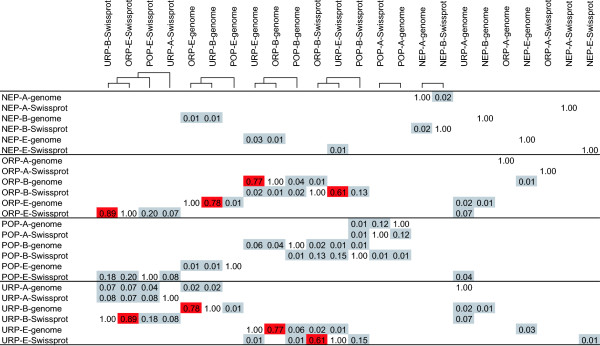
**Overlap of patterns between the peptide pattern subsets**. Numbers give proportions of overlapping patterns. A, B and E indicate the kingdoms of archaea, bacteria and eukaryota, respectively. Clustering is based on single linkage hierarchical clustering and is shown schematically at the top. Peptide subsets with an overlap of 50% or more are red boxed, the remaining are in grey boxes.

In eukaryotic ORPs from the Swiss-Prot data set, the cytochrome b protein family, also found among eukaryotic POPs, contributes to one third of the peptide patterns (approximately 1000 occurrences each). There is a 20% overlap between the peptide patterns in eukaryotic Swiss-Prot ORPs and POPs (Figure 4).

The most common features are "metal" and "zinc finger". Zinc fingers are found in many forms but the peptide patterns in this class are primarily of human origin. Further ORPs originate from homeobox-associated proteins, hemoglobins, and the RuBisCO protein family [17] (cf. POP above). Similarly, for the Swiss-Prot bacterial URPs, we notice cytochrome b and the RuBisCO family. The other features associated with eukaryotic ORPs in the genome set, *e.g*. "disulfide" and "coiled", generally originating from various protein families, indicating that they are independently-occurring common patterns.

In the genome bacterial URP set, about half the URPs are significant. The most widespread peptide pattern among eukaryotic genomes and not found in bacteria is HHCPW (535 occurrences in eukaryotes, 48 species), which is part of the DHHC tetrapeptide sequence motif in a putative zinc finger of the palmitoyltransferase family [27].

The most extreme of eukaryotic ORPs in the genome data set is ECKQC, which is found more than 10 000 times; however, these sequence hits are found in only 34 of the 52 eukaryotic species. WGCFD (379 occurrences, 41 species) is unfeatured and occurs in the dynein protein family, which transports cellular cargo along the microtubules in eukaryotic cells [28]. As these patterns are all biologically significant and not the results of amino acid residue bias, one may suspect that they encode common folds or favorable motifs for eukaryotes.

### Peptide patterns common to POPs, NEPs, ORPs and URPs

The 24 classes of peptide patterns and their respective overlaps are outlined in Figure 4. The largest overlaps are found between ORPs in bacteria and URPs in eukaryotes and vice versa. These evidently have dual properties. ORPs from bacteria have a high (61–77%) overlap with URPs from eukaryotes. The reciprocal case URPs from bacteria and ORPs from eukaryotes are even more similar (78–89%).

For the observed abundance of URPs and ORPs, as opposed to POPs and NEPs, we cannot rule out the possibility that they result only from residue bias due to the filtering step. Therefore we focus on the shared ORP and URP patterns that passed the significance test (see Table 5). In the genome set, known ORP-E/URP-B patterns are primarily zinc fingers, while for ORP-B/URP-E, half occur in transmembrane regions. The latter group is rich in the infrequent tryptophan, but the tryptophan-transmembrane association might be explained by the fact that tryptophan can act as an anchor to the lipid layer at the membrane-water interface and is therefore functionally essential for membrane proteins [10]. In Swiss-Prot, the ORP-E/URP-B patterns are fewer and no clear trend is visible. There are only three Swiss-Prot ORP-B/URP-E peptide patterns, PGCSM, TRMKS and CDKIT. TRMKS is part of the bifunctional protein glmU. This trimeric protein is an attractive target for new antibacterial agents as it is involved in the pathways of peptidoglycan (Gram-positive bacteria) and lipopolysaccharide (Gram-negative bacteria) biosynthesis [29]. The protein has two functionally independent domains, and the TRMKS peptide pattern belongs to the N-terminal pyrophosphorylase domain, which resembles a Rossmann fold. The pattern is part of the strictly-conserved motif of pyrophosphorylases and mutational analysis suggests that the arginine is a catalytic residue [29].

**Table 5:** Common patterns between ORPs and URPs in bacterias and eukaryotes

ORP-E/URP-B				ORP-B/URP-E			
Swiss-Prot		Genome		Swiss-Prot		genome	

WDTAG	NP_BIND	PYVCK	ZN_FING	PGCSM	METAL	WNYWV	TRANSMEM
QGPPG	REGION	KPYTC	ZN_FING	CDKIT	METAL	RCWHY	METAL
EECGK	ZN_FING	YECNQ	ZN_FING	TRMKS	REGION	WAWGH	METAL
FHFIL	METAL	PHECK	ZN_FING			WQGQC	ZN_FING
AFHFI	METAL	KPYNC	ZN_FING			WFPKM	TRANSMEM
NPIIY	TRANSMEM	FECKQ	ZN_FING			MVPMW	TRANSMEM
		NHCGK	ZN_FING			AMWWI	TRANSMEM
		PYQCK	ZN_FING			WGGWW	TRANSMEM
		KPHKC	ZN_FING			WHPEW	ACT_SITE
		PYKCQ	ZN_FING			WGIMH	TRANSMEM
		PYKCT	ZN_FING			IYWHF	DOMAIN
		PCGHN	ZN_FING				
		CMNGG	DISULFID				

RLSCA		AWTWN		GANMQ		PMVWR	GHWYF
GWIIR		ECVWQ		PTDMQ		NFWQM	MTAWH
LRLSC		YWEFQ		ANMQR		YWGCP	WYVVH
ICLFL		YCQEY		GSYHD		GENHW	ANHWM
NYTPA		YHEWT		MIGDP		HGCCH	MWPVH
TLTWI		TMYCE		YHDVD		ACMHC	YWQVY
GHPIS		MIKCY		PYRKV		HNWPG	WIAAW
RNLSH		CYIFM		YPAME		SHIWY	EFWCR
KQRSM		GFHCN		RDVHP		WPMKH	MNAWA
FCAEA		SKFWY		LPHRY		WWIKA	YPCNY
		FCQVR		FHIGG		WMPSW	LCHYW
		CKQDV		TYNFP		FPMDW	IRWQH
		RSKFW		VMFGN		FCDWY	HWAYK
		NTWHR		MIEGP		WHKRP	MGKWL
		CKPPN		NIMEF		IEAHW	FWWNP
		MFGCP		VYKHA		TMWRG	GWLWF
		MWIPK		HGTYP		WMAMN	GMNKW
		PKYCI		KDHHS		FGWQV	PRHYW
		QNVMC				AHDWC	WRNAW
		MCVDY				WDMNF	MAHDW
		MYCEA				IMTWM	WAMTQ
		WWVSM				EHWHT	RHWMI
		RNMCP				TYAMW	

Table shows patterns that are ORPs in eukaryotes and URPs in bacteria and vice versa. The upper part shows pattern with associated features and the lower part lists those without feature association. Only peptide patterns where residue bias can be excluded are shown (*p *≤ 0.05).

Most patterns are not associated with any known feature, but are probably part of an important biological entity unique to the respective kingdom, which has not been elucidated so far. Among the unfeatured ORP-E/URP-B, the four patterns LRLSC, RLSCA, GHPIS and RNLSH are described as maturase K-associated in the ORP-E section. The remaining peptide patterns, not described earlier, are all cytochrome b associated. In the genome data set many of the peptide patterns are observed in only a few organisms, and the common theme of these seems to be the retrotransposase family discussed earlier. The four peptides ECVWQ, CKQDV, PKYCI and SKFWY are found in 20 or more of the 52 eukaryotic genomes, but have no common descriptions; however, the many occurrences of the last two are due to hits in multiple proteins in only one organism, *Trichomonas vaginalis *and rice, respectively.

### Effects of databank growth

All numbers in this study are dependent on how many protein sequences have been discovered to date (2007). Some insight into the effect of time can be offered, as we have a similar data set (unpublished) from 2003. Swiss-Prot has increased by 33% in length and 40% in number of proteins, but the fractions of shared pentapeptide patterns (Figure 2) are still similar. Hence it seems that the growth of Swiss-Prot is fairly homogeneous. The genome set, however, has increased by 200% in length and 188% in number of proteins. It now includes 386 species compared to 137 in 2003. Interestingly, though, the total sequence fraction of bacteria is larger in 2007 than 2003. This stands even though we included only one strain per species in the current study, and completely-sequenced organisms with multiple strains are mostly bacterial. Another notable difference in the genome set is that the fraction of patterns unique to eukaryotes has decreased from 5% to 1% and that patterns that are found in all kingdoms have increased from 62% to 75%. Furthermore, the number of unobserved pentapeptide patterns has decreased by less than one percentage point, while the non-existent hexapeptide patterns have decreased by approximately 7 percentage points for Swiss-Prot and about 15 percentage points for the genome set. Hence, databank growth does affect oligopeptide patterns of length six and longer, but it seems that we have already reached saturation of the available patterns for oligopeptides of length five.

### Outlook

Methodology and ideas from this study may be important in further studies. An interesting application would be to construct a predictor for protein-coding sequences that is different from ab initio algorithms such as Genscan [30]. One such effort has already provided complementary information on this subject [31]. However, the training data in that study were limited to structural motifs of 471 proteins and Pfam alignments, the latter only accounting for 38% of the Swiss-Prot sequences. The informational content of short oligopeptides such as those in our study might possibly be used to distinguish features in truly-expressed exons from those in translated introns and open reading frames, that have been frame-shifted.

## Conclusion

Although there are no obvious differences in amino acid residue preferences between the genome and Swiss-Prot sets, we see marked differences in pentapeptide characteristics. Almost all pentapeptide patterns exist, but there are sets of over- and under-represented patterns that are extreme in frequencies, even if compositional bias is considered. The abundances of many of the highly represented peptide patterns in this study can be explained on the basis of the protein families from which they originate. Notably, only a few protein families give rise to most of the over- and under-represented peptide patterns between kingdoms. These are mainly in three categories: (i) proteins widespread in a kingdom, such as respiratory chain-associated cytochromes and proteins associated with the translation machinery; (ii) patterns with unassigned functions, of special interest for understanding structural and functional mechanisms of proteins; and (iii) multicopy proteins such as retrotransposons, which usually carry a species-unique peptide pattern. Categories (i) and (ii) are found in both Swiss-Prot and the genome set while category (iii) is found only in the genome set. In our study we used only one set for each species, but for many of the completely-sequenced species there are multiple releases for several strains, suggesting that if included, category (iii) protein families will give rise to even more extreme numbers of occurrences. As sequence patterns are fundamental in many bioinformatics algorithms, this raises questions about the need to correct for over-represented peptide patterns such as those found in this study.

## Methods

### Preparation of datasets

The UniprotKB/Swiss-Prot database (release 51.5, January 2007), which consists of 255 000 sequences, was downloaded from EBI [7,8]. All proteins of viral origin (8000 proteins) were removed from the original release to make the Swiss-Prot data set comparable to the genome data set. The genome set was assembled from complete genomes downloaded from GenBank [32], TIGR [33] and EnsEMBL [34,35] (January 2007). For genomes with multiple strains, only the strain with the largest number of proteins was included, resulting in a set of 386 completely-sequenced organisms representing 31 archaeal, 303 bacterial and 52 eukaryotic species, with a total of 2 million protein sequences. Details of the genomes included in this data set are given in Additional file 5.

For statistical comparisons, reference sets were generated from the genome and Swiss-Prot data sets by randomizing the original sequence data, on a per protein basis. Given a set of original protein sequences Ω = *o*_1_, *o*_2_, ..., *o*_*j*_, ..., *o*_*N *_where the protein sequence *o*_*j *_has the letters *o*_*j*1_*o*_*j*2 _...*o*_*jk*_...ojlen(oj)
 MathType@MTEF@5@5@+=feaafiart1ev1aaatCvAUfKttLearuWrP9MDH5MBPbIqV92AaeXatLxBI9gBaebbnrfifHhDYfgasaacH8akY=wiFfYdH8Gipec8Eeeu0xXdbba9frFj0=OqFfea0dXdd9vqai=hGuQ8kuc9pgc9s8qqaq=dirpe0xb9q8qiLsFr0=vr0=vr0dc8meaabaqaciaacaGaaeqabaqabeGadaaakeaacqWGVbWBdaWgaaWcbaGaemOAaOMaemiBaWMaemyzauMaemOBa4MaeiikaGIaem4Ba82aaSbaaWqaaiabdQgaQbqabaWccqGGPaqkaeqaaaaa@3863@ (using the amino acid residue alphabet, *A*). From Ω we create a set of permutated protein sequences Π = *p*_1_, *p*_2_, ..., *p*_*j*_, ..., *p*_*N *_where *p*_*j *_contains all the letters from *o*_*j *_but in arbitrary order, *p*_*j *_= *randomize*(*o*_*j*_). The function *randomize *is defined as,

randomize(*o*_*j*_)

   randObj = instantiate new randomization object with new seed.

   permutatedSequence = ""

   while *len*(*o*_*j*_) > 0 :

      k = randObj.randrange(1, len(*o*_*j*_))

      permutatedSequence += *o*_*jk*_

      from *o*_*j *_remove *o*_*jk*_

   return permutatedSequence

That is, each randomized sequence was created by adding residues one at a time from a random position in the original sequence. A new residue was taken at each iteration until the original sequence was consumed.

This resulted in a randomized sequence of the same length and with the same amino acid residue composition as the original.

### Preparing peptide pattern sets

The abundances of oligopeptides of lengths four, five and six in the original and randomized sets were retrieved by sliding a window of length *d *= 4,5,6 over all the protein sequences (Table 1). Words *ω*_*i *_of length *d *can be constructed given the alphabet *A *of length *L*. The dictionary *D*_*d *_holds all combinations of the words *ω*_*i *_where *i *= 1, 2, .., *L*^*d*^. We define a function for the occurrences of a word *ω*_*i *_in the set of sequences Ω (and analogously ∏) as

occurrences(ωi,Ω)=∑j=1N∑k=1len(oj)−d+1π(ojk,ωi)
 MathType@MTEF@5@5@+=feaafiart1ev1aaatCvAUfKttLearuWrP9MDH5MBPbIqV92AaeXatLxBI9gBaebbnrfifHhDYfgasaacH8akY=wiFfYdH8Gipec8Eeeu0xXdbba9frFj0=OqFfea0dXdd9vqai=hGuQ8kuc9pgc9s8qqaq=dirpe0xb9q8qiLsFr0=vr0=vr0dc8meaabaqaciaacaGaaeqabaqabeGadaaakeaacqWGVbWBcqWGJbWycqWGJbWycqWG1bqDcqWGYbGCcqWGYbGCcqWGLbqzcqWGUbGBcqWGJbWycqWGLbqzcqWGZbWCcqGGOaakiiGacqWFjpWDdaWgaaWcbaGaemyAaKgabeaakiabcYcaSiabfM6axjabcMcaPiabg2da9maaqadabaWaaabmaeaacqWFapaCcqGGOaakcqWGVbWBdaWgaaWcbaGaemOAaOMaem4AaSgabeaakiabcYcaSiab=L8a3naaBaaaleaacqWGPbqAaeqaaOGaeiykaKcaleaacqWGRbWAcqGH9aqpcqaIXaqmaeaacqWGSbaBcqWGLbqzcqWGUbGBcqGGOaakcqWGVbWBdaWgaaadbaGaemOAaOgabeaaliabcMcaPiabgkHiTiabdsgaKjabgUcaRiabigdaXaqdcqGHris5aaWcbaGaemOAaOMaeyypa0JaeGymaedabaGaemOta4eaniabggHiLdaaaa@68F6@

where *π *(*o*_*jk*_, *ω*_*i*_) is an indicator function.

π(ojk,ωi)={1,ojkojk+1...ojk+d−1=ωi0,otherwise
 MathType@MTEF@5@5@+=feaafiart1ev1aaatCvAUfKttLearuWrP9MDH5MBPbIqV92AaeXatLxBI9gBaebbnrfifHhDYfgasaacH8akY=wiFfYdH8Gipec8Eeeu0xXdbba9frFj0=OqFfea0dXdd9vqai=hGuQ8kuc9pgc9s8qqaq=dirpe0xb9q8qiLsFr0=vr0=vr0dc8meaabaqaciaacaGaaeqabaqabeGadaaakeaaiiGacqWFapaCcqGGOaakcqWGVbWBdaWgaaWcbaGaemOAaOMaem4AaSgabeaakiabcYcaSiab=L8a3naaBaaaleaacqWGPbqAaeqaaOGaeiykaKIaeyypa0ZaaiqabeaafaqaaeGacaaabaGaeGymaeJaeiilaWcabaGaem4Ba82aaSbaaSqaaiabdQgaQjabdUgaRbqabaGccqWGVbWBdaWgaaWcbaGaemOAaOMaem4AaSMaey4kaSIaeGymaedabeaakiabc6caUiabc6caUiabc6caUiabd+gaVnaaBaaaleaacqWGQbGAcqWGRbWAcqGHRaWkcqWGKbazcqGHsislcqaIXaqmaeqaaOGaeyypa0Jae8xYdC3aaSbaaSqaaiabdMgaPbqabaaakeaacqaIWaamcqGGSaalaeaacqqGVbWBcqqG0baDcqqGObaAcqqGLbqzcqqGYbGCcqqG3bWDcqqGPbqAcqqGZbWCcqqGLbqzaaaacaGL7baaaaa@64ED@

For further analysis only the pentapeptide sets were used. The choice of length five was a compromise between complexity of the sequence patterns and the informational content of the possible set of words of length *d *(*e.g*. setting *d *= 6, we observe only 39–91% of the possible words, Table 1).

The abundances of pentapeptides in the original and randomized sets were retrieved using a Linux Cluster of Beowulf design of 32 nodes (1800+ AMD CPU, 512 MB RAM per node) and a 64 bit Linux system with 8 GB of RAM. The sets of POPs, ORPs, URPs and NEPs were generated by filtering the data according to the rules in Table 3 and then selecting the 100 top-ranked peptides. Within a set, the peptide sequences were clustered by scoring ungapped pairwise alignments using an identity matrix. Multiple sequence alignment was made by grouping them by single-linkage hierarchical clustering and using a cut-off score value of three or more.

### Significance of peptide patterns

A peptide pattern that has passed the filtering and ranking steps might not necessarily indicate biological importance but rather compositional bias. To determine whether this is the case, the resulting peptides were tested for significance. For the word *ω*_*i *_with the sequence *a*_1_*a*_2_...*a*_*d *_we build a set *W*_*i *_= {*ω*_*i*1_, *ω*_*i*1_, ..., *ω*_*il*_, ..., *ω*_id*_} of words with all unique combination of the letters *a*_1_, *a*_2_,..., *a*_*d *_where d* is the number of unique combinations (i.e. d* = *d*! if *a*_1 _≠ *a*_2 _≠ ... ≠ *a*_*d*_). Then we estimate the expectation value of occurrences, ω¯i
 MathType@MTEF@5@5@+=feaafiart1ev1aaatCvAUfKttLearuWrP9MDH5MBPbIqV92AaeXatLxBI9gBaebbnrfifHhDYfgasaacH8akY=wiFfYdH8Gipec8Eeeu0xXdbba9frFj0=OqFfea0dXdd9vqai=hGuQ8kuc9pgc9s8qqaq=dirpe0xb9q8qiLsFr0=vr0=vr0dc8meaabaqaciaacaGaaeqabaqabeGadaaakeaaiiGacuWFjpWDgaqeamaaBaaaleaacqWGPbqAaeqaaaaa@301F@, in ∏ by

ω¯i,Π=∑l=1d∗occurrences(ωil,Π)d∗
 MathType@MTEF@5@5@+=feaafiart1ev1aaatCvAUfKttLearuWrP9MDH5MBPbIqV92AaeXatLxBI9gBaebbnrfifHhDYfgasaacH8akY=wiFfYdH8Gipec8Eeeu0xXdbba9frFj0=OqFfea0dXdd9vqai=hGuQ8kuc9pgc9s8qqaq=dirpe0xb9q8qiLsFr0=vr0=vr0dc8meaabaqaciaacaGaaeqabaqabeGadaaakeaaiiGacuWFjpWDgaqeamaaBaaaleaacqWGPbqAcqGGSaalcqqHGoauaeqaaOGaeyypa0ZaaSaaaeaadaaeWaqaaiabd+gaVjabdogaJjabdogaJjabdwha1jabdkhaYjabdkhaYjabdwgaLjabd6gaUjabdogaJjabdwgaLjabdohaZjabcIcaOiab=L8a3naaBaaaleaacqWGPbqAcqWGSbaBaeqaaOGaeiilaWIaeuiOdaLaeiykaKcaleaacqWGSbaBcqGH9aqpcqaIXaqmaeaacqWGKbazdaahaaadbeqaaiabgEHiQaaaa0GaeyyeIuoaaOqaaiabdsgaKnaaCaaaleqabaGaey4fIOcaaaaaaaa@55C0@

and the estimated standard deviation

sωi,Π=∑l=1d∗(occurrences(ωil,Π)−ω¯i,Π)2d∗
 MathType@MTEF@5@5@+=feaafiart1ev1aaatCvAUfKttLearuWrP9MDH5MBPbIqV92AaeXatLxBI9gBaebbnrfifHhDYfgasaacH8akY=wiFfYdH8Gipec8Eeeu0xXdbba9frFj0=OqFfea0dXdd9vqai=hGuQ8kuc9pgc9s8qqaq=dirpe0xb9q8qiLsFr0=vr0=vr0dc8meaabaqaciaacaGaaeqabaqabeGadaaakeaacqWGZbWCdaWgaaWcbaacciGae8xYdC3aaSbaaWqaaiabdMgaPjabcYcaSiabfc6aqbqabaaaleqaaOGaeyypa0ZaaSaaaeaadaaeWaqaaiabcIcaOiabd+gaVjabdogaJjabdogaJjabdwha1jabdkhaYjabdkhaYjabdwgaLjabd6gaUjabdogaJjabdwgaLjabdohaZjabcIcaOiab=L8a3naaBaaaleaacqWGPbqAcqWGSbaBaeqaaOGaeiilaWIaeuiOdaLaeiykaKIaeyOeI0Iaf8xYdCNbaebadaWgaaWcbaGaemyAaKMaeiilaWIaeuiOdafabeaakiabcMcaPmaaCaaaleqabaGaeGOmaidaaaqaaiabdYgaSjabg2da9iabigdaXaqaaiabdsgaKnaaCaaameqabaGaey4fIOcaaaqdcqGHris5aaGcbaGaemizaq2aaWbaaSqabeaacqGHxiIkaaaaaaaa@60D1@

A null hypothesis was stated that *occurrences*(*ω*_*i*_, Ω) is N(ω¯i,Π,sωi,Π)
 MathType@MTEF@5@5@+=feaafiart1ev1aaatCvAUfKttLearuWrP9MDH5MBPbIqV92AaeXatLxBI9gBaebbnrfifHhDYfgasaacH8akY=wiFfYdH8Gipec8Eeeu0xXdbba9frFj0=OqFfea0dXdd9vqai=hGuQ8kuc9pgc9s8qqaq=dirpe0xb9q8qiLsFr0=vr0=vr0dc8meaabaqaciaacaGaaeqabaqabeGadaaakeaacqWGobGtcqGGOaakiiGacuWFjpWDgaqeamaaBaaaleaacqWGPbqAcqGGSaalcqqHGoauaeqaaOGaeiilaWIaem4Cam3aaSbaaSqaaiab=L8a3naaBaaameaacqWGPbqAcqGGSaalcqqHGoauaeqaaaWcbeaakiabcMcaPaaa@3D9C@. A *p*-value for *x *observations of *ω*_*i *_was calculated and the null hypothesis was rejected for all *ω*_*i *_with a *p*-value less than or equal to 0.05. Those that not belonged to the null hypothesis were considered to be biologically significant. That is all POPs and ORPs that satisfy

P(*x ≥ occurences*(*ω*_*i*_, Ω)|N(ω¯i,Π,sωi,Π)
 MathType@MTEF@5@5@+=feaafiart1ev1aaatCvAUfKttLearuWrP9MDH5MBPbIqV92AaeXatLxBI9gBaebbnrfifHhDYfgasaacH8akY=wiFfYdH8Gipec8Eeeu0xXdbba9frFj0=OqFfea0dXdd9vqai=hGuQ8kuc9pgc9s8qqaq=dirpe0xb9q8qiLsFr0=vr0=vr0dc8meaabaqaciaacaGaaeqabaqabeGadaaakeaacqWGobGtcqGGOaakiiGacuWFjpWDgaqeamaaBaaaleaacqWGPbqAcqGGSaalcqqHGoauaeqaaOGaeiilaWIaem4Cam3aaSbaaSqaaiab=L8a3naaBaaameaacqWGPbqAcqGGSaalcqqHGoauaeqaaaWcbeaakiabcMcaPaaa@3D9C@) ≤ 0.05

and all NEPs and URPs that satisfy

P(*x *≤ *occurences*(*ω*_*i*_, Ω)|N(ω¯i,Π,sωi,Π)
 MathType@MTEF@5@5@+=feaafiart1ev1aaatCvAUfKttLearuWrP9MDH5MBPbIqV92AaeXatLxBI9gBaebbnrfifHhDYfgasaacH8akY=wiFfYdH8Gipec8Eeeu0xXdbba9frFj0=OqFfea0dXdd9vqai=hGuQ8kuc9pgc9s8qqaq=dirpe0xb9q8qiLsFr0=vr0=vr0dc8meaabaqaciaacaGaaeqabaqabeGadaaakeaacqWGobGtcqGGOaakiiGacuWFjpWDgaqeamaaBaaaleaacqWGPbqAcqGGSaalcqqHGoauaeqaaOGaeiilaWIaem4Cam3aaSbaaSqaaiab=L8a3naaBaaameaacqWGPbqAcqGGSaalcqqHGoauaeqaaaWcbeaakiabcMcaPaaa@3D9C@) ≤ 0.05

Note that the estimation of the null distribution of a pentapeptide is based on at most 120 samples, and for patterns with less than five different residues this number is even lower. For homopeptides (which are found only in a few cases in our peptide categories), no permutations are possible, hence a null distribution will be based on only one sample. Therefore, the *p*-value should be considered more as a guide for excluding statistically expected patterns than an accurate calculation of a probability.

### Extracting descriptive feature of patterns

To determine whether a peptide is part of any novel or known feature, a scan against all Swiss-Prot entries (release 51.5) was performed. Every hit in the FT field was recorded and those features that covered at least one fifth of the sequence hits are listed in Additional files 3 and 4. Ambiguous features such as "chain", "domain", "topological domain" and "region" were discarded in further analysis. A python script to retrieve fasta headers was used to retrieve information about proteins in which a certain peptide pattern was observed.

## Abbreviations

A archaea

B bacteria

E eukaryota

NEP negatively selected peptides

ORP over-represented peptides, in a kingdom

POP positively selected peptides

RuBisCO ribulose bisphosphate carboxylase

URP under-represented peptides, in a kingdom

## Authors' contributions

Anders Bresell performed the data collection, computer programming and analysis. Bengt Persson initiated, planned and supervised the study. Both authors contributed ideas and wrote the manuscript.

## Supplementary Material

Additional file 1 Relative amino acid residue composition in Swiss-Prot and genome set. Detailed analysis of difference between kingdoms and data sets. The data in Figure 3 is derived from these background values. Click here for file

Additional file 2 Selected peptide patterns. The table shows the peptide patterns that have been discussed in the article, but also other peptide patterns of general interest are included. The patterns for which no conclusions could be made by analyzing the observed occurrences, protein family association, kingdom coverage or literature, are not listed here – but are found in Additional files 3 and 4. Click here for file

Additional file 3 Top 100 peptides of each category in Swiss-Prot data set. Data for peptide classes of the Swiss-Prot data set. The table is provided as PDF. Each peptide category (POP, NEP, ORP and ORP) is shown on a separate page. Each peptide category has three columns for the kingdoms archaea (A), bacteria (B) and eukaryota (E). Each kingdom has five columns; 1. Aligned peptide patterns. 2. For POP and ORP, the number of occurrences in original data for that kingdom; for NEP, the number of occurrences in randomized data for that kingdom; for URP, the number of occurrences in original data for the other two kingdoms. Most extreme values are color-coded with green background. 3. The *p*-value for biological significance (see Methods section for details). Significant values are color-coded with orange background (*p *≤ 0.05). 4. The number of individual sequence region hits in Swiss-Prot release 51.5. 5. Swiss-Prot sequence features (FT field) and the fraction of sequence hits in column 4 that are mapped to this feature. Only features of at least 20% coverage are reported. Features with more than 50% coverage are color coded with background in magenta. Click here for file

Additional file 4 Top 100 peptides of each category in genome data set. Data table for peptide classes of the genome data set. As for Additional file 3, with additional 1–3 columns introduced between columns 4 and 5, showing the number of species in which the peptide pattern was found. Click here for file

Additional file 5 Details of genome data set. The table shows detailed information on sources of data included in the genome set. Columns are separated with a tab-character with one source file on each row. If a species has multiple entries (*e.g*. one file for each chromosome), then the files are concatenated on the basis of their NCBI taxonomic id. Columns are: 1. NCBI Taxonomy lineage. 2. NCBI Taxonomy id. 3. NCBI Taxonomy scientific name. 4. Species name on source server. 5. Filepath on source server. 6. Source server (FTP). 7. Login directory. 8. Size in bytes. 9. Last modification date on source server. Click here for file

## References

[B1] Pe'er I, Felder CE, Man O, Silman I, Sussman JL, S BJ (2004). Proteomics Singatures: Amino Acid and oligopeptide Composistions Differentiate Among Phyla. Proteins.

[B2] Tekaia F, Yeramian E (2006). Evolution of proteomes: fundamental signatures and global trends in amino acid compositions. BMC Genomics.

[B3] Otaki JM, Ienaka S, Gotoh T, Yamamoto H (2005). Availability of short amino acid sequences in proteins. Protein Sci.

[B4] Figureau A, Soto MA, Toha J (2003). A pentapeptide-based method for protein secondary structure prediction. Protein Eng.

[B5] Yang AS, yong Wang L (2003). Local structure prediction with local structure-based sequence profiles. Bioinformatics.

[B6] Sigrist CJA, Cerutti L, Hulo N, Gattiker A, Falquet L, Pagni M, Bairoch A, Bucher P (2002). PROSITE: a documented database using patterns and profiles as motif descriptors. Brief Bioinform.

[B7] Bairoch A, Boeckmann B (1992). The SWISS-PROT protein sequence data bank. Nucleic Acids Res.

[B8] Boeckmann B, Bairoch A, Apweiler R, Blatter M, Estreicher A, Gasteiger E, Martin M, Michoud K, O'Donovan C, Phan I, Pilbout S, Schneider M (2003). The SWISS-PROT protein knowledgebase and its supplement TrEMBL in 2003. Nucleic Acids Res.

[B9] Barford D (2004). The role of cysteine residues as redox-sensitive regulatory switches. Curr Opin Struct Biol.

[B10] Killian JA, von Heijne G (2000). How proteins adapt to a membrane-water interface. Trends Biochem Sci.

[B11] Klein-Seetharaman J, Oikawa M, Grimshaw SB, Wirmer J, Duchardt E, Ueda T, Imoto T, Smith LJ, Dobson CM, Schwalbe H (2002). Long-range interactions within a nonnative protein. Science.

[B12] Chan DI, Prenner EJ, Vogel HJ (2006). Tryptophan- and arginine-rich antimicrobial peptides: structures and mechanisms of action. Biochim Biophys Acta.

[B13] Martin JL (1995). Thioredoxin-a fold for all reasons. Structure.

[B14] Klumpp M, Baumeister W (1998). The thermosome: archetype of group II chaperonins. FEBS Lett.

[B15] Howell N (1989). Evolutionary conservation of protein regions in the protonmotive cytochrome b and their possible roles in redox catalysis. J Mol Evol.

[B16] Zhang Z, Huang L, Shulmeister VM, Chi YI, Kim KK, Hung LW, Crofts AR, Berry EA, Kim SH (1998). Electron transfer by domain movement in cytochrome bc1. Nature.

[B17] Spreitzer RJ, Salvucci ME (2002). Rubisco: structure, regulatory interactions, and possibilities for a better enzyme. Annu Rev Plant Biol.

[B18] Miller JR (2002). The Wnts. Genome Biol.

[B19] Thomas M, Brady L (1997). HIV integrase: a target for AIDS therapeutics. Trends Biotechnol.

[B20] Noor MAF, Chang AS (2006). Evolutionary genetics: jumping into a new species. Curr Biol.

[B21] Tsai CH, Chen BJ, Chan CH, Liu HL, Kao CY (2005). Improving disulfide connectivity prediction with sequential distance between oxidized cysteines. Bioinformatics.

[B22] Dennis GJ, Sherman BT, Hosack DA, Yang J, Gao W, Lane HC, Lempicki RA (2003). DAVID: Database for  Annotation, Visualization, and Integrated Discovery. Genome Biol.

[B23] Halbleib JM, Nelson WJ (2006). Cadherins in development: cell adhesion, sorting, and tissue morphogenesis. Genes Dev.

[B24] Andersen GR, Nissen P, Nyborg J (2003). Elongation factors in protein biosynthesis. Trends Biochem Sci.

[B25] Guan H, Kuriki T, Sivak M, Preiss J (1995). Maize branching enzyme catalyzes synthesis of glycogen-like polysaccharide in glgB-deficient Escherichia coli. Proc Natl Acad Sci USA.

[B26] Spraggon G, Schwarzenbacher R, Kreusch A, Lee CC, Abdubek P, Ambing E, Biorac T, Brinen LS, Canaves JM, Cambell J, Chiu HJ, Dai X, Deacon AM, DiDonato M, Elsliger MA, Eshagi S, Floyd R, Godzik A, Grittini C, Grzechnik SK, Hampton E, Jaroszewski L, Karlak C, Klock HE, Koesema E, Kovarik JS, Kuhn P, Levin I, McMullan D, McPhillips TM, Miller MD, Morse A, Moy K, Ouyang J, Page R, Quijano K, Robb A, Stevens RC, van den Bedem H, Velasquez J, Vincent J, von Delft F, Wang X, West B, Wolf G, Xu Q, Hodgson KO, Wooley J, Lesley SA, Wilson IA (2004). Crystal structure of an Udp-n-acetylmuramate-alanine ligase MurC (TM0231) from Thermotoga maritima at 2.3 A resolution. Proteins.

[B27] Mitchell DA, Vasudevan A, Linder ME, Deschenes RJ (2006). Protein palmitoylation by a family of DHHC protein S-acyltransferases.. 2006 Jun;47(6):1118-27. Epub 2006 Apr 1  PMID: 16582420. J Lipid Res.

[B28] Serohijos AWR, Chen Y, Ding F, Elston TC, Dokholyan NV (2006). A structural model reveals energy transduction in dynein. Proc Natl Acad Sci USA.

[B29] Brown K, Pompeo F, Dixon S, Mengin-Lecreulx D, Cambillau C, Bourne Y (1999). Crystal structure of the bifunctional N-acetylglucosamine 1-phosphate uridyltransferase from Escherichia coli: a paradigm for the related pyrophosphorylase superfamily. EMBO J.

[B30] Burge C, Karlin S (1997). Prediction of complete gene structures in human genomic DNA. J Mol Biol.

[B31] Thayer EC, Bystroff C, Baker D (2000). Detection of protein coding sequences using a mixture model for local protein amino acid sequence. J Comput Biol.

[B32] Benson D, Karsch-Mizrachi I, Lipman D, Ostell J, Wheeler D (2004). GenBank. Nucleic Acids Res.

[B33] The Institute for Genomic Research. http://www.tigr.org.

[B34] Hubbard T, Barker D, Birney E, Cameron G, Chen Y, Clark L, Cox T, Cuff J, Curwen V, Down T, Durbin R, Eyras E, Gilbert J, Hammond M, Huminiecki L, Kasprzyk A, Lehvaslaiho H, Lijnzaad P, Melsopp C, Mongin E, Pettett R, Pocock M, Potter S, Rust A, Schmidt E, Searle S, Slater G, Smith J, Spooner W, Stabenau A, Stalker J, Stupka E, Ureta-Vidal A, Vastrik I, Clamp M (2002). The Ensembl genome database project. Nucleic Acids Res.

[B35] Hubbard T, Andrews D, Caccamo M, Cameron G, Chen Y, Clamp M, Clarke L, Coates G, Cox T, Cunningham F, Curwen V, Cutts T, Down T, Durbin R, Fernandez-Suarez X, Gilbert J, Hammond M, Herrero J, Hotz H, Howe K, Iyer V, Jekosch K, Kahari A, Kasprzyk A, Keefe D, Keenan S, Kokocinsci F, London D, Longden I, McVicker G, Melsopp C, Meidl P, Potter S, Proctor G, Rae M, Rios D, Schuster M, Searle S, Severin J, Slater G, Smedley D, Smith J, Spooner W, Stabenau A, Stalker J, Storey R, Trevanion S, Ureta-Vidal A, Vogel J, White S, Woodwark C, Birney E (2004). Ensembl 2005. Nucleic Acids Res.

