# Supplementary material for: Characterization of oligopeptide patterns in large protein sets
Source: BMC Genomics. 2007 Oct 1;8:346. doi: 10.1186/1471-2164-8-346 (PMC2231379; doi:10.1186/1471-2164-8-346)

Additional file 1 – Relative amino acid residue composition in Swiss-Prot and genome set

The figure shows the overall relative amino acid compositions for each kingdom in the two data sets, ordered by their average frequencies in the respective data set. Comparing differences among the kingdoms, we see that alanine (A) is more frequent in bacteria than in the other kingdoms. Similarly, serine (S) and cysteine (C) are preferred in eukaryotes in both data sets. In archaea, glutamate (E) and isoleucine (I) are more common and glutamine (Q) less common than in the other kingdoms. Also some differences are also seen between the data sets, for instance lysine (K) is more frequent in archaea than in other kingdoms in the Swiss-Prot set, while in the genome set the lysine frequency is lower in bacteria. Our data do not conflict with a habitat-amino acid usage correlation study [2] and are consistent with kingdom classification via principal component analysis [1].

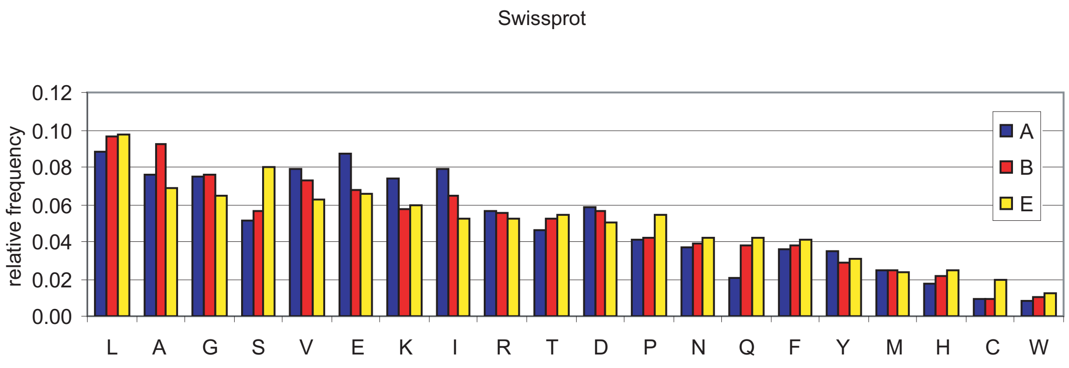

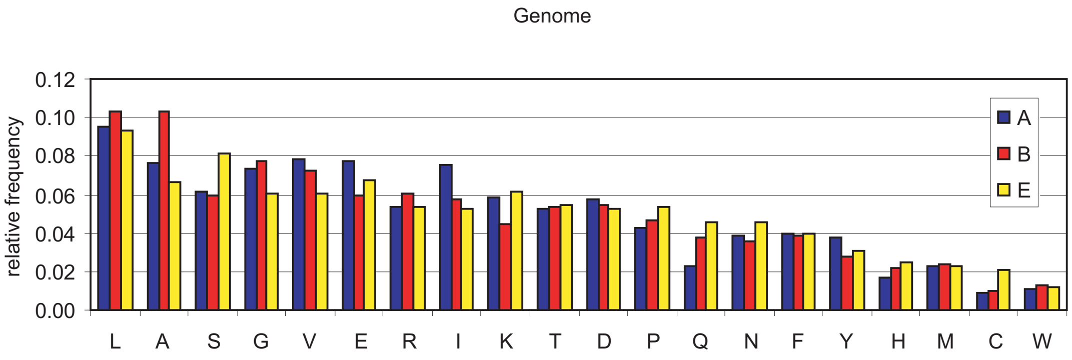

Supplement: Additional file 1 — Relative amino acid residue composition in Swiss-Prot and genome set. Detailed analysis of difference between kingdoms and data sets. The data in Figure 3 is derived from these background values. [file 1471-2164-8-346-S1.pdf]
