# Supplementary material for: Characterization of oligopeptide patterns in large protein sets
Source: BMC Genomics. 2007 Oct 1;8:346. doi: 10.1186/1471-2164-8-346 (PMC2231379; doi:10.1186/1471-2164-8-346)
Supplement: Additional file 2 — Selected peptide patterns. The table shows the peptide patterns that have been discussed in the article, but also other peptide patterns of general interest are included. The patterns for which no conclusions could be made by analyzing the observed occurrences, protein family association, kingdom coverage or literature, are not listed here – but are found in Additional files 3 and 4. [file 1471-2164-8-346-S2.pdf]

Additional file 2 - Selected peptide patterns

Category	Kingdom	Data set	Pattern	Numer of occurrences	Number of species	Feature	Protein family	Note
POP	A	sp	GSGKT	117		No feature	Protein with kinase, ATP-binding or helicase properties	
POP	A	g	WDFGD	303	12	Domain and topo domain	Found in many different proteins	
POP	A	g	CPKCG	273	30	No feature	Found in many different proteins	
POP	A	g	CPVCG	258	31	No feature		Found in all archaeal species
POP	A	g	GMDKM	59	31	No feature	chaperonin thermosome	Found in all archaeal species
POP	B	sp	QFHPE	644		Active site		
POP	B	sp	MIFQH/IFQHF/FQHFN	179/181/201		Domain	Methionine import ATP-binding protein metN	
POP	B	sp	AKCYG/KCYGG/CYGGD	345/300/302		No feature	GTP-binding protein lepA	
POP	B	sp	TVWRQ/VWRQA	335/349		No feature	Elongation factor G	
POP	B	sp	GMQFD/MQFDR	385/375		No feature	60 kDa chaperonin	
POP	B	g	WCGPC	599	275	Disulfide and active site	Thioredoxins	Found in almost all bacterial species
POP	B	g	CTTNC	396	273	Active site	Glyceraldehyde-3-phosphate dehydrogenase	Found in almost all bacterial species, second cystein catalytic resiude [ref ST1]
POP	B	g	NCWDN	218	41	No feature	Integrase or tranposase	Found only once in Swissprot
POP	E	sp	AMHYT, WWNFG, WIWGG, HICRD, PWGQM, QMSFW/MSFWG and EWYFL	>1300			Cytochrome b protein	Part of the known conserved regions Qo, Qi and the two heme binding segments [ref ST2]
POP	E	sp	IRYMH/RYMHA/YMHAN	~500		metal and hemegroup	Cytochrome b protein	
POP	E	sp	WNIGI	~500			Cytochrome b protein	
POP	E	sp	QCLFW	~500			Cytochrome b protein	
POP	E	sp	VWFQN/WFQNR, KIWFQ/IWFQN	235/642, 448/499		DNA-binding	Homeobox associated proteins	
POP	E	sp	WTTVW/TVWTD, HVWHM/VWHMP/WHMPA, GHPWG/HPWGN, PFMRW/FMRWR/MRWRD, WNIGI	~430		No feature	Ribulose bisphosphate carboxylase (RuBisCO)	
POP	E	sp	HRAMH	~430		binding, active site	Ribulose bisphosphate carboxylase (RuBisCO)	RuBisCO catalyzes the first major step in corbon fixation in the Calvin Cycle [ref ST3]
POP	E	sp	VYPWT/YPWTQ	403/377		No feature		Part of variuos hemoglobin subunits
POP	E	sp	QRIHT	669		Zinc finger		Mainly found in human proteins
POP	E	g	MWDCM	198	23	Repeat	Sodium chanel proteins	
POP	E	g	FHWCC	283	29	No feature		Found in proteins in the Wnt singalying pathway
POP	E	g	WCCYV	207	25	No feature		Found in proteins in the Wnt singalying pathway
POP	E	g	CDQYW	180	24		Tyrosine-protein phophatases	
POP	E	g	WWDHF	569	3	No feature	All but 6 are putative retrotransposons in rice	Not found in Swissprot, High abundance only from a few species
POP	E	g	WCMRH	313	13	No feature	All but 14 are putative retrotransposons in rice	Not found in Swissprot, High abundance only from a few species
POP	E	g	YCKWH	203	3	No feature	Proteins mainly part of the retrotranspons family	High abundance only from a few species
NEP	E	sp	CSCCC	40 in rand.		Composional bias		Several consecutive cysteines
NEP	E	g	RCDLM	50 in rand.		No feature		
ORP	A	sp	WRCKT	23		No feature	Isoluecyl- and valyl-tRNA synthetase	
ORP	A	sp	RYWGI	16		No feature	Isoluecyl- and valyl-tRNA synthetase	
ORP	A	sp	TAWNK	12		Motif	Isoleucyl-tRNA synthetase	
ORP	A	sp	HHNTD	19		No feature	S-adenosylmethionine synthetase	
ORP	A	sp	DPHKM/PHKMG	12		binding	L-tyrosine decarboxylase	
ORP	A	sp	PHTSC/HTSCG	10		metal	Acetyl-CoA decarboxylase/synthase	
ORP	A	sp	RKMHT	12		active site	Glutamyl-tRNA amidotransferase subunit D	
ORP	A	g	EMCCH/MCCHY/CCHYD	18		No feature		All in same protein and same species*Methanospirillum hungatei JF-1*
ORP	B	sp	FRCGF	268		No feature		
ORP	B	sp	FGRFC	245		No feature	GTP-binding protein lepA	
ORP	B	sp	DWMEQ	265		No feature	Elongation factor G	
ORP	B	sp	YHDVD	235		No feature	Elongation factor G	
ORP	B	sp	MGAQM	234		No feature	60 kDa chaperonin	Protein also found among POP-B, not found in eukaryotes
ORP	B	sp	MNPMD	210		No feature		
ORP	B	sp	CDKIT	132		metal	Dihydroxy-acid dehydratase	The protein acts in the final step in the biosynthesis of isoleucine caline in bacteria [ref ST4]
ORP	B	sp	SCSGM	110		metal	Dihydroxy-acid dehydratase	The protein acts in the final step in the biosynthesis of isoleucine caline in bacteria [ref ST4]
ORP	B	sp	CGRYE	124		binging	M1G-methyltransferase	Bacterial specific tRNA modifying enzyme. Essential for the correct reading during translation [ref ST5]
ORP	B	sp	GHYEG	104		binging	M1G-methyltransferase	Bacterial specific tRNA modifying enzyme. Essential for the correct reading during translation [ref ST5]
ORP	B	g	FCDWY	140	138	No feature	Valyl-tRNA synthetase	The bacterial form of valyl-tRNA synthetase
ORP	B	g	HYNWH	216	9	Domain	Transposase	205 copies of transposase in*Bordatella perussis Thoma I*, most frequent
ORP	B	g	IMTWM	190	7	No feature	Transposase and pencilin binding protein	184 copies of transposase in*Mycobacterium ulcerans Agy99*
ORP	B	g	EFWCR	109	8	No feature		Mulicopy tranposase protein in*Yersinia pestis* and *Salmonella enterica*
ORP	E	sp	FAFHF/AFHFI/FHFIL, IRYMH/RYMHA/YMHAN	~1000		metal	Cytochrome b protein	Several also found in swissprot POP
ORP	E	sp	KIWFQ/WFQNR/QNRRM	448/642/317		DNA-binding	Homeobox associated proteins	Also found in POP
ORP	E	sp	YPWTQ/PWTQR	377/386		No feature	Hemoglobin	Hemoglobin is a typical eukaryotic specfic family
ORP	E	sp	HYCRD	372		No feature	RuBisCO	Different pattern from those of the RuBisCO associated POP patterns
ORP	E	sp	PIVMH/VMHDY	388		No feature	RuBisCO	Different pattern from those of the RuBisCO associated POP patterns
ORP	E	g	ECKQC	10768	34	Zinc finger		
ORP	E	g	CPCNK	405	22	covalently libid binding	Synaptosomal-associated 25 kDa protein	
ORP	E	g	WGCFD	379	41	No feature	Dynein	
URP	A	sp	QQQQQ	9113 in E+B		compositional bias		2226 if overlaps are not counted, not signficant
URP	A	sp	PPPPP	5681 in E+B		compositional bias		2533 if overlaps are not counted, not signficant
URP	A	sp	NNNNN	2215 in E+B		compositional bias		615 if overlaps are not counted, not signficant
URP	A	sp	AYAIL/AILRS	1641/1527 in E+B		No feature	Mostly found in cytochrome b	Two most signficant (p <= 0.16 and 0.017, resp.)
URP	A	g	THTGE	13209 in E+B	B:63 E:44	Zinc finger	Found in many different proteins	
URP	A	g	HRDLK	6019 in E+B	B:126 E:50	Domain and active site	Found in many different proteins	Found in 50 of the 52 eukaryotic genomes, and 126 of 303 bacterial genomes.
URP	A	g	TAGQE	3116 in E+B	B:112 E:49	NP binding	Found in many different proteins	Found in 49 of the 52 eukaryotic genomes, and 112 of 303 bacterial genomes.
URP	A	g	LHYAA	2312 in E+B	B:128 E:47	Repeat		Found in 47 of the 52 eukaryotic genomes, and 128 of 303 bacterial genomes.
URP	A	g	LPGPP	3423 in E+B	B:135 E:42	Region	Mostly found in collagen associated proteins	

Category	Kingdom	Data set	Pattern	Numer of occurrences	Number of species	Feature	Protein family	Note
URP	A	g	DVNDN/NDNAP	3865/3470 in E+B	B:63/72 E:44/40	Domain and topo domain	Cadherin-associated proteins	Patterns are part of the extracellular domain of membrane bound cadherins that contains characteristic repeats involved in the cell-cell adhesion [ref ST6]
URP	B	sp	AYVAY/YVAYP, FCAEA	348/432,379 in A+E		No feature	RuBisCO	
URP	B	sp	LRLSC/RLSCA	492/332 in A+E		No feature	Found in immunoglobin heavy chain and maturase K etc	
URP	B	sp	GHPIS	398 in A+E		No feature	Maturase K	Associated to a protein for intron splicing in plants
URP	B	sp	RNLSH	333 in A+E		No feature	Maturase K	Associated to a protein for intron splicing in plants
URP	B	sp	WDTAG	503 in A+E		NP binding	Chlorophyll binding protein + others	Plant associated
URP	B	g	MCVDY	1094 in A+E	E:10	No feature	Retrotranposon	Retrostransposon, primarily found in rice.
URP	B	g	TYMCE/MYCEA	412/435 in A+E	E:4/15	Region/domain	Retrotranposon	Retrostransposon, primarily found in rice.
URP	B	g	HHCPW	525 in E + 0 in A	E:48	Zinc finger	Palmytoyltransferase	Part of an known DHHC tetrapeptide motif.
URP	E	sp	YAEGY	270 in A+B		No feature	Serine hydroxymethyltransferase	Most abundant in other kingdoms.
URP	E	sp	VMPQT	223 in A+B		Region	Translation initiation factor IF-2	
URP	E	sp	GSHYD/YHDVD	200/235 in A+B		No feature	Elongation factor G	
URP	E	g	GWMHD	110 in A+B	A:2 B:100	No feature	1,4-alpha-glucan branching enzyme	Is widespread in the bacterial kingdom and found in 100 of 303 bacterial genomes.
URP	E	g	QWAYA	133 in A+B	A:2 B:37	No feature	UDP-N-acetylmurate-L-alanine ligase	Found in proteins that synthetize peptidoglycan murein for the bacterial cell wall.
URP	E	g	FCDWY	140 in A+B	A:0 B:138	No feature	Valyl-tRNA synthetase	Found in many bacterial genomes.
URP	E	g	WGGWW	119 in A+B	A:0 B:86	Transmembrane	Cytochrome c associated	Membrane associated
URP	E	g	PFHMW	110 in A+B	A:5 B:95	No feature	NADH-ubiquinone oxidoreductase chain N	A pattern found in many bacterias, present in NADH-ubiquinone oxidoreductase chain N, a protein which is part [illegible] the proton-pumping complex I required for ATP-synthesis. The pattern matches the quinone motif in the fourth cytoplasmic loop and the histidine has been shown to be an active site residue by mutational analysis [ref ST7]. The complex is found also in the eukaryotic mitochondria. However this pentapeptide is unique to the bacterial forms and is found in 95 of the 303 bacterial species in the genom set.
URP	E	g	IMTWM	190 in A+B	A:0 B:7	No feature	Transposase and pencilin binding protein	Also found in ORP-B, most hits to transposase motifs in Mycobacterium ulcerans Ahy99

References

ST1 Yun M, Park CG, Kim JY, Park HW: Structural analysis of glyceraldehyde 3-phosphate dehydrogenase from Escherichia coli: direct evidence of substrate binding and cofactor-induced conformational changes. Biochemistry 2000, 39(35):10702-10.

ST2 Howell N: Evolutionary conservation of protein regions in the protonmotive cytochrome b and their possible roles in redox catalysis. J Mol Evol 1989, 29(2):157-69.

ST3 Spreitzer RJ, Salvucci ME: Rubisco: structure, regulatory interactions, and possibilities for a better enzyme. Annu Rev Plant Biol 2002, 53(NIL):449-75.

ST4 MYERS JW: Dihydroxy acid dehydrase: an enzyme involved in the biosynthesis of isoleucine and valine. J Biol Chem 1961, 236(NIL):1414-8.

ST5 Ahn HJ, Kim HW, Yoon HJ, Lee BI, Suh SW, Yang JK: Crystal structure of tRNA(m1G37)methyltransferase: insights into tRNA recognition. EMBO J 2003, 22(11):2593-603.

ST6 Halbleib JM, Nelson WJ: Cadherins in development: cell adhesion, sorting, and tissue morphogenesis. Genes Dev 2006, 20(23):3199-214.

ST7 Amarneh B, Vik SB: Mutagenesis of subunit N of the Escherichia coli complex I. Identification of the initiation codon and the sensitivity of mutants to decylubiquinone. Biochemistry 2003,42(17):4800-8.
