# Supplementary material for: Characterization of oligopeptide patterns in large protein sets
Source: BMC Genomics. 2007 Oct 1;8:346. doi: 10.1186/1471-2164-8-346 (PMC2231379; doi:10.1186/1471-2164-8-346)
Supplement: Additional file 3 — Top 100 peptides of each category in Swiss-Prot data set. Data for peptide classes of the Swiss-Prot data set. The table is provided as PDF. Each peptide category (POP, NEP, ORP and ORP) is shown on a separate page. Each peptide category has three columns for the kingdoms archaea (A), bacteria (B) and eukaryota (E). Each kingdom has five columns; 1. Aligned peptide patterns. 2. For POP and ORP, the number of occurrences in original data for that kingdom; for NEP, the number of occurrences in randomized data for that kingdom; for URP, the number of occurrences in original data for the other two kingdoms. Most extreme values are color-coded with green background. 3. The p-value for biological significance (see Methods section for details). Significant values are color-coded with orange background (p ≤ 0.05). 4. The number of individual sequence region hits in Swiss-Prot release 51.5. 5. Swiss-Prot sequence features (FT field) and the fraction of sequence hits in column 4 that are mapped to this feature. Only features of at least 20% coverage are reported. Features with more than 50% coverage are color coded with background in magenta. [file 1471-2164-8-346-S3.pdf]

Swiss-Prot

POP

A

--LIKNG--	33	0	236				
--LKKEG--	32	0	360				
--YERAG--	45	0	160				
----RAGAD	29	0	324				
--SCNTT--	31	0	142	REGION:	0.21	ACT_SITE:	0.21
-VSCNT---	30	0	116	ACT_SITE:	0.31	REGION:	0.26
VVSCN----	28	0	101	REGION:	0.28	ACT_SITE:	0.28
--IDEHD--	29	0	62				
-FIDEI---	32	0	723				
--GSGKT--	117	0	4513	NP_BIND:	0.35	DOMAIN:	0.24
-FGSGK---	58	0	200				
-NGSGK---	34	0	1104	NP_BIND:	0.33	DOMAIN:	0.23
--PGTGK--	77	0	1033	NP_BIND:	0.38		
--PGVGK--	58	0	996	NP_BIND:	0.32		
--STGGT--	36	0	520				
-ISTGG---	34	0	450				
-VVTGG---	32	0	283				
--GKTVT--	32	0	434				
DHGKT----	62	0	1333	NP_BIND:	0.39		
--FPGLV--	37	0	137	REPEAT:	0.26		
---PGLVY-	28	0	89	REPEAT:	0.35		
-QFPGL---	41	0	91	REPEAT:	0.45		
--GMDKM--	29	0	54				
----DKMLV	33	0	134				
--GHVDH--	58	0	1247	NP_BIND:	0.39		
--GHVDF--	30	0	1642	NP_BIND:	0.48		
--TPGHV--	35	0	1550	NP_BIND:	0.50		
---PGHVD-	30	0	1606	NP_BIND:	0.49		
--SGKST--	57	0	4298	NP_BIND:	0.35	DOMAIN:	0.24
---GKSTL-	80	0	6701	NP_BIND:	0.37	DOMAIN:	0.24
---GKSTF-	30	0	1033	NP_BIND:	0.36		
--VGKTT--	40	0	522	NP_BIND:	0.34		
--SGKTT--	66	0	2215	NP_BIND:	0.39		
---GKTTT-	44	0	2032	NP_BIND:	0.46		
--MSKSL--	34	0	1890	MOTIF:	0.32	BINDING:	0.32
-KMSKS---	82	0	3937	MOTIF:	0.33	BINDING:	0.33
--MSKSK--	41	0	1217	BINDING:	0.32	MOTIF:	0.32
--PSTRT--	37	0	199				
---STRTR-	36	0	617	REGION:	0.33		
--CPECG--	42	0	427	ZN_FING:	0.33		
--CPYCG--	42	0	155	METAL:	0.31		
--CPNCG--	32	0	186				
--CPKCG--	77	0	317				
--CPRCG--	42	0	278	METAL:	0.27		
--CPVCG--	69	0	369				
--PPGTG--	43	0	802	NP_BIND:	0.37		
-GPPGT---	38	0	901	NP_BIND:	0.32		
--EPEQF--	37	0	103	REPEAT:	0.36		
-YEPEQ---	38	0	93	REPEAT:	0.40		
--IDTPG--	52	0	2055	NP_BIND:	0.44		
-LIDTP---	34	0	665	NP_BIND:	0.42		
--QFHPE--	71	0	2928	ACT_SITE:	0.50	DOMAIN:	0.26
-VQFHP---	36	0	1187	ACT_SITE:	0.33	DOMAIN:	0.32
-TQFHP---	29	0	308	DOMAIN:	0.33	ACT_SITE:	0.31
--AARNA--	33	0	269				
-EAARN---	29	0	243				
--VDHGK--	67	0	1410	NP_BIND:	0.40		
-HVDHG---	66	0	1390	NP_BIND:	0.40		
--DAPGH--	29	0	328	NP_BIND:	0.47		
--DTPGH--	56	0	2774	NP_BIND:	0.45		
--LGICL--	40	0	1694	DOMAIN:	0.21		
---GICLG-	54	0	1582	DOMAIN:	0.33	ACT_SITE:	0.33
--PEWRA--	29	0	61				
-GPEWR---	30	0	53				
--EQFPG--	41	0	224				
-PEQFP---	40	0	106	REPEAT:	0.37		
--KSLGN--	35	0	1643	BINDING:	0.31	MOTIF:	0.31
--NGLIT--	30	0	149				
--EGKSP--	33	0	105				
--RYKEL--	32	0	418				
--RGPAQ--	29	0	59				
--GDEVI--	34	0	309				
--RSIEG--	40	0	155				
--LDEPT--	54	0	1705	DOMAIN:	0.49		
--AKKLN--	29	0	314				
--ATPAD--	29	0	220				
--GRGNR--	30	0	115				
--RGITI--	43	0	1146				
--NKVDR--	29	0	141	NP_BIND:	0.40		
--EIVDA--	33	0	170				
--GGGSN--	34	0	410				
--QKGID--	33	0	150				
--MTDSG--	33	0	99				
--IKALK--	36	0	354				
--QADAA--	30	0	258				
--EVTPE--	30	0	197				
--IAKET--	29	0	160				
--LKEGD--	32	0	364				
--FIAMV--	31	0	68				
--RLGIP--	32	0	231				
--EAVKR--	47	0	306				
--VVYRE--	29	0	64				
--ISRQR--	38	0	314				
--AEYLA--	30	0	220				
--EKYRP--	45	0	117				
--EPVVT--	31	0	119				
--MPVAA--	30	0	108				
--TLIVF--	30	0	130				
--ALRDA--	34	0	515				
--TLAEN--	29	0	202				

B

--CPTCG--	194	0	385				
-ACPTC---	181	0	257				
--KCYGG--	300	0	313				
---CYGGD-	302	0	313				
---CYGIG-	184	0	198				
-AKCYG---	345	0	360				
--FRCGF--	268	0	268				
FGFRC----	245	0	249				
--DQWGN--	168	0	177				
---QWGDE-	217	0	708	NP_BIND:	0.33	METAL:	0.33
--IFQHF--	181	0	371	DOMAIN:	0.49		
---FQHFN-	201	0	412	DOMAIN:	0.49		
-MIFQH---	179	0	364	DOMAIN:	0.49		
--MPHPE--	197	0	442	DOMAIN:	0.46		
---PHHEN-	232	0	507	METAL:	0.50		
--TWSRR--	247	0	278				
IKTWS----	223	0	249				
--WNMGW--	162	0	163				
---NMGWM-	162	0	163				
-KWNMG---	170	0	171				
--HRHRY--	292	0	663	DOMAIN:	0.49		
---PHRYP-	173	0	178				
--LWWGH--	195	0	208				
-QLWWG---	195	0	205				
RQLWW----	195	0	214				
--HPFYT--	201	0	211				
-HHPFT---	192	0	202				
--HNMQQ--	257	0	544	DOMAIN:	0.50		
-THNMQ---	257	0	544	DOMAIN:	0.50		
---NMQRQ-	292	0	355				
--THYGR--	182	0	190				
---HYERF-	301	0	331	REGION:	0.89		
--GWHIE--	226	0	261				
---WHIEC-	225	0	501	METAL:	0.50		
----HIECS	219	0	491	METAL:	0.50		
--DWCIS--	239	0	268				
---WCISR-	262	0	295				
--IMEFC--	171	0	188				
---MEFCK-	172	0	184				
--RPGWH--	268	0	310				
---PGWHI-	228	0	265				
--DYRYF--	207	0	235				
---YRYFP-	201	0	228				
--HNGCR--	191	0	266				
---NGCRP-	187	0	258				
--YPDCR--	192	0	211				
-GYPDC---	172	0	192				
--MDWME--	254	0	259				
---DWMEQ-	265	0	267				
--RYCPS--	180	0	192				
-PRYCP---	169	0	183				
--CYGMQ--	172	0	525	DOMAIN:	0.34	ACT_SITE:	0.34
--CLGMQ--	346	0	1135	DOMAIN:	0.34	ACT_SITE:	0.34
--VWFDS--	187	0	206				
-DVWFD---	186	0	209				
--VWRQA--	349	0	368				
-TVWRQ---	335	0	341				
--GCYHG--	176	0	192				
---CYHGH-	175	0	194				
--VCTRV--	266	0	307				
---CTRVY-	212	0	264				
--MQFDR--	385	0	397				
-GMQFD---	375	0	393				
--GMNPM--	211	0	225				
---MNPMD-	210	0	213				
--TMDWM--	205	0	207				
-ATMDW---	194	0	203				
--SEMFG--	180	0	236				
--MIHQP--	273	0	606	ACT_SITE:	0.50		
--MCVND--	159	0	182				
--GCSMC--	158	0	512	METAL:	0.66		
--CYVTP--	166	0	204				
--YFVHS--	161	0	331	DOMAIN:	0.54		
--EMEVW--	258	0	327				
--CEKCG--	202	0	397				
--QRYWG--	163	0	187				
--CFRDE--	234	0	265				
--QQRLC--	269	0	583	DOMAIN:	0.49		
--GSCTN--	168	0	465	METAL:	0.45		
--QMHAN--	178	0	180				
--YYQPE--	181	0	570	DOMAIN:	0.32	MOTIF:	0.32
--HHNVG--	222	0	479	DOMAIN:	0.49		
--TTTPW--	224	0	287				
--HNHPS--	190	0	224				
--SYHDV--	203	0	212				
--SWGYQ--	191	0	392	ACT_SITE:	0.48		
--YNFED--	158	0	230				
--YYDYY--	173	0	557	DOMAIN:	0.32	MOTIF:	0.32
--FYMVG--	162	0	193				
--MRTFH--	253	0	288				
--QFMDQ--	282	0	309				
--TYQVP--	186	0	240				
--PVPMM--	179	0	190				
--NYFYP--	170	0	185				
--WMSHG--	160	0	322	DOMAIN:	0.50		
--QFHPE--	644	0	2928	ACT_SITE:	0.50	DOMAIN:	0.26
--FVLWK--	161	0	174				
--GMVHP--	158	0	182				
--WDVGH--	187	0	190				
--TTNSC--	205	0	229				

E

--AMHYT--	1477	0	1510						
---MHYTP-	175	0	188						
--TQDWV--	385	0	414						
YFTQD----	343	0	358						
--FQQMW--	259	0	287						
---QQMWI-	255	0	283						
-TFQQM---	262	0	281						
--IRYMH--	486	0	976	METAL:	0.49				
---RYMHA-	508	0	1022	METAL:	0.50				
----YMHAN	501	0	1008	METAL:	0.50				
--WWNFG--	1583	0	1600						
-SWWNF---	764	0	769						
SAWWN----	530	0	538						
--QWIWG--	241	0	246						
---WIWGG-	1318	0	1334						
-VQWIW---	239	0	246						
--TNWDD--	239	0	255						
---NWDDM-	302	0	316						
----WDDME	303	0	315						
--VTHIC--	901	0	907						
----HICRD	1364	0	1379						
--VWFQN--	235	0	480	DNA_BIND:	0.49				
---WFQNR-	642	0	1305	DNA_BIND:	0.49				
--KIWFQ--	448	0	902	DNA_BIND:	0.49				
---IWFQN-	499	0	1011	DNA_BIND:	0.49				
--WLIRY--	534	0	547						
--WLIRN--	315	0	331						
-GWIIR---	357	0	363						
--LHWYT--	214	0	217						
-FLHWY---	215	0	221						
---HWYTG-	199	0	206						
--WSYEY--	226	0	455	TOPO_DOM:	0.50				
WYWSY----	232	0	470	TOPO_DOM:	0.49				
--WTTVW--	425	0	470						
----TVWTD	428	0	480						
--GMHFR--	266	0	281						
-HGMHF---	266	0	270						
NHGMH----	258	0	269						
--VWHMP--	426	0	486						
-HVWHM---	431	0	492						
---WHMPA-	428	0	492						
--GHPWG--	424	0	499						
---HPWGN-	414	0	465						
----PWGQM	1648	0	1676						
--MRWRD--	437	0	457						
-FMRWR---	448	0	480						
PFMRW----	446	0	488						
--WLPFF--	168	0	342	TRANSMEM:	0.48				
-CWLPF---	194	0	410	TRANSMEM:	0.47				
--KIWHH--	282	0	294						
---IWHHT-	273	0	287						
--YPWTQ--	377	0	409						
-VYPWT---	403	0	463						
--QMSFW--	1628	0	1642						
---MSFWG-	1532	0	1546						
--WHHTF--	275	0	291						
---HHTFY-	278	0	293						
--MWISK--	212	0	237						
-QMWIS---	207	0	228						
--YKETW--	312	0	322						
--CDVDI--	208	0	262						
--EYFRD--	250	0	634						
--VMHDY--	388	0	399						
--WTQRF--	316	0	347						
--WMAPE--	270	0	694	DOMAIN:	0.39				
--EWYFL--	1615	0	1630						
--MNEPP--	187	0	494						
--AYDRY--	225	0	890	TOPO_DOM:	0.25	DISULFID:	0.22	TRANSMEM:	0.22
--FDKCI--	175	0	391	METAL:	0.47				
--CEEMI--	202	0	409	DISULFID:	0.49				
--MFRPF--	168	0	187						
--MYVAI--	235	0	256						
--PMYFF--	276	0	574	TRANSMEM:	0.47				
--WNIGI--	506	0	523						
--MEKIW--	295	0	310						
--QNRRM--	317	0	645	DNA_BIND:	0.48				
--YGCYC--	252	0	1139	DISULFID:	0.53	METAL:	0.21		
--NHSFM--	242	0	735	METAL:	0.33	TOPO_DOM:	0.33		
--CEVWK--	304	0	311						
--IHVWH--	430	0	484						
--HYYSG--	265	0	266						
--YFPHF--	241	0	272						
--ITRMY--	200	0	205						
--QCLFW--	548	0	549						
--HQWYW--	262	0	528	TOPO_DOM:	0.50				
--YWVKD--	233	0	241						
--CIFCG--	174	0	843	METAL:	0.65				
--QRIHT--	669	0	940	ZN_FING:	0.71				
--QCGNQ--	247	0	256						
--LAHYC--	331	0	339						
--NYGWI--	393	0	400						
--PYKCN--	218	0	334	ZN_FING:	0.64				
--RCYHI--	266	0	270						
--FHDHT--	167	0	333	TRANSMEM:	0.49	TOPO_DOM:	0.49		
--MIPFH--	208	0	213						
--CIYLH--	168	0	335	METAL:	0.50				
--NKWKY--	188	0	191						
--VEYCP--	167	0	368	METAL:	0.48				
--HRAMH--	435	0	1486	BINDING:	0.33	ACT_SITE:	0.33		
--CPTNC--	168	0	387	METAL:	0.48				

Swiss-Prot

NEP

A

--LLVRL--	16	0	188		
--LLVAE--	16	0	308		
---LVALI-	18	0	463	TRANSMEM:	0.31
--IILIK--	15	0.01	43		
---ILLKE-	15	0	202		
--IVLLK--	18	0	192		
--GGKEL--	19	0.01	148		
-AAGKE---	16	0.02	169		
--EGEAS--	20	0.03	125		
---GAASL-	16	0.03	311		
--LLEED--	16	0	350		
--LVEDE--	15	0	153		
--AGEGL--	23	0.01	265		
ERAGE----	15	0.02	111		
-DAGEK---	16	0.08	85		
--AEILL--	18	0	228		
---EIELV-	17	0	120		
--EIALI--	16	0	147		
--EIVLI--	15	0	118		
--EIGLL--	15	0	223		
--IEKKK--	20	0	119		
--YEKKL--	15	0.06	147		
--VGLIE--	15	0.01	94		
AAVGL----	15	0	561		
--KLEVL--	15	0	280		
-LKLEV---	19	0	177		
---LEVEP-	16	0.05	114		
--KILLL--	18	0	510		
-VKILI---	17	0	81		
--VLIII--	15	0	132	TRANSMEM:	0.33
---LIIIV-	18	0	116	TRANSMEM:	0.41
---LIIII-	23	0	199	TRANSMEM:	0.39
--ILIII--	17	0	297	TRANSMEM:	0.34
---LTIIV-	17	0.04	195	TRANSMEM:	0.34
--LSIII--	16	0	165	TRANSMEM:	0.25
--LEIII--	16	0.01	124		
--EELRL--	15	0	431		
--EETKL--	15	0.04	146		
--KFLII--	16	0.05	37		
---VLIIV-	16	0	189	TRANSMEM:	0.33
--LILEI--	16	0	141		
--LELRI--	15	0.01	91		
--EVLVL--	15	0	272		
--RLLVL--	15	0	379		
--AEGVG--	17	0	197		
--EEDVG--	18	0.01	105		
--EIVIE--	15	0	62		
--EIVII--	16	0	46		
--LIVIL--	19	0.01	284	TRANSMEM:	0.36
--RGELI--	16	0.03	78		
--RLELV--	15	0.01	179		
--LILLE--	16	0	254		
--LILLV--	15	0	518	TRANSMEM:	0.30
---ILLED-	16	0	195		
--LVLLE--	15	0	345		
--IIIIV--	17	0	168	TRANSMEM:	0.43
--ILIIV--	17	0	216	TRANSMEM:	0.28
----IIVEL	15	0	69		
--VGALE--	20	0	181		
-EVGAE---	17	0	178		
---GAEEE-	16	0	217		
--KPKIE--	18	0.08	74	REGION:	0.27
---IKIEI-	19	0	48		
--LKIVV--	17	0.01	99		
-EKKIV---	15	0	132		
--VVLED--	16	0.01	228		
-LVVIE---	16	0	131		
--LLLIE--	19	0	121		
-KLLVI---	15	0	120		
--LLIKL--	17	0	165		
--LIIKL--	16	0	179		
--LLIIE--	20	0	60		
--LLIIK--	17	0	92		
--IVVIV--	16	0	152		
--IVLIV--	15	0	183	TRANSMEM:	0.37
--LVIVI--	18	0	161	TRANSMEM:	0.40
-VLVIV---	17	0	340	TRANSMEM:	0.36
--ILVLE--	19	0	133		
--ILILE--	15	0	101		
--LVIEI--	15	0	54		
--LIIEI--	19	0.01	54		
--ALTEE--	16	0.04	291		
--AATEE--	15	0.01	244		
--DGVVG--	17	0.02	89		
--ILTIK--	15	0.04	85	REGION:	0.25
--KEEER--	17	0	268		
--EKGAL--	17	0.03	220		
--EAAAI--	19	0.03	320		
--EPIGK--	15	0.13	45		
--ALKVV--	15	0.01	200		
--ETELE--	15	0.01	214		
--ILGVA--	15	0.01	316		
--EGEGI--	16	0	127		
--LKLIL--	15	0	230		
--VREGA--	15	0.03	245		
--VLIEL--	17	0	92		
--IIETK--	15	0.02	112		
--KKKEA--	16	0.01	208		
--GARGE--	15	0.02	194		
--IIIKI--	17	0	42		

B

--GLGPP-	53	0	126				
--GVGPP-	37	0	62				
---LGPPT	37	0	71				
--ESPRA-	35	0	72				
ARESP---	34	0	42				
--EPVGK-	36	0	58				
-VEPPG--	35	0	41				
--VIVLY-	35	0	54	TRANSMEM:	0.28		
---IVLKF	50	0	11				
---IVLRM	35	0	136	TRANSMEM:	0.29	DOMAIN:	0.28
--GQKDE-	37	0	34				
--GPEDE-	41	0	74				
--TAHLV-	41	0	32				
-KRAHL--	35	0	22				
--TRKEG-	40	0	34				
---RKETT	36	0	29				
--LYIRA-	35	0	26				
-YLYIL--	34	0	19				
-LLYIK--	46	0	42	DOMAIN:	0.21		
--FVIIL-	57	0	55	TRANSMEM:	0.29		
--FVIKI-	37	0	22	DOMAIN:	0.23	TOPO_DOM:	0.23
--KIEGP-	37	0	17	COMPBIAS:	0.24		
--KNEGG-	38	0	31				
--AAVHI-	40	0	32				
-ILAVH--	46	0	32				
--NKTAG-	36	0	43				
--NKSAG-	37	0	35				
--NEGAG-	47	0	33				
--FIIKV-	39	0	44	DOMAIN:	0.36		
--FIIKI-	44	0	36	TOPO_DOM:	0.28	DOMAIN:	0.22
-IFIVK--	39	0	65	DOMAIN:	0.29		
--GEDNT-	37	0	18				
--GEDRT-	37	0	28	TOPO_DOM:	0.21		
---EDTTG	35	0	51				
--RAPPE-	34	0	73				
-VRAPP--	37	0	43				
-AQAPP--	40	0	132				
--ARSPP-	36	0	81				
--ARAPP-	50	0	106				
---RGPPA	49	0	77				
--MLLVK-	36	0	58				
--MILVR-	35	0	11	TRANSMEM:	0.36		
--TRASP-	36	0	36				
--DRAEP-	35	0	11				
---RAGPP	34	0	92				
--PIDVG-	48	0	15				
--PIKGG-	55	0	16				
--DANED-	35	0	31				
--DAQSD-	36	0	16				
--ARGPP-	35	0	161				
--ARGDP-	49	0	76				
--PRGAP-	35	0	122				
--IIIRV-	50	0	23				
-VIIIK--	54	0	13				
--GPREE-	40	0	54				
-EEPRE--	35	0	42				
--EDTVD-	39	0	42				
---DTKDR	37	0	20				
--GPPLD-	38	0	76				
---PPLFL	37	0	144				
--VVYIL-	36	0	49				
--VVYID-	34	0	36				
--IYIRL-	35	0	26	TOPO_DOM:	0.35		
--IYIKL-	48	0	21				
--IIIRI-	41	0	18	DOMAIN:	0.22		
--IEIRI-	37	0	13	VAR_SEQ:	0.23		
--KIGMG-	35	0	25				
--GPAES-	44	0	67				
--KLVIY-	35	0	32				
--LFIVR-	38	0	118	DOMAIN:	0.28	TRANSMEM:	0.27
--VYVIL-	43	0	29	TRANSMEM:	0.28		
--FAVGP-	37	0	33				
--GGTNE-	38	0	17				
--EGSLM-	35	0	43	TOPO_DOM:	0.28		
--GDEGN-	39	0	21				
--NKEEP-	35	0	22				
--ELEMG-	35	0	23				
--AEPRK-	38	0	55				
--MAPGG-	39	0	31				
--ARDQS-	36	0	19	REGION:	0.26		
--VLIQF-	37	0	51				
--ETIGP-	36	0	22				
--LIVYE-	37	0	39	REGION:	0.36		
--AHIIV-	35	0	45	DOMAIN:	0.24		
--DMLAI-	45	0	13				
--KEQDG-	36	0	21				
--IGVRK-	37	0	36				
--NSVDG-	36	0	38	TOPO_DOM:	0.24		
--NVGIE-	44	0	10				
--DQGDG-	37	0	24	DOMAIN:	0.25		
--SEVGY-	34	0	18				
--LMVEI-	35	0	22				
--FILIP-	34	0	27	TRANSMEM:	0.26		
--GGVQD-	46	0	19	TOPO_DOM:	0.37		
--DTEGD-	35	0	27				
--PIQIL-	39	0	46				
--EKDNA-	38	0	77	COILED:	0.34		
--GKASQ-	39	0	52	DOMAIN:	0.33		
--DKTED-	38	0	43				
--VHLKA-	37	0	37				

E

--PDKGP--	28	0	13		
-GPDKP---	36	0	10		
--AYGPL--	30	0	30		
--AIYPL--	29	0	57		
--IDPGP--	31	0	27		
--IDPGG--	30	0	7	TRANSMEM:	0.29
--NDPGG--	28	0	7		
--AGPNK--	29	0	6		
-GIGPN---	29	0	24		
--RAGML--	32	0	103		
----GMLES	32	0	18		
--GYARE--	28	0	32		
----AREPT	30	0	10		
--IMLKS--	31	0	27		
--IMLDL--	35	0	25		
NLIML----	29	0	6		
--FKIEA--	28	0	25		
--FPREA--	33	0	19		
--LGVEH--	28	0	34		
-IQGVE---	28	0	32		
--LMTGT--	32	0	59		
-LTMTG---	30	0	27		
--NDQGL--	30	0	27		
---DAGLM-	28	0	17		
--AQFGV--	28	0	33		
----FGVRD	29	0	34		
--YLVVM--	29	0	3	DOMAIN:	0.33
--YLILM--	29	0	5		
--LLIEW--	28	0	15		
-KLHIE---	28	0	16		
--APNQS--	28	0	8		
--EPNQS--	28	0	13		
--GPNIS--	29	0	1		
--GPKQS--	30	0	9	REGION:	0.22
--RFFEL--	30	0	25		
SARFF----	30	0	40		
--CELKL--	32	0	13		
--CNLKL--	30	0	4		
--VEVGY--	29	0	14		
--VDTGY--	28	0	51		
--VLYIE--	29	0	20		
---LYFEL-	38	0	15		
--TEVYL--	31	0	44		
---GVYLK-	32	0	18		
--FMLEL--	29	0	13		
--FFLEQ--	30	0	9	VAR_SEQ:	0.22
--HLIII--	29	0	13		
--KFIII--	40	0	18		
--ESPML--	34	0	10		
--GKIGQ--	31	0	20		
--SKYVT--	33	0	28		
--TELMI--	29	0	6		
--QERDD--	30	0	24		
--KGLHA--	29	0	34		
--ARDTK--	32	0	177		
--CELDS--	29	0	13		
--LCKLT--	30	0	9		
--EYQSE--	29	0	9		
--QVSFP--	30	0	14		
--FMSLK--	30	0	7		
--SEYRG--	28	0	19		
--TKGVR--	32	0	23		
--GEPNE--	29	0	18		
--SMLAF--	31	0	37		
--VGIEQ--	29	0	29		
--DKQGA--	32	0	25		
--CSCCC--	40	0	5	COMPBIAS:	0.40
--SDPPD--	36	0	4		
--PRFKA--	28	0	38		
--PTYVS--	36	0	17		
--AIAFE--	32	0	42		
--GLQTY--	32	0	14		
--NPVGR--	28	0	27	TOPO_DOM:	0.44
--KRAQP--	28	0	16		
--DTQAD--	28	0	25		
--FIAVR--	29	0	40		
--PARNE--	28	0	14		
--LCPVE--	28	0	13		
--LATPH--	29	0	40		
--REVKF--	28	0	12	VAR_SEQ:	0.25
--LTFRF--	29	0	21		
--HKESI--	32	0	57		
--GMGSE--	31	0	16		
--LNIKM--	28	0	26		
--LMPDL--	29	0	9		
--DLAYD--	28	0	17		
--PKNEG--	28	0	8		
--LRFQG--	31	0	17		
--GKSHA--	28	0	32	NP_BIND:	0.31
--LIRIY--	32	0	35		
--LFKFA--	33	0	10		
--SEPYA--	30	0	10		
--KQDRS--	28	0	15		
--MFVEL--	30	0	15		
--EVFIL--	31	0	41		
--GQDDA--	34	0	55		
--RNNAS--	31	0	27		
--GIRTI--	31	0	70		
--ARPLM--	28	0	16		
--IILYK--	32	0	12		

Swiss-Prot

ORP

A

-IWKGD--	10	0	14		
-IWKYT--	11	0	20	DOMAIN:	0.35
-ARWYC--	13	0	20		
--RWYCK-	10	0	15		
---WYCKE	11	0	19		
-WEMRV--	11	0	13		
HWEMR---	12	0	14		
-DPHKM--	12	0	25	BINDING:	0.48
--PHKMG-	12	0	25	BINDING:	0.48
-HTSCG--	10	0	23	METAL:	0.39
PHTSC---	10	0	20	METAL:	0.45
-DTWMD--	11	0	11		
--TWMDS-	11	0	13		
-WYPLD--	10	0	14		
-VYIGC--	12	0	13		
-RYWGI--	16	0	16		
-WYNGP--	12	0	15		
-YHYDH--	11	0	13		
-ECQAM--	11	0	12		
-HMSYA--	12	0	17		
-FIEWQ--	10	0	13		
-WMTWK--	10	0	17	HELIX:	0.24
-WRCKT--	23	0	23		
-YPILW--	10	0	24	TRANSMEM:	0.46
-YHDPC--	10	0	12		
-MTSQC--	11	0	13		
-EVYWV--	11	0	16		
-QPCIR--	11	0	11		
-MQIGM--	10	0	17		
-CFREV--	12	0	26	METAL:	0.46
-IYMAY--	10	0	18		
-WFYSQ--	12	0	14		
-ACPHR--	12	0	13		
-HLTMY--	10	0	12		
-TAWNK--	13	0	30	MOTIF:	0.43
-WCGRG--	11	0	24	REGION:	0.50
-VNHCV--	10	0	11		
-HHNTD--	19	0	20		
-HLCHV--	14	0	15		
-MMAHH--	10	-	10		
-ISHIM--	10	0	14		
-QGDHR--	14	0	17		
-KHKYD--	10	0	14		
-GFTQY--	11	0	17		
-WVMDR--	10	0	12		
-FGKWD--	11	0	14		
-YITWG--	10	0	11		
-RKMHT--	12	0	29	ACT_SITE:	0.41
-WRVDW--	11	0	15		
-QGWDC--	10	0	13		
-VWKLY--	11	0	16		
-KVFWA--	13	0	15		
-VCQQN--	11	0	13		
-KINSC--	10	0	21	DISULFID:	0.24

B

--RMEFP--	106	0	109				
-NIMEF---	178	0	184				
---MEFCK-	172	0	184				
--TQWGD--	111	0	330	NP_BIND:	0.33	METAL:	0.33
-SDQWG---	144	0	155				
--QWGNI--	139	0	145				
-DQWGN---	168	0	177				
--PGCSM--	130	0	255	METAL:	0.49		
--HFCSM--	142	0	118				
--YQIQT--	109	0	109				
RYYQI----	125	0	131				
--CGRYE--	124	0	255	BINDING:	0.49		
---GHYEG-	104	0	201	BINDING:	0.45		
--FEFRW--	109	0	110				
--FGFRC--	245	0	249				
--MIEGP--	125	0	129				
--MIGDP--	132	0	138				
--ENRFM--	143	0	145				
----RFMHV	120	0	127				
--MGWMH--	105	0	105				
-NMGWM---	162	0	163				
-TMDWM---	205	0	207				
--FHTFN--	115	0	120				
---HDFND-	109	0	114				
--WNGYN--	119	0	121				
-PWNGY---	112	0	113				
MPWNG----	106	0	107				
--RDVHP--	147	0	155				
----VHPTH	118	0	119				
--VGHET--	116	0	118				
---GHQTR-	119	0	128				
--ANMQR--	144	0	150				
-GANMQ---	145	0	148				
IQANM----	119	0	124				
--RHHTF--	139	0	142				
---HHHFE-	150	0	159				
--VAHIW--	127	0	134				
---AHIWF-	132	0	140				
----HIWFL	129	0	135				
--GWMHD--	122	0	124				
--GSYHD--	200	0	203				
--KWNMG--	170	0	171				
---WNMGW-	162	0	163				
--IDWQG--	153	0	297	DOMAIN:	0.47		
---DWQGA-	137	0	268	DOMAIN:	0.47		
--LPHRY--	142	0	149				
---PHRYP-	173	0	178				
--AFMPW--	144	0	146				
---FMPWN-	105	0	105				
--FHIGG--	124	0	135				
-TFHIG---	122	0	130				
-PTDMQ---	152	0	166				
-FPDPW---	132	0	142				
-TETVW---	114	0	116				
-HLDYH---	119	0	121				
-CDKIT---	132	0	261	METAL:	0.48		
-FCDWY---	111	0	114				
-YPAME---	151	0	155				
-PQFYF---	129	0	135				
-QRNWI---	116	0	117				
-QMHAN---	178	0	180				
-YHDVD---	235	0	238				
-FECLH---	129	0	136				
-YVPGW---	109	0	113				
-AWYSY---	121	0	125				
-RFGVM---	110	0	114				
-LEPMM---	121	0	123				
-TRMKS---	124	0	141	REGION:	0.79		
-MGAQM---	224	0	233				
-MNPMD---	210	0	213				
-DWMEQ---	265	0	267				
-YIRFA---	106	0	208	DOMAIN:	0.47		
-DHRIN---	194	0	201				
-TYNFP---	196	0	202				
-KCFRD---	118	0	125				
-VMFGN---	131	0	138				
-GEHAD---	105	0	201	BINDING:	0.42		
-RCDAN---	107	0	107				
-PHYTR---	124	0	125				
-VIKSH---	113	0	188	DOMAIN:	0.39		
-HPWFV---	104	0	209	DOMAIN:	0.49		
-VYKHA---	141	0	148				
-RFTDG---	121	0	122				
-AGAYW---	110	0	121				
-MMVGR---	136	0	273	DOMAIN:	0.48		
-MNIGQ---	185	0	189				
-QYAYR---	116	0	122				
-HGTYP---	155	0	158				
-FRCGF---	268	0	268				
-SVMQF---	107	0	109				
-KDYEC---	142	0	150				
-ITHEM---	195	0	383	DOMAIN:	0.49		
-WMSHG---	160	0	322	DOMAIN:	0.50		
-PYRKV---	156	0	159				
-PMFHQ---	126	0	134				
-SCSGM---	111	0	226	METAL:	0.48		
-MLYPT---	104	0	111				
-TRHNA---	117	0	121				
-KDHHS---	122	0	129				
-NSMNC---	124	0	128				

E

--MFFIC--	1060	0	1071		
---FFICI-	304	0	315		
---FFICL-	1087	0	1103		
----FICLY	452	0	461		
--FAFHF--	1257	0	2525	METAL:	0.50
---AFHFI-	980	0	1973	METAL:	0.50
----FHFIL	990	0	1989	METAL:	0.49
--YGWII--	347	0	352		
---GWIIR-	357	0	363		
--YGWLI--	763	0	771		
-NYGWI---	393	0	400		
--AWWNF--	533	0	540		
--SWWNF--	764	0	769		
----WNFGS	1646	0	1668		
--YKETW--	312	0	322		
---KETWN-	410	0	416		
--LETWN--	320	0	329		
---ETWNI-	1151	0	1165		
--ICLFL--	377	0	392		
---CLFLH-	472	0	958	METAL:	0.49
--QCLFW--	548	0	549		
--HCDKL--	313	0	651	METAL:	0.47
-LHCDK---	312	0	649	METAL:	0.47
---CDKLH-	312	0	349		
--QNRRM--	317	0	645	DNA_BIND:	0.48
WFQNR----	642	0	1305	DNA_BIND:	0.49
--NYTPA--	793	0	808		
PDNYT----	819	0	858		
--WGGFS--	1556	0	1588		
WIWGG----	1318	0	1334		
--VMHDY--	388	0	399		
PIVMH----	388	0	396		
--SAWWN--	530	0	538		
-ISAWW---	524	0	531		
--SSWWN--	757	0	758		
--HYCRD--	372	0	386		
--HICRD--	1364	0	1379		
--THICR--	922	0	931		
--AHICR--	406	0	425		
--IRYMH--	486	0	976	METAL:	0.49
---RYMHA-	508	0	1022	METAL:	0.50
----YMHAN	501	0	1008	METAL:	0.50
--YKCEE--	317	0	400	ZN_FING:	0.77
-PYKCE---	346	0	459	ZN_FING:	0.74
KPYKC----	996	0	1361	ZN_FING:	0.72
--PWTQR--	386	0	421		
-YPWTQ---	377	0	409		
--RLSCA--	332	0	359		
-LRLSC---	492	0	554		
--AHYCR--	330	0	361		
-LAHYC---	331	0	339		
--NMFTS--	382	0	453		
-TNMFT---	364	0	373		
--VVYPW--	306	0	363		
---VYPWT-	403	0	463		
--VTHIC--	901	0	907		
--VAHIC--	392	0	400		
--EWIWG--	1064	0	1072		
-VEWIW---	1015	0	1021		
--SECGK--	399	0	615	ZN_FING:	0.63
--EECGK--	430	0	564	ZN_FING:	0.73
--PYECK--	453	0	583	ZN_FING:	0.77
-KPYEC---	1061	0	1400	ZN_FING:	0.75
--MFRPI--	323	0	326		
-MMFRP---	816	0	827		
--MHYTA--	331	0	343		
--MHYTS--	992	0	1001		
--RIWYL--	350	0	361		
---IWYLD-	386	0	390		
--TQRFF--	317	0	351		
-WTQRF---	316	0	347		
--ISSWW--	683	0	686		
-NISSW---	653	0	660		
--RSMMF--	707	0	713		
---SMMFR-	732	0	743		
---YCRDN-	430	0	449		
---PYYTI-	1034	0	1043		
---HYVRY-	471	0	478		
---WYFLF-	1593	0	1608		
---HQRIH-	826	0	1142	ZN_FING:	0.72
---ELHCD-	331	0	694	METAL:	0.46
---THPLM-	381	0	393		
---MFETF-	307	0	339		
---YGSYM-	330	0	337		
---ACEVW-	306	0	315		
---MYQQN-	305	0	311		
---TLTWI-	898	0	909		
---WIIRY-	351	0	356		
---HPFII-	386	0	397		
---TKTYF-	308	0	335		
---ECKEC-	391	0	553	ZN_FING:	0.68
---NPIIY-	433	0	890	TRANSMEM:	0.46
---KIWFQ-	448	0	902	DNA_BIND:	0.49
---GHPIS-	398	0	414		
---WDTAG-	503	0	1071	NP_BIND:	0.42
---QGPPG-	451	0	607	REGION:	0.45
---WIGGQ-	1265	0	1282		
---RNLSH-	333	0	361		
---KQRSM-	812	0	822		
---FCAEA-	379	0	389		

Swiss-Prot

URP

A

--SFWGA--	1539	1.00	1547						
---FWGAT-	1549	1.00	1561						
----WGATV	1609	0.97	1630						
--TWIGG--	1338	0.99	1361						
---WIGGQ-	1267	1.00	1282						
-LTWIG---	1422	0.95	1448						
--KIPFH--	1262	0.98	1286						
---IPFHP-	1478	0.99	1493						
-DKIPF---	1279	0.60	1299						
--LAMHY--	1549	1.00	1581						
---AMHYT-	1484	1.00	1510						
-FLAMH---	1427	1.00	1444						
--ICRDV--	1386	0.99	1411						
---CRDVN-	1358	1.00	1383						
-HICRD---	1364	1.00	1379						
--VLPWG--	1652	0.95	1679						
-YVLPW---	1646	0.99	1667						
GYVLP----	1728	0.38	1782						
--IPNKL--	1606	0.29	1634						
-SIPNK---	1591	0.63	1606						
---PNKLG-	1598	0.46	1609						
--WNFGS--	1647	1.00	1668						
---NFGSL-	1595	0.62	1626						
-WWNFG---	1587	1.00	1600						
--PPGPP--	1291	0.87	1195	REGION:	0.50	COMPBIAS:	0.26		
---PGPPG-	1677	0.30	1065	REGION:	1.06	TOPO_DOM:	0.21		
----GPPGP	1855	0.30	1082	REGION:	1.15	TOPO_DOM:	0.25	DOMAIN:	0.22
--PPPPP--	5681	-	4058	COMPBIAS:	1.15	VAR_SEQ:	0.22		
--QMSFW--	1634	1.00	1642						
-GQMSF---	1649	1.00	1665						
---MSFWG-	1538	1.00	1546						
--LLFLH--	1373	0.65	1430						
---LFLHE-	1353	0.87	1372						
-HLLFL---	1375	0.65	2730	METAL:	0.48				
--WGGFS--	1558	1.00	1588						
--WGQMS--	1654	1.00	1662						
--PHIKP--	1343	0.99	1369						
-PPHIK---	1342	0.99	1363						
TPPHI----	1361	1.00	1377						
--FFAFH--	1262	1.00	2530	METAL:	0.50				
---FAFHF-	1258	1.00	2525	METAL:	0.50				
-RFFAF---	1266	0.96	1275						
--LTRFF--	1584	0.88	1605						
---TRFFA-	1483	0.96	1499						
-TLTRF---	1660	0.68	1688						
--LFAYA--	1540	0.57	1574						
-FLFAY---	1609	0.81	1639						
YFLFA----	1625	0.81	1653						
--VDKAT--	1281	0.19	1307						
---DKATL-	1309	0.26	1340						
----KATLT	1304	0.31	1392						
--LGDPD--	1370	0.24	1419						
-LLGDP---	1327	0.15	1567						
--AILRS--	1641	0.17	1701						
-YAILR---	1580	0.29	1606						
--PFHPY--	1513	1.00	1524						
---FHPYY-	1266	1.00	1281						
--KPEWY--	1571	1.00	1578						
---PEWYF-	1698	1.00	1714						
--ETGSN--	1399	0.50	1417						
-HETGS---	1395	0.90	1411						
--DSDSD--	1979	0.46	707	COMPBIAS:	2.28	REPEAT:	0.32		
---SDSDS-	2211	0.66	749	COMPBIAS:	2.44				
--LRSIP--	1672	0.45	1703						
---RSIPN-	1610	0.87	1630						
--IWGGF--	1329	1.00	1351						
-WIWGG---	1320	1.00	1334						
--SAIPY--	1427	0.74	1463						
-LSAIP---	1382	0.29	1465						
--FLHET--	1389	0.97	1411						
---LHETG-	1404	0.72	1431						
--GASMF--	1301	0.85	1325						
---ASMFF-	1273	1.00	1293						
--CGKAF--	2034	1.00	2546	ZN_FING:	0.79				
-ECGKA---	1346	0.79	1740	ZN_FING:	0.76				
--WYFLF--	1595	1.00	1608						
-EWYFL---	1620	1.00	1630						
--IKPEW--	1584	0.97	1593						
-HIKPE---	1580	0.82	1599						
--GSNNP--	1567	0.97	1584						
-TGSNN---	1408	0.89	1438						
--AYAIL--	1527	0.16	1542						
-FAYAI---	1541	0.68	1562						
--LPWGQ--	1655	1.00	1671						
---PWGQM-	1660	1.00	1676						
--VNYGW--	1334	1.00	1344						
-DVNYG---	1351	0.76	1361						
---YYGSY-	1600	1.00	1648						
---HANGA-	1613	0.94	3225	METAL:	0.49				
---QILTG-	1258	0.70	1359						
---QQQQQ-	9113	-	3250	COMPBIAS:	2.52	VAR_SEQ:	0.31	COILED:	0.22
---TNLLS-	1485	0.53	1561						
---SRSRS-	1421	0.39	1064	COMPBIAS:	0.89	VAR_SEQ:	0.36		
---GGQPV-	1359	0.69	1403						
---AFSSV-	1424	0.49	1477						
---PSNIS-	1331	0.83	1375						
---TVITN-	1794	0.66	1835						
---NNNNN-	2215	-	943	COMPBIAS:	2.04				
---MATAF-	1624	0.93	1642						
---IPYIG-	1318	0.57	1353						

B

--MHYTA--	332	0.28	343		
---HYTAD-	377	0.04	383		
--HYTSD--	997	0.09	1012		
-MHYTS---	993	0.51	1001		
--YGWII--	347	0.13	352		
-NYGWI---	394	0.41	400		
--YGWLI--	763	0.06	771		
--IRYMH--	486	0.58	976	METAL:	0.49
---RYMHA-	508	0.30	1022	METAL:	0.50
----YMHAN	501	0.53	1008	METAL:	0.50
--PWTQR--	386	0.46	421		
-YPWTQ---	377	0.85	409		
VYPWT----	403	0.61	463		
--VMHDY--	388	0.35	399		
PIVMH----	388	0.15	396		
--YCRDN--	430	0.77	449		
-HYCRD---	372	0.89	386		
---CRDVN-	1357	0.24	1383		
--FFICL--	1087	0.13	1103		
---FICLY-	452	0.27	461		
-MFFIC---	1060	0.69	1071		
--QCLFW--	548	0.97	549		
-SQCLF---	554	0.28	563		
--ICLFL--	377	0.01	392		
---CLFLH-	472	0.24	958	METAL:	0.49
--AHYCR--	330	0.77	361		
--AHICR--	406	0.39	425		
-LAHYC---	331	0.46	339		
--NYTPA--	793	0.01	808		
PDNYT----	819	0.07	858		
--QNRRM--	317	0.09	645	DNA_BIND:	0.48
WFQNR----	642	0.79	1305	DNA_BIND:	0.49
--VTHIC--	901	0.60	907		
--VAHIC--	392	0.24	400		
--THICR--	922	0.62	931		
---HICRD-	1364	0.55	1379		
--FHFIL--	990	0.01	1989	METAL:	0.49
-AFHFI---	980	0.02	1973	METAL:	0.50
FAFHF----	1257	0.15	2525	METAL:	0.50
--EWIWG--	1064	0.60	1072		
-VEWIW---	1015	0.69	1021		
---WIWGG-	1318	0.41	1334		
--PYECK--	453	0.60	583	ZN_FING:	0.77
-KPYEC---	1061	0.60	1400	ZN_FING:	0.75
--KPYKC--	996	0.61	1361	ZN_FING:	0.72
---PYKCE-	346	0.60	459	ZN_FING:	0.74
--AYVAY--	348	0	365		
---YVAYP-	432	0.02	456		
--HYVRY--	471	0.33	478		
--SAWWN--	530	0.83	538		
-ISAWW---	524	0.51	531		
---AWWNF-	533	0.91	540		
--RLSCA--	332	0	359		
-LRLSC---	492	0	554		
--NMFTS--	382	0.11	453		
-TNMFT---	364	0.13	373		
--GWIIR--	357	0.05	363		
---WIIRY-	351	0.34	356		
--KCEEC--	356	0.70	454	ZN_FING:	0.76
-YKCEE---	317	0.29	400	ZN_FING:	0.77
--HPLMK--	533	0.15	544		
-THPLM---	381	0.11	393		
--ETWNI--	1151	0.25	1165		
-KETWN---	410	0.30	416		
-LETWN---	320	0.14	329		
--SECGK--	399	0.09	615	ZN_FING:	0.63
--EECGK--	431	0.04	564	ZN_FING:	0.73
--ISSWW--	683	0.67	686		
-NISSW---	653	0.28	660		
--RGPPG--	417	0	514	REGION:	0.53
--QGPPG--	451	0	607	REGION:	0.45
--MFRPI--	323	0.07	326		
-MMFRP---	816	0.47	827		
--SSWWN--	757	0.97	758		
---SWWNF-	764	1.00	769		
--RIWYL--	350	0.19	361		
---IWYLD-	386	0.21	390		
--RSMMF--	707	0.31	713		
---SMMFR-	732	0.31	743		
--WNFGS--	1646	0.37	1668		
--PYYTI--	1035	0.14	1043		
--DDMEK--	321	0	354		
--WYFLF--	1593	0.42	1608		
--RGIYY--	433	0.01	447		
--HQRIH--	826	0.38	1142	ZN_FING:	0.72
--ELHCD--	331	0.29	694	METAL:	0.46
--YGSYM--	331	0.20	337		
--TLTWI--	898	0.03	909		
--HPFII--	387	0.08	397		
--ECKEC--	391	0.70	553	ZN_FING:	0.68
--NPIIY--	433	0	890	TRANSMEM:	0.46
--KIWFQ--	448	0.53	902	DNA_BIND:	0.49
--GHPIS--	398	0.01	414		
--WDTAG--	503	0.03	1071	NP_BIND:	0.42
--WGGFS--	1556	0.06	1588		
--WIGGQ--	1265	0.13	1282		
--RNLSH--	333	0.02	361		
--TQRFF--	317	0.06	351		
--KQRSM--	812	0.04	822		
--FCAEA--	379	0.04	389		

E

--LPHRY--	142	0.01	149		
---PHRYP-	174	0.06	178		
--KSHHN--	241	0.02	479	DOMAIN:	0.49
-IKSHH---	235	0.03	472	DOMAIN:	0.49
--KDHHS--	122	0.04	129		
--NIMEF--	178	0.02	184		
----MEFCK	172	0.30	184		
--MIEGP--	126	0	129		
--MIGDP--	132	0.01	138		
--RHHTF--	139	0.28	142		
---HHHFE-	150	0.39	159		
--TMDWM--	205	0.84	207		
----DWMEQ	265	0.61	267		
--WNMGW--	162	0.95	163		
---NMGWM-	162	0.80	163		
-KWNMG---	170	0.51	171		
--QWGNI--	139	0.23	145		
-DQWGN---	168	0.30	177		
SDQWG----	144	0.07	155		
--VAHIW--	127	0.27	134		
---AHIWF-	132	0.43	140		
----HIWFL	129	0.13	135		
--PLMHV--	169	0.02	173		
----MHVIA	172	0.04	177		
--HFCSM--	142	0.60	118		
---FCSMC-	146	0.50	152		
--PGCSM--	130	0.05	255	METAL:	0.49
--FRCGF--	268	0.11	268		
FGFRC----	245	0.11	249		
--IRMKI--	140	0.01	146		
--TRMKS--	125	0	141	REGION:	0.79
--YAHAR--	146	0.01	152		
-QYAHA---	146	0.06	151		
-VYKHA---	141	0.02	148		
--HNMQQ--	272	0.39	544	DOMAIN:	0.50
-THNMQ---	273	0.41	544	DOMAIN:	0.50
--ANMQR--	144	0.04	150		
-GANMQ---	145	0.04	148		
--FECLH--	129	0.07	136		
-YFECL---	139	0.04	144		
AYFEC----	151	0.11	154		
--GWMHD--	123	0.83	124		
--GSYHD--	200	0.02	203		
--WMSHG--	161	0.55	322	DOMAIN:	0.50
-VWMSH---	183	0.63	362	DOMAIN:	0.51
--PTYNF--	149	0.04	153		
---TYNFP-	196	0.04	202		
--AMLCY--	145	0.14	149		
---MLCYV-	156	0.13	159		
--GYDHI--	218	0.06	220		
-PGYDH---	162	0.09	166		
--LYPAM--	160	0	172		
---YPAME-	152	0.03	155		
--HIDHG--	350	0.18	703	NP_BIND:	0.50
-AHIDH---	337	0.10	671	NP_BIND:	0.49
--IDWQG--	153	0.26	297	DOMAIN:	0.47
---DWQGA-	137	0.09	268	DOMAIN:	0.47
--FENRF--	182	0.01	184		
---ENRFM-	143	0.06	145		
-PTDMQ---	152	0.05	166		
-FPDPW---	132	0.16	142		
-CDKIT---	133	0.02	261	METAL:	0.48
-CGRYE---	124	0.06	255	BINDING:	0.49
-HRFAI---	197	0.03	199		
-GMIFQ---	230	0.06	456	DOMAIN:	0.49
-ARDTK---	170	0	177		
-PQFYF---	129	0.07	135		
-IPAWY---	139	0.20	142		
-IPCLD---	173	0	350	ACT_SITE:	0.49
-QMHAN---	178	0.32	180		
-YHDVD---	235	0.05	238		
-YQTVY---	130	0.05	132		
-TDVWV---	131	0.05	138		
-IAMGH---	156	0.05	159		
-RDVHP---	147	0	155		
-MGAQM---	224	0.08	233		
-MNPMD---	210	0.35	213		
-MNIGQ---	185	0.06	189		
-DHRIN---	195	0.06	201		
-FHIGG---	124	0	135		
-AFMPW---	144	0.47	146		
-VMFGN---	131	0.02	138		
-IWTTT---	175	0.01	187		
-YAEGY---	270	0	270		
-PYQHG---	131	0.09	139	REGION:	0.91
-ATIEW---	132	0.05	136		
-PHYTR---	124	0.09	125		
-ESHNH---	149	0.04	157		
-VMPQT---	223	0.03	410	REGION:	0.44
-MMVGR---	136	0.11	273	DOMAIN:	0.48
-IENYS---	173	0	341	DOMAIN:	0.45
-YFYPD---	222	0.08	229		
-HGTYP---	155	0.03	158		
-KDYEC---	142	0.07	150		
-ITHEM---	195	0.09	383	DOMAIN:	0.49
-PYRKV---	158	0	159		
-PMFHQ---	126	0.45	134		
-RYYQI---	125	0.06	131		
-NSMNC---	124	0.26	128		
-GPMTI---	183	0.01	184		
