# Supplementary material for: Characterization of oligopeptide patterns in large protein sets
Source: BMC Genomics. 2007 Oct 1;8:346. doi: 10.1186/1471-2164-8-346 (PMC2231379; doi:10.1186/1471-2164-8-346)
Supplement: Additional file 4 — Top 100 peptides of each category in genome data set. Data table for peptide classes of the genome data set. As for Additional file 3, with additional 1–3 columns introduced between columns 4 and 5, showing the number of species in which the peptide pattern was found. [file 1471-2164-8-346-S4.pdf]

Genome

POP

A

--RFHPR-	43	0	20	A:17	B:67	E:29				
---FHPRC	47	0	16	A:17	B:66	E:23	DISULFID:	0.56		
--QFHPE-	122	0	2928	A:30	B:288	E:50	ACT_SITE:	0.50	DOMAIN:	0.26
-VQFHP--	82	0	1187	A:29	B:284	E:50	ACT_SITE:	0.33	DOMAIN:	0.32
--SGGQQ-	147	0	1284	A:30	B:290	E:45	DOMAIN:	0.47		
---GGEQQ	48	0	287	A:24	B:260	E:43	DOMAIN:	0.41		
--NFTDQ-	57	0	14	A:4	B:52	E:29	CARBOHYD:	0.21	TOPO_DOM:	0.21
---FTDQS	91	0	25	A:4	B:48	E:39				
--QFTDL-	61	0	65	A:10	B:89	E:40				
--PKCGS-	62	0	59	A:24	B:48	E:35				
--PKCGG-	52	0	94	A:22	B:58	E:37				
--PVCGS-	44	0	252	A:23	B:144	E:43	DISULFID:	0.38		
--TCPYC-	50	0	125	A:26	B:106	E:33	METAL:	0.22		
--RCPEC-	48	0	75	A:17	B:69	E:40	ZN_FING:	0.28	METAL:	0.21
---CPRCE	52	0	80	A:27	B:46	E:38				
--DIGCG-	50	0	84	A:17	B:233	E:49				
--CIGCG-	81	0	155	A:26	B:200	E:47	METAL:	0.62		
-KCIGC--	55	0	121	A:25	B:136	E:28	METAL:	0.64		
--QRQRV-	74	0	239	A:23	B:252	E:45	DOMAIN:	0.39		
--QQQRV-	98	0	543	A:28	B:263	E:43	DOMAIN:	0.46		
--GQRQR-	109	0	643	A:23	B:286	E:46	DOMAIN:	0.45		
--GQQQR-	123	0	1254	A:30	B:289	E:45	DOMAIN:	0.48		
--CGKST-	91	0	1606	A:27	B:280	E:50	NP_BIND:	0.32	DOMAIN:	0.31
--CGKTT-	100	0	563	A:25	B:253	E:50	NP_BIND:	0.35	DOMAIN:	0.25
TGCGK---	44	0	230	A:19	B:176	E:50	NP_BIND:	0.37		
--PGNYT-	54	0	81	A:20	B:76	E:38	CARBOHYD:	0.21		
--PGNHD-	58	0	31	A:28	B:182	E:40	TOPO_DOM:	0.23		
--QNPVH-	113	0	14	A:4	B:15	E:23				
--QNPTH-	52	0	6	A:5	B:18	E:29				
VFQNP---	49	0	185	A:25	B:180	E:31	DOMAIN:	0.45		
--CVGCG-	69	0	81	A:21	B:104	E:35	METAL:	0.27		
--CTGCG-	89	0	133	A:21	B:152	E:38	METAL:	0.36		
--CPRCG-	176	0	278	A:29	B:178	E:45	METAL:	0.27		
--CRRCG-	59	0	175	A:29	B:93	E:47	METAL:	0.48	ZN_FING:	0.30
--CDSCG-	49	0	70	A:9	B:89	E:42	ZN_FING:	0.26		
--CPECG-	188	0	427	A:27	B:224	E:45	ZN_FING:	0.33		
--CPHCG-	96	0	128	A:28	B:195	E:42				
--CPGCG-	73	0	153	A:30	B:177	E:41				
--CPSCG-	80	0	196	A:25	B:210	E:48				
--CPNCG-	149	0	186	A:30	B:166	E:45				
--CPICG-	86	0	188	A:25	B:153	E:47	ZN_FING:	0.24	METAL:	0.22
--CPYCG-	130	0	155	A:31	B:151	E:39	METAL:	0.31		
--CPDCG-	71	0	227	A:22	B:171	E:41	ZN_FING:	0.26		
--CPKCG-	273	0	317	A:30	B:217	E:48				
--CPACG-	82	0	158	A:23	B:177	E:40				
--CGLCV-	44	0	78	A:19	B:99	E:45	METAL:	0.28		
--CGACV-	58	0	76	A:25	B:145	E:34	METAL:	0.47		
--CGICA-	61	0	75	A:23	B:47	E:24	METAL:	0.45		
--GCGIC-	96	0	63	A:26	B:59	E:48	METAL:	0.44		
--GCGVC-	47	0	65	A:21	B:84	E:32				
--GCGAC-	46	0	213	A:22	B:137	E:38	METAL:	0.38		
--GMGGM-	61	0	403	A:17	B:245	E:49				
--GMDKM-	59	0	54	A:31	B:21	E:44				
--KCPVC-	65	0	115	A:23	B:50	E:46	ZN_FING:	0.23		
--VCPVC-	62	0	135	A:27	B:97	E:40	ZN_FING:	0.22		
---CPVCG	258	0	369	A:31	B:216	E:46				
--RCPVC-	47	0	89	A:19	B:89	E:41				
--CPVDA-	73	0	211	A:22	B:179	E:42	METAL:	0.46		
--CPVNA-	54	0	50	A:21	B:71	E:29	METAL:	0.46		
--CIKCG-	45	0	64	A:20	B:44	E:37	METAL:	0.31		
--CKKCG-	76	0	160	A:23	B:57	E:43	ZN_FING:	0.26		
--CRKCG-	52	0	95	A:24	B:47	E:44	ZN_FING:	0.28		
--CSKCG-	50	0	133	A:22	B:61	E:45	ZN_FING:	0.35		
-KCPKC--	85	0	170	A:25	B:82	E:46	ZN_FING:	0.24		
--GHVDH-	95	0	1247	A:31	B:303	E:46	NP_BIND:	0.39		
---HVDHG	99	0	1390	A:31	B:303	E:51	NP_BIND:	0.40		
--TTHYM-	45	0	29	A:25	B:62	E:28	DOMAIN:	0.34	VAR_SEQ:	0.31
-LTTHY--	54	0	44	A:26	B:182	E:37	DOMAIN:	0.25		
--KCSNC-	43	0	72	A:10	B:55	E:42	METAL:	0.22		
---CSNCG	52	0	119	A:18	B:136	E:47				
--WAWDF-	63	0	8	A:4	B:10	E:7	TOPO_DOM:	0.50		
--WLWDF-	45	0	15	A:4	B:10	E:15	REGION:	0.33		
--WNFGD-	82	0	32	A:11	B:45	E:20	TOPO_DOM:	0.31		
--WTFGD-	48	0	11	A:9	B:40	E:21	REGION:	0.27		
--WDFGD-	303	0	27	A:12	B:49	E:29	DOMAIN:	0.44	TOPO_DOM:	0.26
--IHHRI-	69	0	5	A:5	B:40	E:29				
-EIHHR--	80	0	28	A:7	B:85	E:31				
--IYMYD-	95	0	2	A:9	B:9	E:16				
-DIYMY--	93	0	20	A:5	B:16	E:21				
--STEQN-	142	0	42	A:6	B:55	E:42				
--LDVGC-	49	0	194	A:21	B:216	E:50				
--WWDYG-	45	0	16	A:25	B:9	E:47	MUTAGEN:	0.38	TOPO_DOM:	0.25
--PGCPP-	51	0	325	A:26	B:200	E:39	DISULFID:	0.31	METAL:	0.23
--MYDLS-	78	0	13	A:7	B:27	E:29				
--HAHLD-	49	0	38	A:31	B:121	E:41				
--CLGHQ-	51	0	475	A:26	B:260	E:39	DOMAIN:	0.34	ACT_SITE:	0.33
--HGPPG-	53	0	180	A:18	B:67	E:49	NP_BIND:	0.23		
--CGYEN-	44	0	11	A:15	B:20	E:16				
--PHMTV-	47	0	48	A:17	B:189	E:17	DOMAIN:	0.48		
--GMKQR-	51	0	39	A:25	B:166	E:29	DOMAIN:	0.33		
--FRESQ-	51	0	33	A:6	B:37	E:36	REGION:	0.30		
--LAGCS-	63	0	189	A:9	B:212	E:43	LIPID:	0.48	SIGNAL:	0.26
--MTGWR-	60	0	35	A:26	B:221	E:11				
--PEQFP-	58	0	106	A:31	B:58	E:31	REPEAT:	0.37		
--VTHDQ-	69	0	349	A:20	B:245	E:31	DOMAIN:	0.47		
--QAQIL-	57	0	100	A:21	B:235	E:44				
--PVHTY-	103	0	21	A:7	B:51	E:30				
--HGKTT-	86	0	1139	A:31	B:303	E:48	NP_BIND:	0.44		
--PPTPE-	44	0	171	A:19	B:170	E:47				
--HTPGH-	53	0	15	A:27	B:193	E:31	METAL:	0.40		

B

-CTHCG-	97	0	50	A:8	B:71	E:35	ZN_FING:	0.30						
RCIHC--	84	0	20	A:5	B:74	E:35	METAL:	0.40						
-CVHCK-	109	0	28	A:1	B:84	E:29	METAL:	0.50						
NCVHC--	93	0	22	A:6	B:78	E:24	METAL:	0.64						
-WWNPD-	90	0	5	A:3	B:83	E:1								
-WWFWD-	117	0	15	A:0	B:87	E:1	TRANSMEM:	0.27						
-MMVWC-	88	0	2	A:0	B:5	E:1								
-NQVWC-	115	0	5	A:0	B:17	E:2								
-GCPFC-	81	0	30	A:5	B:66	E:31	ZN_FING:	0.27						
-RCPFC-	172	0	170	A:13	B:134	E:34	METAL:	0.37						
--CPNCK	103	0	71	A:8	B:65	E:47	ZN_FING:	0.21						
-PCPMC-	138	0	10	A:8	B:118	E:14	METAL:	0.40						
-CPKCN-	102	0	155	A:18	B:85	E:40	ZN_FING:	0.23						
ACPYC--	85	0	58	A:5	B:67	E:22	METAL:	0.52						
-QCPHC-	110	0	34	A:3	B:75	E:32	ZN_FING:	0.24						
--CPHCH	111	0	75	A:3	B:73	E:22	METAL:	0.48						
--CPHCK	118	0	59	A:7	B:79	E:40	METAL:	0.24						
-ACPHC-	118	0	63	A:7	B:81	E:29	ZN_FING:	0.29	METAL:	0.25				
-YCPHC-	135	0	58	A:8	B:102	E:32	DISULFID:	0.28						
-PCPHC-	81	0	24	A:1	B:61	E:34	ZN_FING:	0.38						
--CPHCQ	124	0	95	A:6	B:88	E:40	ZN_FING:	0.28						
--CPQCQ	88	0	83	A:3	B:62	E:35	ZN_FING:	0.24						
-HCPFC-	112	0	75	A:4	B:86	E:26								
-HCPYC-	81	0	17	A:1	B:62	E:21								
-NICRC-	109	0	10	A:5	B:67	E:21								
-NPCPC-	123	0	3	A:0	B:121	E:13								
-CCHNP-	145	0	16	A:0	B:96	E:6	STRAND:	0.25						
-HCHND-	111	0	54	A:21	B:99	E:16								
-CPTCH-	154	0	280	A:6	B:111	E:37	METAL:	0.45	ZN_FING:	0.24	REPEAT:	0.23		
-CPKCH-	84	0	99	A:2	B:66	E:38	METAL:	0.28						
-CIGCH-	198	0	61	A:7	B:111	E:10	METAL:	0.43						
-CDVCH-	86	0	137	A:0	B:75	E:33	METAL:	0.32	ZN_FING:	0.26				
-CEVCH-	92	0	89	A:1	B:81	E:35	ZN_FING:	0.34	DOMAIN:	0.26				
-CGTCH-	107	0	89	A:1	B:67	E:21	METAL:	0.35						
-CESCH-	100	0	19	A:2	B:51	E:25								
-CGSCH-	162	0	42	A:3	B:81	E:18	BINDING:	0.33	METAL:	0.31				
-CLNCH-	80	0	13	A:0	B:54	E:27	BINDING:	0.31						
-CSNCH-	87	0	52	A:4	B:62	E:30	METAL:	0.31						
-CMACH-	91	0	16	A:2	B:53	E:8	BINDING:	0.50	METAL:	0.25				
-CQTCH-	89	0	69	A:3	B:54	E:12	METAL:	0.33						
-CQSCH-	87	0	17	A:2	B:50	E:22	BINDING:	0.24	METAL:	0.24				
-CSDCH-	95	0	39	A:2	B:52	E:20	BINDING:	0.41	METAL:	0.21				
-CPCCG-	93	0	30	A:4	B:55	E:30								
FCPCH--	91	0	105	A:0	B:86	E:22	METAL:	0.38						
-CSFCG-	296	0	367	A:15	B:258	E:42	ZN_FING:	0.48						
YCSFC--	175	0	147	A:0	B:147	E:23	ZN_FING:	0.44						
HCGFC--	83	0	4	A:5	B:77	E:9	METAL:	0.50						
-CEHHM-	81	0	57	A:2	B:74	E:8	DISULFID:	0.47						
-CEHCL-	90	0	15	A:2	B:68	E:26	METAL:	0.40						
-GCICC-	195	0	5	A:8	B:115	E:20								
-GCFWC-	163	0	85	A:8	B:129	E:5	ACT_SITE:	0.49						
-PDWCI-	92	0	54	A:0	B:91	E:5								
IQDWC--	92	0	67	A:0	B:91	E:19								
-HCFGC-	203	0	68	A:1	B:167	E:13	ZN_FING:	0.49						
-KCTGC-	99	0	19	A:19	B:58	E:26	METAL:	0.32						
-QCTHY-	191	0	3	A:1	B:6	E:21								
--CTHYP	164	0	83	A:1	B:153	E:22								
-ACCPF-	91	0	39	A:0	B:85	E:10	ZN_FING:	0.49						
--CCPFH	98	0	39	A:0	B:96	E:5	ZN_FING:	0.49						
-QCGKC-	80	0	121	A:2	B:62	E:37	METAL:	0.40						
-QCGYC-	215	0	13	A:11	B:96	E:14	METAL:	0.46						
-QCGFC-	181	0	49	A:10	B:98	E:39								
-GWMHN-	92	0	20	A:0	B:89	E:4	REGION:	0.40						
-YWMHN-	86	0	84	A:0	B:84	E:1								
--WMHNG	93	0	90	A:0	B:92	E:10								
-WCPPC-	123	0	33	A:2	B:90	E:32	DISULFID:	0.33	DOMAIN:	0.27				
-WCGPC-	599	0	684	A:12	B:275	E:49	DOMAIN:	0.23	DISULFID:	0.23	ACT_SITE:	0.21	SITE:	0.21
-CSNMC-	85	0	10	A:1	B:66	E:7								
--SNMCH	96	0	11	A:1	B:74	E:11								
-HCWRH-	83	0	58	A:0	B:80	E:4								
--CWRHK	93	0	74	A:0	B:91	E:8								
-YPHCW-	145	0	104	A:8	B:143	E:7								
--PHCWR	146	0	104	A:8	B:145	E:5								
-THMCY-	130	0	229	A:1	B:128	E:6	METAL:	0.66						
HTHMC--	132	0	229	A:1	B:130	E:8	METAL:	0.66						
-CTTNC-	396	0	693	A:2	B:273	E:47	REGION:	0.34	ACT_SITE:	0.33				
-CMGNQ-	115	0	18	A:2	B:111	E:14	METAL:	0.22						
-FYHCF-	117	0	30	A:2	B:110	E:10	ZN_FING:	0.43						
-FCDWY-	140	0	114	A:0	B:138	E:2								
-PFHMW-	105	0	15	A:5	B:95	E:1								
-WCIYP-	92	0	47	A:0	B:91	E:30								
-CEWKN-	105	0	2	A:0	B:5	E:5								
-CNQAC-	90	0	30	A:2	B:76	E:22	TOPO_DOM:	0.23						
-HCKTC-	128	0	49	A:1	B:110	E:42	METAL:	0.37						
-QMHHH-	92	0	21	A:0	B:70	E:16	VAR_SEQ:	0.29						
-WHMYD-	83	0	14	A:0	B:81	E:26								
-HHCMR-	85	0	5	A:1	B:61	E:27								
-WCRGY-	116	0	5	A:0	B:14	E:23								
-WASWC-	212	0	36	A:0	B:131	E:6	DISULFID:	0.31	DOMAIN:	0.22				
-CCGQP-	111	0	9	A:1	B:90	E:11	METAL:	0.22						
-MVCHH-	100	0	203	A:3	B:96	E:12	ACT_SITE:	0.47						
-HPHWC-	94	0	1	A:0	B:8	E:3								
-WNCKY-	129	0	2	A:1	B:15	E:8								
-CACCS-	103	0	75	A:1	B:99	E:43	METAL:	0.64						
-AWYWH-	81	0	134	A:0	B:77	E:13								
-HFCSM-	187	0	118	A:0	B:153	E:9								
-NCWDN-	218	0	1	A:0	B:41	E:4								
-WSEHC-	125	0	111	A:20	B:120	E:8								
-WHIEC-	248	0	501	A:19	B:240	E:49	METAL:	0.50						
-FHPTC-	81	0	45	A:0	B:75	E:28	DISULFID:	0.29						

E

--PCGWW--	53	0	9	A:0	B:4	E:21						
--WQGWW--	48	0	6	A:0	B:13	E:24	REPEAT:	0.33				
--WMMIH--	44	0	8	A:0	B:4	E:15	DOMAIN:	0.38				
CNWMM----	68	0	9	A:0	B:2	E:17	DOMAIN:	0.44				
--WMMWA--	54	0	11	A:0	B:2	E:22	TRANSMEM:	0.36	TOPO_DOM:	0.27		
--MAMYW--	77	0	15	A:0	B:9	E:18						
----MYWQH	46	0	6	A:0	B:3	E:16	DOMAIN:	0.33				
--MFHWC--	61	0	25	A:0	B:1	E:20	TOPO_DOM:	0.44				
---FHWCC-	283	0	108	A:0	B:0	E:29						
--RCHWC--	58	0	12	A:0	B:6	E:20	TOPO_DOM:	0.50	VAR_SEQ:	0.33		
--YHYHC--	46	0	8	A:0	B:3	E:19	ZN_FING:	0.50				
----YHCNW	63	0	13	A:0	B:0	E:20	ZN_FING:	0.31	TOPO_DOM:	0.31		
--WWFCA--	69	0	49	A:0	B:1	E:17	TRANSMEM:	0.49				
--WWKCK--	59	0	7	A:0	B:0	E:23						
--YCMWR--	56	0	8	A:0	B:0	E:22	DOMAIN:	0.50				
--ECMWC--	56	0	4	A:0	B:0	E:18	TOPO_DOM:	0.75	COMPBIAS:	0.25		
--QCMWF--	45	0	12	A:0	B:0	E:22	REPEAT:	0.42				
-MQCMW---	56	0	12	A:0	B:0	E:28	REPEAT:	0.42				
--YWTMH--	120	0	1	A:0	B:0	E:3						
--YWWMN--	64	0	16	A:0	B:5	E:19	TOPO_DOM:	0.44				
--HQWNW--	74	0	10	A:0	B:1	E:3	TRANSMEM:	0.30	TOPO_DOM:	0.30		
-HHRWN---	53	0	9	A:0	B:3	E:22	CARBOHYD:	0.33				
--HWCEY--	43	0	16	A:0	B:0	E:19	ZN_FING:	0.44				
--HWWEY--	49	0	6	A:1	B:1	E:21						
--HWQTY--	63	0	0	A:0	B:9	E:24						
--YQHHW--	47	0	5	A:0	B:4	E:21	TOPO_DOM:	0.80				
-WYQYH---	73	0	33	A:0	B:4	E:14						
--WNNHQ--	83	0	21	A:0	B:6	E:13	DOMAIN:	0.24				
--WWTHQ--	45	0	5	A:0	B:5	E:27						
--WMQKW--	47	0	8	A:0	B:4	E:17	BINDING:	0.25	TOPO_DOM:	0.25		
--WMMYW--	82	0	39	A:0	B:2	E:24	TRANSMEM:	0.38				
--WMGTW--	85	0	54	A:0	B:1	E:21	DOMAIN:	0.46				
--WYWCT--	107	0	18	A:0	B:2	E:20	TRANSMEM:	0.44				
-GWYWC---	71	0	24	A:0	B:0	E:16	TOPO_DOM:	0.33	DOMAIN:	0.21	DISULFID:	0.21
-IWMWC---	97	0	12	A:0	B:0	E:14						
--WCCYV--	207	0	43	A:0	B:2	E:25						
-DWCCH---	49	0	27	A:0	B:3	E:18	DISULFID:	0.70				
--HNWWQ--	46	0	9	A:0	B:4	E:19	DOMAIN:	0.44				
--HNMWH--	78	0	19	A:0	B:3	E:24	TOPO_DOM:	0.47				
--WQYRW--	57	0	2	A:0	B:6	E:27	DOMAIN:	0.50				
----YRWWW	62	0	6	A:0	B:8	E:27						
----WRWRW	47	0	0	A:0	B:18	E:6						
--WYHMY--	56	0	3	A:0	B:0	E:14	DOMAIN:	0.33	TOPO_DOM:	0.33		
---YPMYW-	43	0	4	A:0	B:4	E:15	DOMAIN:	0.50				
--NWMHY--	83	0	42	A:0	B:1	E:26	DOMAIN:	0.48				
---WHHYW-	43	0	16	A:0	B:1	E:20	TOPO_DOM:	0.50				
--SWIMW--	51	0	25	A:0	B:6	E:18	TRANSMEM:	0.48				
--MWIMM--	46	0	3	A:0	B:2	E:30						
--WCWYC--	47	0	2	A:0	B:0	E:27	ZN_FING:	0.50				
QAWCW----	66	0	57	A:0	B:10	E:20	REGION:	0.28	DISULFID:	0.28	TOPO_DOM:	0.28
--HSWWC--	76	0	7	A:0	B:6	E:24						
---KWWCR-	50	0	3	A:0	B:1	E:19	DISULFID:	0.67	DOMAIN:	0.33	TOPO_DOM:	0.33
--DFWWC--	70	0	5	A:0	B:1	E:24						
--FWWCI--	70	0	5	A:0	B:1	E:24						
---WWCID-	58	0	5	A:0	B:0	E:22						
--WWDHF--	569	0	0	A:1	B:0	E:3						
MSWWD----	101	0	12	A:9	B:6	E:38	TOPO_DOM:	0.25				
--EWWWD--	53	0	4	A:0	B:1	E:22	DOMAIN:	0.25				
---WWWDC-	46	0	2	A:0	B:0	E:16						
--WYGWH--	97	0	37	A:1	B:12	E:46	TRANSMEM:	0.24	TOPO_DOM:	0.24		
--WYYWK--	53	0	8	A:0	B:0	E:17	DOMAIN:	0.50				
--HHHWD--	52	0	21	A:0	B:9	E:28	METAL:	0.71				
-AHMHW---	49	0	4	A:0	B:2	E:19						
--HRWCM--	49	0	0	A:0	B:1	E:1						
----WCMRH	313	0	0	A:0	B:1	E:13						
--WMWNQ--	55	0	16	A:0	B:1	E:18	DOMAIN:	0.56	TOPO_DOM:	0.56		
--WMFMQ--	125	0	0	A:0	B:0	E:1						
--EWFWW--	66	0	14	A:0	B:2	E:29						
-AEWYW---	96	0	31	A:0	B:4	E:23	DOMAIN:	0.42				
--WDWHA--	50	0	5	A:0	B:9	E:8						
-FWDWH---	43	0	0	A:0	B:4	E:2						
--GDWHW--	43	0	0	A:0	B:6	E:17						
--YCWCW--	47	0	7	A:0	B:0	E:18	TOPO_DOM:	0.43	VAR_SEQ:	0.29		
-NYCWC---	44	0	7	A:0	B:0	E:16	TOPO_DOM:	0.43	VAR_SEQ:	0.29		
--KWWYD--	140	0	27	A:0	B:2	E:21						
--KWWDD--	148	0	5	A:4	B:19	E:9						
--HWPWM--	110	0	19	A:0	B:1	E:17						
-KHWPW---	108	0	19	A:1	B:0	E:18						
--YWDWT--	128	0	23	A:0	B:17	E:10	REGION:	0.43				
--HWVYW--	72	0	115	A:0	B:6	E:23	TRANSMEM:	0.44				
--NPWHC--	85	0	8	A:0	B:4	E:26	TOPO_DOM:	0.25				
--HMMRC--	60	0	29	A:0	B:3	E:17	DISULFID:	0.41	DOMAIN:	0.21		
--WYHTC--	43	0	14	A:0	B:0	E:10	DOMAIN:	0.50	DISULFID:	0.50		
--WEFMW--	56	0	12	A:2	B:1	E:19						
--PWANC--	49	0	25	A:1	B:2	E:20	REPEAT:	0.32				
--KHMWP--	100	0	42	A:1	B:7	E:27	METAL:	0.43				
--FCWMC--	62	0	8	A:0	B:0	E:31	ZN_FING:	0.50				
--MWDCM--	198	0	35	A:0	B:0	E:23	REPEAT:	0.40				
--WYCNR--	58	0	7	A:0	B:8	E:27						
--WWYAH--	79	0	5	A:0	B:2	E:25						
--WWWVP--	49	0	22	A:0	B:9	E:20	TOPO_DOM:	0.32	TRANSMEM:	0.27		
--RYYHW--	43	0	10	A:0	B:9	E:17	TOPO_DOM:	0.50				
--MDWVW--	51	0	4	A:0	B:6	E:18	TRANSMEM:	0.25				
--WWENY--	98	0	0	A:0	B:7	E:8						
--WNQHY--	49	0	5	A:0	B:5	E:21	DOMAIN:	0.80				
--MPWFH--	75	0	29	A:0	B:3	E:22	DOMAIN:	0.41				
--YCKWH--	203	0	1	A:0	B:0	E:3						
--CDQYW--	180	0	43	A:0	B:1	E:24	DOMAIN:	0.35	TOPO_DOM:	0.30		
--MHNWY--	46	0	6	A:0	B:1	E:19	ZN_FING:	0.50				
--WHRWQ--	52	0	21	A:0	B:9	E:16	TOPO_DOM:	0.48				

Genome

NEP

A

--KNGPE--	23	0.01	48	B:53	E:36		
--GNGLE--	25	0	133	B:134	E:46		
--PVKGP--	23	0.01	27	B:64	E:40		
--PVKTK--	23	0	111	B:86	E:44		
--VEAHT--	26	0.03	53	B:76	E:30		
--AEAHL--	24	0	64	B:160	E:40		
--SEGPG--	27	0	89	B:81	E:43		
---EGPGD-	25	0	55	B:85	E:38		
-KDEGP---	22	0	52	B:57	E:40	PEPTIDE:	0.23
--AEGPS--	22	0	73	B:104	E:41		
--LEGPS--	30	0	104	B:109	E:40		
--AKEPI--	23	0	68	B:181	E:44		
-VAREP---	24	0	37	B:117	E:34		
--VFIRI--	24	0	30	B:47	E:36		
---FLRIN-	23	0	70	B:114	E:40		
--RAEML--	25	0	138	B:174	E:39		
----EMLVI	28	0	22	B:77	E:31		
--AGANN--	24	0.02	14	B:96	E:34		
---GLNNG-	24	0	43	B:96	E:44		
---GANTG-	22	0	60	B:128	E:44		
--GAERK--	27	0	124	B:139	E:45		
---AEYKN-	22	0.01	24	B:65	E:43		
--AIFFR--	22	0.02	231	B:55	E:27	TRANSMEM:	0.46
--IIFFK--	24	0	19	B:57	E:40		
--AKPKL--	23	0	100	B:121	E:45		
-AAKPT---	24	0	79	B:151	E:42		
--AIQVV--	26	0	110	B:114	E:40		
--ALQVR--	24	0	89	B:172	E:44		
--NEEGG--	26	0	37	B:106	E:49		
-NNEEG---	23	0	37	B:61	E:43		
-DNEGG---	22	0	24	B:57	E:40		
--NSAEG--	23	0	85	B:104	E:40	TOPO_DOM:	0.26
--RPAEG--	25	0	70	B:126	E:41		
--LGQKE--	22	0	85	B:92	E:41		
---GQKEE-	26	0	78	B:64	E:42		
---GGKED-	29	0	68	B:80	E:45		
--KLMII--	23	0	19	B:48	E:27	TRANSMEM:	0.21
-GDLMI---	22	0	49	B:87	E:40		
--KGTDA--	23	0	54	B:115	E:41		
TAKGT----	24	0	84	B:148	E:44		
--GAANE--	32	0	49	B:122	E:43		
--GDANL--	23	0	68	B:157	E:43		
--LVMEK--	22	0	39	B:107	E:43		
LKLVM----	25	0	47	B:59	E:41		
--ELIMS--	22	0	58	B:54	E:41		
-IEIIM---	22	0	12	B:42	E:21		
--EGGPK--	28	0	35	B:40	E:38		
-TTGGP---	22	0	47	B:92	E:37		
--IVIQL--	26	0	106	B:93	E:40	TRANSMEM:	0.38
-AIVLQ---	26	0	67	B:153	E:47		
--LEDQA--	26	0	142	B:144	E:44		
---EDQEA-	27	0	95	B:92	E:48		
--GLGPP--	23	0	126	B:49	E:40		
--VLGPP--	26	0	62	B:89	E:46		
--RGTKL--	24	0	46	B:106	E:47		
--RGPKL--	22	0	50	B:70	E:33		
--FKLSI--	28	0	31	B:91	E:45		
--FRLSI--	23	0	29	B:87	E:41		
--RSVGK--	23	0	93	B:130	E:46		
--FIPIG--	25	0	44	B:90	E:42		
--VIIQV--	25	0	113	B:107	E:36	TRANSMEM:	0.36
--NDKIG--	23	0	33	B:83	E:31		
--SDPGE--	26	0	34	B:86	E:42		
--PTDLG--	25	0	60	B:135	E:40		
--KVNLF--	24	0	41	B:86	E:41		
--RAFII--	25	0	20	B:76	E:32		
--HLVAV--	23	0	64	B:131	E:39		
--ILRAP--	31	0	80	B:136	E:43	DOMAIN:	0.25
--AGEGM--	26	0	51	B:112	E:31		
--AYERP--	22	0.01	13	B:51	E:33		
--ASNKG--	29	0	64	B:83	E:47		
--FKLVT--	24	0	73	B:141	E:39		
--RKSKR--	22	0	131	B:96	E:47		
--TYGAK--	24	0.01	27	B:67	E:33		
--SEPER--	22	0	74	B:131	E:45		
--IEIYV--	24	0	37	B:56	E:35	REGION:	0.27
--DVVPK--	22	0	210	B:80	E:48		
--DDDKG--	27	0	76	B:66	E:44		
--ELVVM--	22	0	54	B:119	E:32		
--VILKM--	24	0	52	B:53	E:43		
--TKSEG--	28	0	61	B:62	E:43		
--GPTSA--	29	0	77	B:121	E:49		
--IFIEF--	22	0	16	B:48	E:24		
--KLFLM--	22	0	35	B:69	E:40		
--KYVRL--	26	0	53	B:73	E:43	DOMAIN:	0.25
--FVIKI--	32	0	22	B:33	E:38	DOMAIN:	0.23
--LLIMR--	25	0	21	B:87	E:36	TOPO_DOM:	0.29
--RTDSA--	25	0	40	B:145	E:45		
--RLMVK--	22	0	70	B:78	E:39	DOMAIN:	0.24
--GDMAA--	22	0	36	B:128	E:41		
--IYFEL--	23	0	14	B:38	E:36	TOPO_DOM:	0.21
--ETNKI--	24	0	89	B:130	E:43		
--KGGAP--	24	0	61	B:122	E:42		
--LAFYE--	25	0	48	B:129	E:40		
--RGATF--	23	0	27	B:97	E:34		
--LWLEL--	25	0.01	33	B:111	E:39		
--KTTKV--	24	0	75	B:76	E:49		
--EEEDM--	22	0	80	B:40	E:47		
--LEIEM--	27	0	39	B:60	E:36		
--FKGAV--	24	0	34	B:102	E:36		

B

--RAMWF-	29	0	2	A:0	E:5	TOPO_DOM:	0.50				
--RAHMF-	36	0	5	A:0	E:11						
--EPTEC-	33	0	27	A:0	E:29	REGION:	0.22	DISULFID:	0.22		
--QETEC-	30	0	4	A:2	E:23	STRAND:	0.25				
--GYMFP-	34	0	7	A:0	E:18						
--LYMYP-	34	0	0	A:0	E:13						
--WYITV-	36	0	4	A:0	E:12	DOMAIN:	0.50				
--MYHTV-	30	0	2	A:1	E:13	TRANSMEM:	0.50				
--IMFQF-	36	0	0	A:0	E:10						
--IMMEF-	32	0	8	A:2	E:24						
--CIIIQ-	30	0	4	A:3	E:20	DOMAIN:	0.25				
QICII---	36	0	0	A:1	E:20						
--HMYAE-	30	0	0	A:2	E:4						
--AMYME-	35	0	1	A:1	E:11						
--PCKAK-	30	0	22	A:3	E:17	VAR_SEQ:	0.23				
RAPCK---	40	0	4	A:2	E:17	DOMAIN:	0.25	REPEAT:	0.25		
--RGPMW-	29	0	2	A:0	E:6	DOMAIN:	0.50				
--RGPPW-	72	0	7	A:0	E:21	VAR_SEQ:	0.29				
KCRGP---	29	0	1	A:1	E:23						
-IRGPC--	29	0	4	A:2	E:19						
--VLKMW-	36	0	7	A:1	E:21	DOMAIN:	0.43				
--ALYMW-	37	0	1	A:3	E:4						
---LIMWD	29	0	3	A:0	E:13	DOMAIN:	0.33				
--HLIIW-	35	0	8	A:2	E:13	DISULFID:	0.50	TOPO_DOM:	0.25		
---LYIWP	38	0	16	A:0	E:17	TRANSMEM:	0.38				
--EDNEC-	30	0	11	A:0	E:25	DISULFID:	0.27				
---DNKCL	35	0	16	A:0	E:35						
--QNSMY-	30	0	2	A:0	E:22						
--KFSMY-	37	0	3	A:1	E:12	REPEAT:	0.33				
--GMFMK-	31	0	8	A:2	E:16						
--HMFIK-	33	0	4	A:0	E:17	DOMAIN:	0.50	TOPO_DOM:	0.25		
--KMLYM-	29	0	1	A:2	E:11						
-PKMGY--	36	0	14	A:2	E:19	DOMAIN:	0.36				
--YMFEE-	40	0	7	A:2	E:31						
--LMFWE-	30	0	4	A:0	E:7	DOMAIN:	0.25	TRANSMEM:	0.25		
--KCPRI-	29	0	23	A:1	E:29	TOPO_DOM:	0.26				
--TCPRE-	32	0	13	A:1	E:26						
--LMCVK-	31	0	0	A:0	E:6						
--IMHVK-	38	0	5	A:1	E:21						
--LHMFQ-	31	0	3	A:0	E:20						
--LHMYV-	39	0	1	A:1	E:22						
--CKPPA-	29	0	92	A:0	E:25	DISULFID:	0.47	DOMAIN:	0.22		
--CVPPK-	33	0	12	A:1	E:23						
--QDPPM-	35	0	6	A:3	E:18						
-NKDPP--	53	0	14	A:2	E:33	REPEAT:	0.29				
-IWDPP--	35	0	3	A:1	E:11	COMPBIAS:	0.33				
--GPKKC-	31	0	20	A:0	E:28	DISULFID:	0.30				
--GPIKW-	33	0	13	A:0	E:19	DOMAIN:	0.31	DISULFID:	0.31	TOPO_DOM:	0.31
--GPPYK-	58	0	2	A:1	E:19						
-HGPPY--	48	0	1	A:0	E:15	TOPO_DOM:	1.00				
--GPPYM-	47	0	1	A:0	E:7						
--NHGPP-	31	0	10	A:0	E:26						
--NHDPP-	35	0	9	A:1	E:25	TOPO_DOM:	0.33	REPEAT:	0.22	VAR_SEQ:	0.22
--WPNTS-	34	0	10	A:1	E:25						
--DSHME-	30	0	4	A:1	E:24	VAR_SEQ:	0.25				
--HTAIW-	30	0	3	A:1	E:9						
--DQHNT-	39	0	0	A:1	E:15						
--PHMGQ-	30	0	3	A:0	E:14						
--ADCVW-	33	0	2	A:2	E:13	DOMAIN:	0.50				
--ASMCD-	31	0	20	A:0	E:22						
--YDLDC-	31	0	5	A:2	E:26	REGION:	0.40	ZN_FING:	0.40		
--LHLKC-	33	0	4	A:1	E:27	ZN_FING:	0.25	VAR_SEQ:	0.25		
--EVNMF-	42	0	37	A:0	E:27	DOMAIN:	0.46				
--HKMFL-	35	0	15	A:0	E:28						
--EFFYM-	31	0	0	A:3	E:7						
--WVQLW-	33	0	6	A:0	E:9						
--CTQDV-	31	0	6	A:1	E:21	COMPBIAS:	0.33				
--AKCML-	30	0	15	A:0	E:27						
--CKSER-	35	0	18	A:4	E:30	DISULFID:	0.22				
--PAKKC-	30	0	9	A:1	E:27	VAR_SEQ:	1.00	DOMAIN:	0.22		
--PCQEG-	32	0	36	A:0	E:24						
--YPINM-	30	0	14	A:5	E:19	TOPO_DOM:	0.29				
--RGDKC-	32	0	9	A:2	E:21						
--PACFR-	37	0	9	A:1	E:21						
--EETHM-	39	0	6	A:0	E:21	COMPBIAS:	0.33				
--EFWPI-	30	0	2	A:1	E:8						
--MWYLV-	33	0	7	A:2	E:11	TRANSMEM:	0.43				
--VVYHM-	30	0	3	A:2	E:17	TRANSMEM:	0.33				
--NMYVR-	29	0	3	A:2	E:16	DOMAIN:	0.33	VARIANT:	0.33	TOPO_DOM:	0.33
--CMPRG-	30	0	3	A:2	E:17	TOPO_DOM:	0.33				
--DVIMW-	29	0	0	A:1	E:10						
--RSKFW-	30	0	0	A:0	E:8						
--APMHK-	31	0	0	A:0	E:5						
--APQCN-	30	0	20	A:0	E:26	DISULFID:	0.45	TOPO_DOM:	0.25		
--IMEWG-	32	0	1	A:0	E:10	NoFeature:	1.00				
--CYDGR-	29	0	3	A:0	E:15	DOMAIN:	0.33	DISULFID:	0.33		
--WAFHP-	29	0	0	A:1	E:6						
--IMYIQ-	31	0	3	A:0	E:19	COILED:	0.33				
--LMWGQ-	31	0	0	A:0	E:6						
--RGCQK-	29	0	10	A:1	E:21	METAL:	0.30				
--NMKAH-	30	0	14	A:0	E:29	VAR_SEQ:	0.29	TOPO_DOM:	0.21		
--LKPPW-	33	0	7	A:1	E:17	REPEAT:	0.29	TOPO_DOM:	0.29		
--PRMCL-	30	0	16	A:1	E:23						
--KCAPK-	30	0	15	A:1	E:25	DOMAIN:	0.27				
--RHSCV-	31	0	4	A:0	E:24	DOMAIN:	0.25	DISULFID:	0.25	TOPO_DOM:	0.25
--YVIWD-	30	0	3	A:1	E:8	DOMAIN:	0.33				
--NAECD-	36	0	7	A:1	E:21	REPEAT:	0.29				
--VMWPR-	33	0	3	A:2	E:8						
--IYMHI-	30	0	39	A:0	E:12	METAL:	0.49				
--PRNDH-	38	0	2	A:0	E:18	TOPO_DOM:	0.50				

E

--NCCCA-	55	0	0	A:0	B:1				
-ANNCC--	41	0	3	A:0	B:4	MOTIF:	0.33	REGION:	0.33
--EWAYN-	35	0	2	A:2	B:4				
--RWAYK-	36	0	0	A:1	B:6				
--PCMGK-	39	0	3	A:0	B:0	DISULFID:	0.33	DOMAIN:	0.33
KGPCM---	38	0	6	A:0	B:2	DISULFID:	0.33		
-KPCMA--	45	0	0	A:0	B:7				
--WEFTG-	37	0	1	A:3	B:19				
-VWEFQ--	34	0	0	A:1	B:14				
--LEWSM-	61	0	3	A:0	B:3	ZN_FING:	0.33		
--ATWSM-	35	0	0	A:2	B:17				
--IWCAS-	34	0	6	A:0	B:12	DISULFID:	0.50		
--EWHAS-	37	0	2	A:2	B:22				
--IEWQA-	54	0	13	A:3	B:40	STRAND:	0.23		
--IKWQT-	33	0	4	A:0	B:16	REGION:	0.25		
--FFKWD-	39	0	1	A:0	B:12				
-QEFKW--	38	0	1	A:0	B:5	DOMAIN:	1.00		
--CCEFQ-	33	0	0	A:0	B:1				
-PCCEF--	34	0	0	A:0	B:0				
--CCEHK-	34	0	6	A:0	B:5	DOMAIN:	0.33		
--PCQFK-	38	0	9	A:0	B:4	DOMAIN:	0.44		
---CMFKV	34	0	0	A:0	B:1				
--WYAEP-	33	0	3	A:0	B:12				
-KWYAN--	35	0	10	A:2	B:22				
--PVCPW-	38	0	0	A:0	B:5				
-YPVCC--	36	0	1	A:0	B:6				
--LQWGY-	37	0	1	A:1	B:17				
--LQWGC-	35	0	2	A:0	B:14	DOMAIN:	0.50		
--ECICC-	36	0	2	A:0	B:1				
--ECMCL-	33	0	1	A:0	B:3				
--RCDLM-	50	0	1	A:0	B:8				
--NCDAM-	38	0	1	A:0	B:6				
--MCFKT-	36	0	0	A:0	B:3				
--MRFKY-	38	0	5	A:2	B:15				
--RWDFK-	35	0	1	A:2	B:22	REGION:	1.00		
--VWPFK-	38	0	0	A:0	B:6				
--AWYPE-	35	0	4	A:1	B:16	DOMAIN:	0.25		
---WYPGV	40	0	1	A:1	B:22				
--MCKFP-	36	0	0	A:0	B:1				
-IMCKF--	40	0	0	A:0	B:3				
--CCKQF-	33	0	3	A:1	B:2				
--CCKQV-	40	0	2	A:0	B:2				
--SNWHV-	37	0	1	A:1	B:15				
--VGCWP-	35	0	1	A:0	B:16				
--HKEWG-	35	0	14	A:0	B:18				
--HTHYR-	35	0	6	A:0	B:19				
--LTYWY-	35	0	2	A:1	B:24	PROPEP:	0.50		
--VAIYW-	38	0	2	A:3	B:29	TRANSMEM:	0.50		
--GWFIE-	34	0	9	A:2	B:52	TRANSMEM:	0.22		
--GPPWF-	48	0	0	A:1	B:13				
--VWYVE-	48	0	3	A:0	B:11	STRAND:	0.33		
--IACCD-	39	0	1	A:1	B:29				
--TNLCM-	41	0	1	A:0	B:1				
--YCPHE-	34	0	0	A:1	B:4				
--IWIIQ-	34	0	4	A:1	B:15	TRANSMEM:	0.50		
--KHTWK-	37	0	15	A:0	B:2	REGION:	0.47		
--KLMWP-	34	0	0	A:0	B:2				
--HFRCF-	37	0	2	A:0	B:11				
--MCRGT-	34	0	4	A:2	B:2	DOMAIN:	0.25	TOPO_DOM:	0.25
--VIWFE-	47	0	1	A:0	B:18				
--FFYMR-	38	0	23	A:4	B:7				
--IDPWP-	39	0	0	A:0	B:12				
--YRKWP-	34	0	17	A:0	B:4	DOMAIN:	0.24		
--EPMHN-	34	0	2	A:0	B:7				
--WSFEQ-	39	0	6	A:2	B:18				
--FGWQV-	36	0	3	A:0	B:45				
--EPWMS-	33	0	1	A:0	B:11				
--QMPYD-	37	0	3	A:0	B:11				
--HTSMH-	36	0	1	A:0	B:7				
--PAMYH-	41	0	1	A:0	B:13				
--LWPMK-	39	0	4	A:0	B:13	DISULFID:	0.25	DOMAIN:	0.25
--AWKVM-	33	0	8	A:1	B:20				
--FPYTM-	36	0	1	A:1	B:23				
--YTCCQ-	34	0	1	A:0	B:0	TOPO_DOM:	1.00		
--MRMVF-	34	0	2	A:3	B:14				
--QWIML-	33	0	1	A:0	B:6				
--YKFAM-	48	0	1	A:0	B:11				
--HEPKW-	33	0	0	A:0	B:5				
--CECYL-	44	0	0	A:0	B:2				
--HMKQD-	36	0	0	A:0	B:5				
--FEHCE-	37	0	4	A:0	B:19				
--CDTLW-	33	0	3	A:0	B:14	DOMAIN:	0.33		
--FLHVW-	36	0	2	A:2	B:18				
--MPNCA-	33	0	0	A:0	B:8				
--MRGHN-	38	0	6	A:3	B:23				
--MYLPC-	34	0	1	A:1	B:4				
--HEGCC-	35	0	7	A:0	B:16				
--CWGFS-	33	0	0	A:0	B:9				
--LMHIM-	35	0	2	A:0	B:10	TRANSMEM:	0.50		
--DMRHN-	34	0	2	A:2	B:7				
--TRHQC-	49	0	5	A:0	B:8	TOPO_DOM:	0.40		
--CESWT-	34	0	0	A:0	B:5				
--KCAWA-	37	0	1	A:2	B:4	TOPO_DOM:	1.00		
--YPWRK-	40	0	1	A:1	B:9	REGION:	1.00		
--GEHYY-	41	0	3	A:1	B:30				
--QYVMI-	49	0	6	A:5	B:16	STRAND:	0.33		
--WFMPL-	35	0	0	A:0	B:29				
--PIMRC-	35	0	2	A:0	B:3				
--YVHMR-	37	0	9	A:2	B:16				
--DEMPC-	34	0	0	A:0	B:6				

Genome

ORP

A

-MCCHY-	18	0	0
EMCCH--	18	0	0
--CCHYD	18	0	1
-WWHKN-	10	0	8
-WCPNA-	18	0	0
-CWYCF-	11	0	0

B

--HEWDG--	46	0	3	B:35						
--HNWPG--	72	0	0	B:52						
--ACMNH--	46	0	0	B:34						
--ACMHC--	55	0	12	B:47						
--FCDWY--	140	0	114	B:138						
--YCDWY--	57	0	34	B:57						
-AQCDW---	45	0	15	B:38	DOMAIN:	0.40				
--AMWWI--	54	0	8	B:49	TRANSMEM:	0.38				
-YAMWW---	45	0	2	B:42						
TYAMW----	49	0	2	B:46						
--WAMTQ--	71	0	1	B:16						
--WHMTL--	45	0	0	B:41						
--YPGWW--	47	0	1	B:39						
----GWWHW	50	0	6	B:42	STRAND:	0.33				
--YWQVY--	58	0	1	B:48						
-FGWQV---	55	0	3	B:45						
--GWLWF--	57	0	18	B:53						
--CWPWF--	48	0	4	B:45	DOMAIN:	0.25	TOPO_DOM:	0.25		
--WAWGH--	49	0	17	B:44	METAL:	0.35	CROSSLNK:	0.35	TRANSMEM:	0.24
--WCIGH--	45	0	8	B:40	STRAND:	0.25				
--WTMRW--	75	0	0	B:4						
--WTMAC--	45	0	24	B:44						
--FPMDW--	56	0	0	B:7						
---PMVWR-	54	0	7	B:43	REGION:	0.29				
--WHIWW--	61	0	11	B:53	TOPO_DOM:	0.27				
--WGGWW--	119	0	16	B:86	TRANSMEM:	0.25				
--MAHDW--	67	0	1	B:12						
---AHDWC-	60	0	1	B:6						
----HDWCH	55	0	0	B:1						
--WMPSW--	53	0	5	B:46						
--WWISW--	69	0	8	B:61	TRANSMEM:	0.50				
--QWWQA--	43	0	6	B:32						
--QWWYG--	102	0	6	B:88						
--YWAWW--	117	0	21	B:90	TRANSMEM:	0.48				
--AWANW--	46	0	2	B:41						
--GMNKW--	52	0	1	B:18	REGION:	1.00				
---MNAWA-	57	0	4	B:46						
--HWAYK--	78	0	16	B:76						
--WWAYY--	70	0	8	B:60	TRANSMEM:	0.50				
--HYNWH--	216	0	18	B:9	DOMAIN:	0.44				
--DYFWH--	46	0	2	B:41	HELIX:	0.50				
--TGWMW--	44	0	3	B:41	TOPO_DOM:	0.33				
----WMWPN	70	0	0	B:65						
--IFWMW--	66	0	4	B:53	TRANSMEM:	0.50				
---FWMWV-	77	0	8	B:64	TRANSMEM:	0.50				
--WWIKA--	51	0	7	B:45	STRAND:	0.29				
---WIAAW-	49	0.04	13	B:39	DOMAIN:	0.31	TRANSMEM:	0.31	TOPO_DOM:	0.31
--LCHYW--	52	0	0	B:4						
---CHYWK-	55	0	0	B:2						
--PRHYW--	67	0	4	B:9	REGION:	0.25	TRANSMEM:	0.25		
--ANHWM--	52	0	11	B:49						
-GENHW---	54	0	6	B:47						
--MMVWC--	88	0	2	B:5						
--HAVWC--	48	0	2	B:38						
--WWFWD--	117	0	15	B:87	TRANSMEM:	0.27				
--WWVWD--	91	0	9	B:82	TOPO_DOM:	0.44				
--HGCCH--	58	0	7	B:52	STRAND:	0.29				
--WMYIR--	46	0	26	B:46						
--SHIWY--	50	0	61	B:47						
--WPMKH--	51	0	0	B:2						
--TWYCH--	64	0	0	B:4						
--WHKRP--	55	0	0	B:6						
--IEAHW--	73	0	27	B:58						
--WQGQC--	64	0	7	B:64	ZN_FING:	0.43				
--NFWQM--	79	0	53	B:78						
--YWGCP--	74	0	35	B:71						
--RFIWW--	49	0	11	B:33	TRANSMEM:	0.45	TOPO_DOM:	0.45		
--WFPKM--	66	0	33	B:45	TRANSMEM:	0.27				
--WGIMH--	60	0	8	B:55	TRANSMEM:	0.50				
--FMWDY--	45	0	6	B:44						
--IYWHF--	59	0	29	B:52	DOMAIN:	0.31	DNA_BIND:	0.31		
--YWMHN--	86	0	84	B:84						
--HWDHY--	49	0	71	B:43	REGION:	0.49				
--TMWRG--	98	0	17	B:80						
--WMAMN--	65	0	20	B:64						
--TCHYM--	44	0	6	B:44						
--EMWQF--	43	0	6	B:43						
--EHMCM--	43	0	51	B:41	DISULFID:	0.49				
--WDMNF--	71	0	8	B:44						
--WQWRI--	43	0	0	B:23						
--IMTWM--	190	0	0	B:7						
--EHWHT--	74	0	36	B:52						
--GHWYF--	75	0	36	B:66						
--RCWHY--	53	0	78	B:52	METAL:	0.50				
--MTAWH--	80	0	0	B:9						
--MVPMW--	52	0	14	B:47	TRANSMEM:	0.50				
--HWIWQ--	64	0	9	B:43	HELIX:	0.22				
--WHPEW--	54	0	18	B:45	ACT_SITE:	0.44	DOMAIN:	0.22		
--IVHAW--	45	0.01	2	B:42	TOPO_DOM:	0.50				
--WYVVH--	96	0	10	B:85						
--MWPVH--	68	0	3	B:65						
--EFWCR--	109	0	4	B:8						
--RHWMI--	77	0	1	B:9						
--WNYWV--	54	0	19	B:32	TRANSMEM:	0.42				
--YPCNY--	66	0	24	B:65						
--WFAWR--	47	0	12	B:38	TRANSMEM:	0.50				
--IRWQH--	77	0	4	B:41	DOMAIN:	0.25				
--MGKWL--	50	0	6	B:42						
--FWWNP--	75	0	4	B:75	TURN:	0.25	HELIX:	0.25		
--WRNAW--	62	0	0	B:28						

E

--CFWEY--	1106	0	0	E:6				
KFCFW----	1086	0	10	E:14	ZN_FING:	0.50		
--NMYHC--	373	0	0	E:5				
--NWYHY--	898	0	3	E:4	HELIX:	0.33		
--CCMPF--	470	0	6	E:15	TRANSMEM:	0.50		
NGCCM----	465	0	0	E:8				
--SMFEC--	394	0	1	E:11				
---MFGCP-	558	0	4	E:9	DOMAIN:	0.25	COMPBIAS:	0.25
--ECKQC--	10768	0	176	E:34	ZN_FING:	0.71		
-FECKQ---	796	0	12	E:28	ZN_FING:	0.33		
PHECK----	527	0	14	E:20	ZN_FING:	0.50		
--QLMCC--	457	0	2	E:10				
---LMCCY-	467	0	6	E:8	PROPEP:	0.67		
-CQLMC---	449	0	1	E:6				
--CCPCN--	379	0	2	E:7	TOPO_DOM:	0.50		
---CPCNK-	405	0	39	E:22	LIPID:	0.36	COMPBIAS:	0.26
---CYCNV-	470	0	9	E:20	TOPO_DOM:	0.44		
--KHCMK--	952	0	0	E:9				
--NHCGK--	691	0	23	E:24	ZN_FING:	0.43		
--KPYNC--	468	0	72	E:32	ZN_FING:	0.51		
--KPYTC--	1415	0	209	E:30	ZN_FING:	0.52		
--KPHKC--	1616	0	218	E:36	ZN_FING:	0.48		
--INCYW--	931	0	0	E:4				
----CYWSD	386	0	1	E:7				
--KCVWQ--	691	0	3	E:9	TOPO_DOM:	0.33		
--ECVWQ--	839	0	3	E:22				
---CVWQI-	1545	0	2	E:29				
--KCVWE--	430	0	3	E:10	DISULFID:	0.33	DOMAIN:	0.33
--YECNH--	464	0	8	E:18	ZN_FING:	0.50		
--YECNQ--	2936	0	43	E:18	ZN_FING:	0.56		
--YECQQ--	385	0	9	E:19	ZN_FING:	0.44		
-PYECH---	844	0	57	E:26	ZN_FING:	0.51		
--PKYCI--	643	0	10	E:20				
-MPRYC---	384	0	121	E:30	DOMAIN:	0.36	TOPO_DOM:	0.21
--CQPSC--	387	0	107	E:28	REPEAT:	0.32	REGION:	0.24
--CQPCF--	379	0	2	E:22	DOMAIN:	0.50		
--YHCFH--	403	0	7	E:19				
---HCFHQ-	378	0	0	E:9				
---HCNHK-	1899	0	8	E:12	ZN_FING:	0.25		
--PYKCQ--	456	0	60	E:31	ZN_FING:	0.50		
--PYKCT--	1038	0	124	E:32	ZN_FING:	0.47		
--MYKCM--	536	0	0	E:3				
--RNMCP--	788	0	1	E:8				
---NMCPF-	795	0	1	E:4				
--KQTWC--	427	0	3	E:4				
---QTWCY-	433	0	4	E:12	REPEAT:	0.50		
--SKFWY--	520	0	15	E:24				
-RSKFW---	430	0	0	E:8				
--MCCYC--	470	0	1	E:16	TOPO_DOM:	1.00		
---CCYCN-	466	0	1	E:8				
--PYQCK--	933	0	113	E:29	ZN_FING:	0.50		
--PYVCK--	721	0	122	E:31	ZN_FING:	0.27		
--YNCWT--	399	0	0	E:3				
-SYNCW---	397	0	0	E:3				
--MYCEA--	435	0	6	E:15	DOMAIN:	0.50		
-TMYCE---	411	0	2	E:4	REGION:	0.50		
--IMCEV--	400	0	0	E:11				
--AWTWN--	436	0	3	E:3	REGION:	0.33	CARBOHYD:	0.33
--HMQDC--	580	0	10	E:8	DOMAIN:	0.30	TOPO_DOM:	0.30
--CWTSH--	396	0	0	E:9				
--YCQEY--	1090	0	0	E:12				
--YHEWT--	788	0	2	E:3	REGION:	1.00		
--QNVMC--	710	0	0	E:5				
--QCYYN--	414	0	21	E:23				
--MIKCY--	543	0	1	E:10				
--WSQCS--	370	0	92	E:33	DOMAIN:	0.37		
--CYIFM--	766	0	0	E:5				
--WWDHF--	569	0	0	E:3				
--MCVDY--	1094	0	0	E:10				
--AFKCM--	372	0	7	E:24	ZN_FING:	0.29		
--WGQNC--	544	0	22	E:17	CARBOHYD:	0.32		
--HNHPC--	379	0	8	E:10	DNA_BIND:	0.38		
--FYWKH--	1004	0	2	E:5				
--GFHCN--	1678	0	0	E:8				
--HKMYM--	439	0	0	E:2				
--HHCPW--	535	0	222	E:48	ZN_FING:	0.37		
--CRRCW--	577	0	2	E:12	ZN_FING:	0.50		
--FRCQW--	428	0	2	E:5	DOMAIN:	0.50		
--FTCSC--	462	0	70	E:32	DISULFID:	0.44		
--WMVCY--	537	0	0	E:1				
--WCSVK--	377	0	10	E:18				
--HNFCR--	382	0	111	E:27	DISULFID:	0.32		
--YWEFQ--	798	0	2	E:6				
--WYNEC--	802	0	2	E:5	DOMAIN:	0.50		
--CKVQW--	561	0	0	E:6				
--CMNGG--	814	0	87	E:33	DISULFID:	0.46		
--PFQCH--	373	0	11	E:28	ZN_FING:	0.45		
--FCQVR--	445	0	6	E:16				
--CMEQR--	381	0	13	E:12				
--CKQDV--	633	0	15	E:23	COILED:	0.27		
--KCCNH--	390	0	34	E:44				
--NTWHR--	414	0	8	E:12	TOPO_DOM:	0.38	DISULFID:	0.25
--CKPPN--	468	0	2	E:18	DOMAIN:	0.50	TOPO_DOM:	0.50
--MWIPK--	763	0	1	E:4				
--WWVSM--	428	0	1	E:2				
--CTHCM--	449	0	0	E:10				
--WGCFD--	379	0	50	E:41	REGION:	0.48		
--CCLMY--	519	0	49	E:10				
--CWFAN--	1120	0	9	E:13	TRANSMEM:	0.44		
--PCGHN--	403	0	15	E:32	ZN_FING:	0.47		

Genome

URP

A

--NDNAP---	3470	0.05	720	B:72	E:40	DOMAIN:	0.66	TOPO_DOM:	0.64		
DVNDN-----	3865	0.01	691	B:63	E:44	DOMAIN:	0.61	TOPO_DOM:	0.58		
--HQRVH---	5043	0.97	529	B:34	E:35	ZN_FING:	0.62				
--HQRIH---	29728	0.97	1142	B:18	E:30	ZN_FING:	0.72				
--HQRTH---	7400	0.98	717	B:13	E:33	ZN_FING:	0.68				
--QHQRI---	4777	0.91	295	B:33	E:33	ZN_FING:	0.61				
--VHQRI---	5887	0.43	206	B:40	E:32	ZN_FING:	0.39				
--RHQRI---	3379	0.44	244	B:57	E:36	ZN_FING:	0.54				
--KHQRI---	3058	0.50	167	B:22	E:33	ZN_FING:	0.47				
--HRRIH---	2262	0.50	264	B:27	E:41	ZN_FING:	0.50				
--HKRIH---	3113	0.58	326	B:15	E:40	ZN_FING:	0.67				
--HERIH---	2316	0.39	211	B:41	E:39	ZN_FING:	0.51				
--HTHTH---	2953	0.83	36	B:53	E:41	TOPO_DOM:	0.22				
---THTHT--	3725	0.81	60	B:48	E:41	METAL:	0.23				
-----THTGE	13209	0.05	1644	B:63	E:44	ZN_FING:	0.65				
--QCGKA---	9185	0.80	335	B:14	E:31	ZN_FING:	0.63				
-KQCGK----	12063	0.73	230	B:18	E:32	ZN_FING:	0.68				
-NQCGK----	4460	0.93	109	B:18	E:23	ZN_FING:	0.50				
--QCSKC---	2311	1.00	36	B:8	E:35	ZN_FING:	0.25				
--ECNQC---	3534	1.00	84	B:5	E:26	ZN_FING:	0.51				
--ECKQC---	10770	1.00	176	B:2	E:34	ZN_FING:	0.71				
---CKQCG--	12324	1.00	249	B:24	E:38	ZN_FING:	0.66				
--FKTYD---	2244	0.06	21	B:41	E:25						
----TYDNF-	2230	0.15	7	B:31	E:26						
--PTQQP---	2248	0.48	38	B:50	E:39						
---QQQPP--	2472	0.98	530	B:68	E:47	COMPBIAS:	0.44				
-TPQQQ----	2214	0.83	105	B:116	E:47						
--LTQHQ---	2505	0.87	221	B:50	E:36	ZN_FING:	0.54				
--LIQHQ---	2660	0.68	272	B:43	E:39	ZN_FING:	0.60				
--QQQHQ---	2837	1.00	488	B:48	E:47	COMPBIAS:	0.44				
--QHQQQ---	2624	1.00	438	B:55	E:48	COMPBIAS:	0.48				
-QQHQQ----	3019	1.00	451	B:68	E:47	COMPBIAS:	0.46				
--KPYQC---	3128	1.00	383	B:9	E:30	ZN_FING:	0.53				
--KPYVC---	2264	0.73	337	B:11	E:37	ZN_FING:	0.59				
--KPYKC---	15510	0.55	1361	B:9	E:41	ZN_FING:	0.72				
--KPFEC---	4830	0.66	329	B:15	E:37	ZN_FING:	0.57				
--KPFKC---	4298	0.60	471	B:4	E:45	ZN_FING:	0.51				
--PQGPP---	2458	0.19	417	B:51	E:45	REGION:	0.40				
--LPGPP---	3423	0.00	407	B:135	E:42	REGION:	0.43				
--CGKVF---	2889	0.46	351	B:19	E:39	ZN_FING:	0.51				
--CGKCF---	2304	0.92	271	B:11	E:36	ZN_FING:	0.63				
--CGKGF---	4524	0.46	654	B:20	E:42	ZN_FING:	0.62				
--QCGKT---	8630	0.85	133	B:27	E:33	ZN_FING:	0.32				
--ECGKT---	5170	0.25	476	B:23	E:36	ZN_FING:	0.54				
--YECKE---	3859	0.29	422	B:13	E:33	ZN_FING:	0.71				
--YECNE---	3964	0.52	291	B:7	E:28	ZN_FING:	0.53				
--YKCEE---	2687	0.29	400	B:13	E:32	ZN_FING:	0.77				
--QQQQH---	4655	1.00	842	B:54	E:48	COMPBIAS:	0.51				
-HQQQQ----	3595	1.00	697	B:69	E:46	COMPBIAS:	0.52				
--QQQQR---	2697	-	459	B:115	E:50	COMPBIAS:	0.32				
-LQQQQ----	6485	0.35	1061	B:132	E:48	COMPBIAS:	0.45				
--IQQQK---	2322	0.66	52	B:52	E:44						
--DVDEC---	2640	0.12	727	B:36	E:37	DOMAIN:	0.37	DISULFID:	0.36		
--DINEC---	2980	0.31	546	B:19	E:40	DOMAIN:	0.36	DISULFID:	0.36		
--KPYEC---	28764	0.67	1400	B:6	E:39	ZN_FING:	0.75				
---PYECS--	3027	0.71	308	B:12	E:33	ZN_FING:	0.61				
---PYECN--	7922	0.91	405	B:5	E:30	ZN_FING:	0.60				
--RPYEC---	3089	0.69	259	B:17	E:30	ZN_FING:	0.65				
--YECKQ---	9596	0.91	162	B:4	E:20	ZN_FING:	0.72				
--YECNQ---	2938	0.98	43	B:2	E:18	ZN_FING:	0.56				
--YKCNE---	3396	0.63	270	B:6	E:36	ZN_FING:	0.64				
-PYKCN----	5211	0.85	334	B:3	E:33	ZN_FING:	0.64				
--RPYKC---	2228	0.80	303	B:10	E:41	ZN_FING:	0.52				
---PYKCE--	3166	0.67	459	B:5	E:30	ZN_FING:	0.74				
--MWASP---	2204	0.80	5	B:19	E:16	SIGNAL:	0.40				
-AMWAS----	2201	0.60	4	B:21	E:18	VAR_SEQ:	0.50	SIGNAL:	0.25		
--HQKIH---	3863	0.96	341	B:11	E:36	ZN_FING:	0.56				
---QKIHT--	2714	0.55	246	B:28	E:32	ZN_FING:	0.55				
--FQNRR---	2717	0.54	1320	B:68	E:45	DNA_BIND:	0.48				
-WFQNR----	2579	1.00	1305	B:5	E:42	DNA_BIND:	0.49				
--TPMYF---	3471	0.59	489	B:13	E:22	TRANSMEM:	0.39	TOPO_DOM:	0.21		
---PMYFF--	4434	0.67	574	B:10	E:25	TRANSMEM:	0.47				
--HTPMY---	3429	0.91	585	B:5	E:21	TRANSMEM:	0.39	TOPO_DOM:	0.22		
-LHTPM----	3485	0.65	596	B:40	E:31	TRANSMEM:	0.38	TOPO_DOM:	0.22		
--QRTHT---	6028	0.72	585	B:77	E:33	ZN_FING:	0.64				
--QRVHT---	4244	0.58	415	B:31	E:34	ZN_FING:	0.62				
--NPAQQ---	2468	0.58	57	B:82	E:41	REGION:	0.21				
-QNPAQ----	2434	0.58	39	B:65	E:33	COMPBIAS:	0.26	REGION:	0.23		
--WMAPE---	2241	0.78	694	B:15	E:47	DOMAIN:	0.39				
--YMAPE---	2306	0.16	549	B:92	E:49	DOMAIN:	0.43				
--EIQQQ---	2480	0.50	88	B:98	E:44						
-PEIQQ----	2287	0.35	50	B:114	E:42						
--CVCVC---	2711	0.94	16	B:4	E:36	REPEAT:	0.94	TRANSMEM:	0.31	REGION:	0.31
---VCVCV--	2920	0.77	29	B:15	E:43	REPEAT:	0.52	TRANSMEM:	0.31	REGION:	0.21
--PYECK---	14414	0.67	583	B:5	E:27	ZN_FING:	0.77				
--PYKCK---	2845	0.55	288	B:10	E:32	ZN_FING:	0.62				
--CANSS---	2280	0.49	43	B:31	E:35	CARBOHYD:	0.28	TOPO_DOM:	0.21		
--ADKNH---	2284	0.33	10	B:37	E:28						
--NGGTC---	3956	0.50	1020	B:30	E:36	DISULFID:	0.56	DOMAIN:	0.27		
--QLQNQ---	2228	0.59	66	B:98	E:47						
--TAGQE---	3116	0.00	1242	B:112	E:49	NP_BIND:	0.43				
--SSRSH---	2255	0.18	309	B:71	E:49	DOMAIN:	0.44				
--YVAIC---	4163	0.39	703	B:24	E:32	TOPO_DOM:	0.33	DISULFID:	0.31		
--LAVHQ---	4026	0.13	39	B:97	E:40						
--QQKLT---	2346	0.21	56	B:112	E:46						
--QPTVQ---	3916	0.49	31	B:60	E:37						
--LHYAA---	2312	0.05	189	B:128	E:47	REPEAT:	0.43				
--HRDLK---	6019	0.05	2218	B:126	E:50	DOMAIN:	0.32	ACT_SITE:	0.32		
--WGTVC---	2241	0.99	245	B:6	E:33	DOMAIN:	0.40	DISULFID:	0.40		
--TAAHC---	2387	0.76	1538	B:79	E:33	DISULFID:	0.36	ACT_SITE:	0.23	DOMAIN:	0.23

B

--CWFAN--	1120	0.09	9	E:13	TRANSMEM:	0.44		
--CAFMN--	418	0.01	1	E:7				
--HCNHK--	1899	0.24	8	E:12	ZN_FING:	0.25		
GFHCN----	1678	0.05	0	E:8				
--MFGCP--	559	0.01	4	E:9	COMPBIAS:	0.25	DOMAIN:	0.25
-DMKGC---	653	0	10	E:22				
--KMCGK--	544	0	15	E:27	DOMAIN:	0.33		
--NHCGK--	691	0.01	23	E:24	ZN_FING:	0.43		
--PYKCQ--	457	0.04	60	E:31	ZN_FING:	0.50		
--PYKCT--	1038	0.01	124	E:32	ZN_FING:	0.47		
--PFKCD--	690	0	92	E:38	ZN_FING:	0.37		
-KPHKC---	1616	0.04	218	E:36	ZN_FING:	0.48		
--PYQCK--	933	0.04	113	E:29	ZN_FING:	0.50		
--PYVCK--	721	0	122	E:31	ZN_FING:	0.27		
--PYECH--	844	0.06	57	E:26	ZN_FING:	0.51		
--PYECQ--	859	0	76	E:25	ZN_FING:	0.49		
--KPYNC--	468	0.03	72	E:32	ZN_FING:	0.51		
--KPYTC--	1415	0.01	209	E:30	ZN_FING:	0.52		
--CYGNC--	636	0.28	2	E:11	HELIX:	0.50		
-QCYYN---	414	0.13	21	E:23				
--VEWYH--	928	0	10	E:14	DISULFID:	0.30	DOMAIN:	0.30
---NWYHY-	898	0.22	3	E:4	HELIX:	0.33		
--QLMCC--	457	0.16	2	E:10				
---LMCCY-	467	0.35	6	E:8	PROPEP:	0.67		
----MCCYC	470	0.97	1	E:16	TOPO_DOM:	1.00		
--CFWEY--	1106	0.41	0	E:6				
-KCVWE---	430	0.06	3	E:10	DISULFID:	0.33	DOMAIN:	0.33
--CCMPF--	471	0.54	6	E:15	TRANSMEM:	0.50		
NGCCM----	465	0.35	0	E:8				
--CCLMY--	519	0.35	49	E:10				
--CQLMC--	449	0.16	1	E:6				
--CYCNV--	470	0.31	9	E:20	TOPO_DOM:	0.44		
-CCYCN---	466	0.93	1	E:8				
--CPCNK--	405	0.20	39	E:22	LIPID:	0.36	COMPBIAS:	0.26
--YECNH--	464	0.11	8	E:18	ZN_FING:	0.50		
--YECNQ--	2936	0.01	43	E:18	ZN_FING:	0.56		
-CYSCN---	569	0.30	9	E:17	TOPO_DOM:	0.33	DISULFID:	0.22
--PFECN--	2156	0.00	95	E:25	ZN_FING:	0.51		
--PHECK--	527	0.01	14	E:20	ZN_FING:	0.50		
--ECKQC--	10768	0.08	176	E:34	ZN_FING:	0.71		
--ECKHC--	1456	0.29	22	E:30	ZN_FING:	0.41		
-FECKQ---	796	0	12	E:28	ZN_FING:	0.33		
--ECKYF--	588	0	3	E:19	METAL:	0.33		
--MIKCY--	543	0.03	1	E:10				
--MYKCM--	537	0.26	0	E:3				
--CTHCM--	449	0.77	0	E:10				
---KHCMK-	952	0.08	0	E:9				
--MYCEA--	435	0.01	6	E:15	DOMAIN:	0.50		
-TMYCE---	412	0.04	2	E:4	REGION:	0.50		
--RNMCP--	788	0.04	1	E:8				
---NMCPF-	795	0.12	1	E:4				
--KQTWC--	427	0.24	3	E:4				
---QTWCY-	433	0.43	4	E:12	REPEAT:	0.50		
--SKFWY--	520	0	15	E:24				
-RSKFW---	430	0	0	E:8				
--KCVWQ--	691	0.18	3	E:9	TOPO_DOM:	0.33		
--ECVWQ--	839	0.05	3	E:22				
---CVWQI-	1545	0.07	2	E:29				
--FTCSC--	462	0.09	70	E:32	DISULFID:	0.44		
--YTCSC--	500	0.11	135	E:30	DISULFID:	0.44	DOMAIN:	0.22
--NVMCH--	710	0.19	0	E:7				
-QNVMC---	710	0.03	0	E:5				
--AWTWN--	436	0	3	E:3	REGION:	0.33	CARBOHYD:	0.33
--INCYW--	931	0.34	0	E:4				
--CSKTC--	470	0.04	107	E:35	DOMAIN:	0.30		
--ECNKC--	629	0.09	79	E:29	ZN_FING:	0.35		
--YCQEY--	1090	0.03	0	E:12				
--YHEWT--	788	0.02	2	E:3	REGION:	1.00		
--CMSNK--	551	0.01	28	E:18	DISULFID:	0.50		
--CYIFM--	766	0.04	0	E:5				
--WWDHF--	570	0.26	0	E:3				
--MCVDY--	1094	0.03	0	E:10				
--WGQNC--	545	0.22	22	E:17	CARBOHYD:	0.32		
--HMQDC--	580	0.22	10	E:8	DOMAIN:	0.30	TOPO_DOM:	0.30
--FYWKH--	1004	0.08	2	E:5				
--HKMYM--	439	0.07	0	E:2				
--HHCPW--	535	0.68	222	E:48	ZN_FING:	0.37		
--CRRCW--	577	0.28	2	E:12	ZN_FING:	0.50		
--FRCQW--	428	0.22	2	E:5	DOMAIN:	0.50		
--WMVCY--	537	0.51	0	E:1				
--FWYYA--	439	0	4	E:15				
--MGCFK--	444	0.01	1	E:13	SIGNAL:	1.00		
--YWEFQ--	800	0	2	E:6				
--WYNEC--	802	0.39	2	E:5	DOMAIN:	0.50		
--CKVQW--	561	0.18	0	E:6				
--CMNGG--	814	0	87	E:33	DISULFID:	0.46		
--FCQVR--	445	0	6	E:16				
--CKQDV--	634	0	15	E:23	COILED:	0.27		
--NTWHR--	414	0.01	8	E:12	TOPO_DOM:	0.38	DISULFID:	0.25
--CKPPN--	469	0.01	2	E:18	DOMAIN:	0.50	TOPO_DOM:	0.50
--CKCHG--	437	0.20	117	E:28				
--MWIPK--	763	0.01	1	E:4				
--PKYCI--	644	0	10	E:20				
--HNHFM--	804	0.09	0	E:7				
--YHCFH--	403	0.46	7	E:19				
--WWVSM--	428	0.03	1	E:2				
--YRCEC--	579	0.12	182	E:28	DISULFID:	0.54	DOMAIN:	0.27
--HFNFC--	415	0.19	0	E:8				
--PCGHN--	403	0.02	15	E:32	ZN_FING:	0.47		
--KFCFW--	1086	0.30	10	E:14	ZN_FING:	0.50		

E

--PRHYW--	67	0	4	A:0	B:9	E:0	REGION:	0.25	TRANSMEM:	0.25		
--PFHMW--	110	0.05	15	A:5	B:95	E:1						
--EWGWQ--	55	0	6	A:1	B:40	E:1						
--HWIWQ--	64	0.17	9	A:0	B:43	E:0	HELIX:	0.22				
--FGNWH--	65	0	1	A:1	B:12	E:2						
--HYNWH--	216	0.12	18	A:0	B:9	E:0	DOMAIN:	0.44				
--YWMHN--	86	0.14	84	A:0	B:84	E:1						
--GWMHD--	110	0.01	124	A:2	B:100	E:2						
--MMVWC--	88	0.18	2	A:0	B:5	E:1						
--PMVWR--	54	0	7	A:0	B:43	E:2	REGION:	0.29				
--EHWHT--	74	0.01	36	A:0	B:52	E:1						
----WHTVY	62	0	4	A:3	B:16	E:2						
--GMNKW--	52	0	1	A:0	B:18	E:1	REGION:	1.00				
--GENHW--	54	0	6	A:0	B:47	E:2						
--WYVWA--	50	0	3	A:1	B:41	E:2	HELIX:	0.33				
--WYVVH--	96	0	10	A:0	B:85	E:2						
--YRYAW--	52	0	14	A:1	B:32	E:2						
--WRNAW--	62	0.01	0	A:0	B:28	E:0						
--HIAPW--	62	0	1	A:1	B:46	E:2						
--WIAAW--	49	0	13	A:0	B:39	E:2	DOMAIN:	0.31	TRANSMEM:	0.31	TOPO_DOM:	0.31
--KPMWG--	70	0	13	A:1	B:64	E:2	TOPO_DOM:	0.31				
-MVPMW---	52	0.01	14	A:0	B:47	E:1	TRANSMEM:	0.50				
--HGCCH--	58	0	7	A:0	B:52	E:2	STRAND:	0.29				
--HDWCH--	55	0.15	0	A:0	B:1	E:1						
--WAWGH--	49	0.01	17	A:0	B:44	E:2	METAL:	0.35	CROSSLNK:	0.35	TRANSMEM:	0.24
-YWAWW---	118	0.28	21	A:1	B:90	E:1	TRANSMEM:	0.48				
--HWAYK--	78	0	16	A:0	B:76	E:2						
--WWAYY--	70	0.10	8	A:0	B:60	E:2	TRANSMEM:	0.50				
--MTAWH--	81	0.01	0	A:1	B:9	E:1						
--MNAWA--	57	0	4	A:0	B:46	E:1						
--QWAYA--	133	0	15	A:2	B:37	E:2						
--QWWYG--	102	0.09	6	A:0	B:88	E:2						
--WMPSW--	53	0.02	5	A:0	B:46	E:1						
--WWISW--	69	0.15	8	A:0	B:61	E:1	TRANSMEM:	0.50				
--ANHWM--	52	0.02	11	A:0	B:49	E:2						
--AWRWM--	60	0.04	15	A:1	B:49	E:2	TRANSMEM:	0.27	TOPO_DOM:	0.27		
--YWQVY--	58	0	1	A:0	B:48	E:2						
-FGWQV---	55	0	3	A:0	B:45	E:2						
--WWFWD--	117	0.28	15	A:0	B:87	E:1	TRANSMEM:	0.27				
--WWVWD--	91	0.21	9	A:0	B:82	E:2	TOPO_DOM:	0.44				
-GWWHW---	50	0.47	6	A:0	B:42	E:0	STRAND:	0.33				
--WWIKA--	51	0	7	A:0	B:45	E:1	STRAND:	0.29				
AMWWI----	54	0.05	8	A:0	B:49	E:1	TRANSMEM:	0.38				
--IFWMW--	67	0.12	4	A:1	B:53	E:1	TRANSMEM:	0.50				
---FWMWV-	78	0.07	8	A:1	B:64	E:1	TRANSMEM:	0.50				
--FCDWY--	140	0.02	114	A:0	B:138	E:2						
--YCDWY--	57	0.08	34	A:0	B:57	E:2						
--GTDWY--	50	0	2	A:2	B:36	E:2						
--WHIWW--	61	0.66	11	A:0	B:53	E:1	TOPO_DOM:	0.27				
--SHIWY--	51	0	61	A:1	B:47	E:1						
--WMWPN--	70	0.24	0	A:0	B:65	E:0						
--WMAMN--	65	0.02	20	A:0	B:64	E:2						
--IRWQH--	77	0	4	A:0	B:41	E:1	DOMAIN:	0.25				
--IEWQA--	54	0	13	A:3	B:40	E:2	STRAND:	0.23				
--MAHDW--	67	0.02	1	A:0	B:12	E:1						
---AHDWC-	60	0	1	A:0	B:6	E:1						
--WWNPD--	93	0.01	5	A:3	B:83	E:1						
-FWWNP---	75	0.03	4	A:0	B:75	E:1	TURN:	0.25	HELIX:	0.25		
--LCHYW--	52	0.01	0	A:0	B:4	E:0						
---CHYWK-	55	0.08	0	A:0	B:2	E:1						
--ACMHC--	55	0.01	12	A:0	B:47	E:1						
--HNWPG--	72	0	0	A:0	B:52	E:2						
--WPMKH--	51	0.02	0	A:0	B:2	E:1						
--TWYCH--	64	0.12	0	A:0	B:4	E:1						
--TWWSY--	50	0	0	A:1	B:48	E:2						
--FPMDW--	56	0	0	A:0	B:7	E:1						
--WHKRP--	55	0	0	A:0	B:6	E:1						
--IEAHW--	73	0	27	A:0	B:58	E:1						
--GWFIE--	64	0	9	A:2	B:52	E:2	TRANSMEM:	0.22				
--WQGQC--	64	0	7	A:0	B:64	E:1	ZN_FING:	0.43				
--NFWQM--	79	0.01	53	A:0	B:78	E:2						
--YWGCP--	74	0	35	A:0	B:71	E:0						
--WFPKM--	66	0	33	A:0	B:45	E:2	TRANSMEM:	0.27				
--WAMTQ--	72	0	1	A:1	B:16	E:1						
--RHWMI--	77	0.02	1	A:0	B:9	E:1						
--IYWHF--	59	0.01	29	A:0	B:52	E:1	DOMAIN:	0.31	DNA_BIND:	0.31		
--HWDHY--	49	0.08	71	A:0	B:43	E:1	REGION:	0.49				
--TMWRG--	98	0	17	A:0	B:80	E:2						
--PIFWW--	49	0.02	12	A:1	B:48	E:2	TRANSMEM:	0.33	DOMAIN:	0.33		
--WDMNF--	71	0	8	A:0	B:44	E:1						
--IMTWM--	190	0.02	0	A:0	B:7	E:0						
--TYAMW--	49	0	2	A:0	B:46	E:1						
--GHWYF--	76	0	36	A:1	B:66	E:1						
--RCWHY--	53	0.04	78	A:0	B:52	E:0	METAL:	0.50				
--WPIGC--	52	0	50	A:1	B:50	E:2						
--WGGWW--	119	0	16	A:0	B:86	E:1	TRANSMEM:	0.25				
--WHPEW--	56	0.05	18	A:2	B:45	E:0	ACT_SITE:	0.44	DOMAIN:	0.22		
--MWPVH--	68	0.01	3	A:0	B:65	E:2						
--EFWCR--	109	0	4	A:0	B:8	E:2						
--WTMRW--	75	0.1	0	A:0	B:4	E:1						
--YVFWE--	56	0	6	A:3	B:44	E:2						
--WGIMH--	60	0.02	8	A:0	B:55	E:2	TRANSMEM:	0.50				
--WNYWV--	54	0.05	19	A:0	B:32	E:2	TRANSMEM:	0.42				
--YPCNY--	66	0	24	A:0	B:65	E:2						
--HYDYW--	78	0.02	33	A:3	B:71	E:1						
--RDWWP--	61	0.01	6	A:2	B:55	E:2						
--CHDPH--	80	0	0	A:1	B:24	E:2						
--MGKWL--	50	0	6	A:0	B:42	E:1						
--GWLWF--	57	0	18	A:0	B:53	E:2						
--FYWYQ--	52	0.01	2	A:5	B:38	E:1						
